# Automated landmarking via multiple templates

**DOI:** 10.1371/journal.pone.0278035

**Published:** 2022-12-01

**Authors:** Chi Zhang, Arthur Porto, Sara Rolfe, Altan Kocatulum, A. Murat Maga

**Affiliations:** 1 Center for Development Biology and Regenerative Medicine, Seattle Children’s Research Institute, Seattle, Washington, United States of America; 2 Department of Biological Sciences, Louisiana State University, Baton Rouge, Louisiana, United States of America; 3 Center for Computation and Technology, Louisiana State University, Baton Rouge, Louisiana, United States of America; 4 Friday Harbor Laboratories, University of Washington, San Juan Island, Washington, United States of America; 5 Alfred University, Alfred, New York, United States of America; 6 Division of Craniofacial Medicine, Department of Pediatrics, University of Washington, Seattle, Washington, United States of America; Ecole Normale Supérieure de Lyon, FRANCE

## Abstract

Manually collecting landmarks for quantifying complex morphological phenotypes can be laborious and subject to intra and interobserver errors. However, most automated landmarking methods for efficiency and consistency fall short of landmarking highly variable samples due to the bias introduced by the use of a single template. We introduce a fast and open source automated landmarking pipeline (MALPACA) that utilizes multiple templates for accommodating large-scale variations. We also introduce a K-means method of choosing the templates that can be used in conjunction with MALPACA, when no prior information for selecting templates is available. Our results confirm that MALPACA significantly outperforms single-template methods in landmarking both single and multi-species samples. K-means based template selection can also avoid choosing the worst set of templates when compared to random template selection. We further offer an example of *post-hoc* quality check for each individual template for further refinement. In summary, MALPACA is an efficient and reproducible method that can accommodate large morphological variability, such as those commonly found in evolutionary studies. To support the research community, we have developed open-source and user-friendly software tools for performing K-means multi-templates selection and MALPACA.

## Introduction

Landmark data has been increasingly used in biological and biomedical fields to quantify complex phenotypes for investigating underlying genetic, environmental, mechanical, and evolutionary factors through multivariate analyses [1–7]. Nevertheless, collecting landmarks manually, nowadays mostly done by annotating high-resolution 3D images or surface models, can be laborious and time consuming [8–12]. Manual landmarking also inevitably leads to intra- and interobserver errors, which can disrupt detection of biologically meaningful variations [10, 13–16]. Nowadays, combining landmark data collected by different researchers has become increasingly common when working with big datasets, so proper error control has become more urgent for ensuring accuracy, consistency, and reproducibility [10, 13, 15–17].

To resolve the limitations in manual landmarking, researchers have developed various automated landmarking techniques based on image registration [9, 12, 18–20]. However, these methods may not always be convenient for biologists to use because most of them require high-end hardware and knowledge of image-processing [10, 12, 18, 19]. We have recently introduced an automatic landmarking method, ALPACA (Automated Landmarking through Point cloud Alignment and Correspondence), as part of the SlicerMorph morphometrics toolkit [10, 21]. For a detailed explanation of ALPACA and its underlying methods, we refer the readers to the cited papers. In summary, ALPACA is fast and lightweight because it uses sparse point clouds extracted from the 3D surface models of biological specimens [10]. These point clouds, while retaining sufficient geometric information, greatly reduce the computational burden so that ALPACA can efficiently run on any recent personal computer and does not require access to specialized hardware such as GPUs or high-performance computing cluster. ALPACA is free to download as part of the open-source SlicerMorph extension in the 3D Slicer software [10, 21, 22].

One limitation of ALPACA and most other registration-based methods lies in the usage of single template for landmarking the entire sample [10]. This is because the accuracy of automated landmarking depends on how well the registration algorithm optimizes the cost of global registration with local shape differences, which becomes more difficult as the template and target specimens become more different in form [10, 12]. This limitation is particular well-known in neuroimaging where template-based analysis had been the norm for the last two decades [12, 23–25] and is particularly difficult in evolutionary focused biological studies, where researchers frequently deal with highly variable samples from different species. Consequently, it can be difficult to use a single template to landmark a variable study sample while maintaining accuracy of landmarking.

If one template is insufficient for landmarking a highly variable study sample, a potential solution is to use multiple templates to have a more comprehensive representation of the whole study sample [12, 24–26]. The multi-template approach has already been utilized in automated image segmentation to better capture variations of anatomical structures within a sample so that the segmentation of these structures can be more accurate [12, 23–27]. Young and Maga [12] adopted the multi-template method from automated segmentation for their image-based automatic landmarking research and have achieved an improvement in accuracy comparing to the single-template method. The main issue with the multi-template methods is the increased computational demand, as each subject now needs to be registered as many times as there are templates. While this is a concern for computationally expensive methods takings hours, in ALPACA a single specimen can be landmarked in a matter of few minutes, making ALPACA an effective tool to implement a multi-template automated landmarking approach [10].

In this study, we present a multi-template, automated landmarking pipeline called MALPACA based on the methods of ALPACA. Overall, there are two steps to the MALPACA workflow: the first step is to identify the templates to be used to landmark the rest of the samples. The second step is the execution of the multi-template estimation pipeline, which is essentially running ALPACA independently for each unique template. For each of the three coordinates of a single landmark, we take the median from all corresponding estimates given by each template used in MALPACA as the final output. Therefore, multiple templates can contribute to landmarking one target specimen, depending on the performance of each template. In this way, MALPACA can avoid biases introduced by using a single template to landmark every specimen.

Identifying the templates to sufficiently capture the variations of the whole sample is critical for any method that relies on them [26, 28, 29]. This might be particularly difficult if there is no prior information, such as a pilot study with smaller samples sizes, available to the investigator. If the investigator needs to manually landmark even a portion of the study sample to determine the patterns of morphological variations and choose the ones to be used as templates, this time investment may overcome the benefit from automated landmarking. If such a pilot dataset is already in existence, we advise investigators to make use of these priors for template selection. For the cases where no prior information is available, we implemented a K-means based template selection that uses point-clouds of the surface models of the study population to approximate the patterns of overall morphological variations in an unbiased way. A Generalized Procrustes Analysis (GPA) is applied to these point clouds, followed by the PCA decomposition of Procrustes aligned coordinates. Using all PC scores from the GPA we apply K-means clustering to the data to detect the samples that are closest to the centroids of the identified clusters. The investigator can specify how many templates per group (if data consists of multiple groups or species) will be extracted. The investigator then landmarks the selected specimens, and inputs them as the templates for the MALPACA pipeline. More details of how to execute the K-means based template selection procedure can be found in the Material and Methods section and S1 Appendix.

The first goal of this study is to evaluate whether MALPACA outperforms single-template ALPACA in estimating landmark positions with less error when compared to the “gold standard” (GS) manual landmarks. The second goal is, in cases where no prior information about the patterns of variations for a sample is available, to assess whether K-means can be an acceptable alternative for choosing a set of templates for MALPACA. The results confirm our expectations that MALPACA outperforms ALPACA for both the single-population mouse and multi-species ape samples. The K-means based multi-template selection method proposed in this study can also generate a template set with good performance when researchers have no prior knowledge for optimal template selection.

An advantage of our multi-template pipeline is the ability to implement a *post-hoc* analysis for quality control. In a single template-based analysis, the only way to assess quality is to manually redo the landmarking and compare the result with the estimated landmarks, which defeats the purpose of automation. Therefore, we provide an example that shows how to import estimates for individual templates into R and conduct an analysis to assess how closely they converge. Optionally, subsets of landmark estimates can be removed to potentially improve the result. This demonstrates the flexibility and capability of developing a *post-hoc* quality check. Users are encouraged to design their own *post-hoc* analysis by setting up their own criteria for detecting outliers. For the research community, we provide modules with graphic user-interface for performing K-means multi-template selection and MALPACA (see S1 Appendix for instructions) within the SlicerMorph extension of the open-source 3D Slicer platform [21, 22]. The structure of the study is summarized in Table 1.

**Table 1:** A summary of research goals and analyses.

Research goals	Samples	Analysis and Evaluation Metric
1. Evaluate whether MALPACA outperforms ALPACA	Mouse and ape sample	1) RMSE and individual landmark errors comparing to manual landmarks (“gold standard” or GS); also comparing to intraobserver manual landmarking errors.
		2) Correlations of morphometric variables such as centroid sizes, pairwise Procrustes distances and PC scores with morphospace derived from GS
2. The performance of K-means multi-template selection	Mouse sample	Permutation test: comparing RMSEs derived from K-means based MALPCA and 100 rounds of MALPACAs, each of which based on a random combination of 7 templates
	Ape sample	Permutation test: comparing RMSEs derived from K-means based MALPCA and 50 rounds of MALPACAs using randomly selected templates, each of which based on a random combination of 6 templates
3. Examples of quality check for the performance of individual templates and refined MALPACA to show the flexibility of designing *post-hoc* analysis	Ape sample	1) Extract outlier estimated landmarks of individual templates
		2) Remove outliers and re-calculate median estimates; compute new RMSEs comparing to GS to assess the performance relative to the original MALPACA
		3) “Species-specific” MALACA as a potential improvement: one MALPACA for each ape species using the two templates from that species; comparing to the original MALPACA based on RMSEs and correlations in Procrustes distances and PC scores with GS.

For details, please see the Material and methods session

## Materials and methods

### Samples

Our study uses two samples that represent different types of group structure. Each specimen is a 3D model in the PLY format. The first sample consists of 3D skull models of 61 inbred laboratory mice from both classical and wild-derived strains [10, 20] (See S1 Table for list of strains used in the study and S1 Data for data availability). This dataset is used to mimic a population level morphometric analysis, where no distinct grouping structure exists (or expected) in the resultant morphospace. All specimens have been manually annotated with 51 landmarks once by single observer (S1A Fig; S1 Data). Throughout this study we will refer the manually annotated datasets as “gold standard” (GS).

The second sample contains 3D skull models of 52 great ape specimens from three species: 11 *Pan troglodytes*, 23 *Gorilla gorilla*, and 18 *Pongo pygmaeus* (For details, please see [10, 30], S1 Data and S2 Table for a list of the specimens used in this study). The dataset is provided by courtesy of the 3D Digitization program of the Smithsonian Institution (S1 Data). Each specimen has been annotated twice manually with 41 landmarks by a single observer (S1B Fig, S1 Data). The mean of these two manual landmark sets is used as the GS.

### K-means based methods for selecting multiple specimens as templates

This study utilizes K-means clustering as the basis for selecting specimens from the mouse and ape data sets as templates [12]. We used this K-means template selection method to choose seven templates from the mouse sample to landmark the remaining 54 specimens using MALPACA (Table 2 and S1 Table). For the ape sample, we ran a K-means template selection procedure for each species to identify two templates per species. Consequently, six templates were used to landmark the remaining 46 ape specimens (9 *Pan*, 21 *Gorilla*, and 16 *Pongo*) (Table 2 and S2 Table).

**Table 2:** K-means selected templates.

Mouse templates	Ape templates
129S1.SVIMJ	USNM084655 (*Pan troglodytes*)
B6CBAF1.J	USNM176236 (*Pan troglodytes*)
BALB.CBYJ	USNM590953 (*Gorilla gorilla*)
CAST.EIJ	USNM599167 (*Gorilla gorilla*)
SF.CamEiJ	USNM142185 (*Pongo pygmaeus*)
SPRET.EiJ	USNM153830 (*Pongo pygmaeus*)
X129P3.J	

For a full list of samples and accessing the data, see S1 Data, S1 and S2 Tables.

The input 3D models for the K-means based method is required to have a filetype that is compatible with 3D Slicer, such as PLY, OBJ, STL, and VTK [22] (also see S1 Appendix). The raw data for K-means are the full set of principal component (PC) scores derived from the decomposition of GPA aligned coordinates from sparse point clouds extracted from the input 3D models. To achieve this, point-to-point correspondences must be established across point clouds so that they can be treated as landmark configurations. This is a three-step process:

1. A reference model is tightly registered to the target(s) using the global and ICP rigid registration steps from ALPACA [10, 31–33].
2. A sparse point cloud is extracted from the reference. This is done by deleting points in the reference model until the distance between any pair of points exceeds a user-defined threshold [34]. In our sample, this leads to the reference point clouds with 791 and 674 points for the mouse and ape datasets respectively.
3. For each point in the reference point cloud, the closest point in the target model is extracted [34]. This results in a sparse point cloud from the target model that has a point-to-point correspondence with the reference point cloud.

These point clouds are treated as landmark configurations and submitted to a Generalized Procrustes Analysis (GPA) [1, 35]. GPA is the most common registration method for geometric morphometrics [1, 35]. It registers the landmark configuration of each specimen to each other by removing non-shape factors of size, orientation, and locations. After GPA alignment, only the shape information is retained. The coordinates of the GPA registered landmark sets are called shape coordinates or Procrustes aligned coordinates. We then run a Principal Component Analysis (PCA) based on the covariance matrix of the shape coordinates [1, 21, 35]. The resultant principal components (PC) scores of each specimen can account for the total variance of the shape coordinates, but with much lower dimensionality. Using the PC scores rather the original shape coordinates for statistical analysis can greatly reduce the computational time yet retain the original shape information.

The full set of PC scores are input into the K-means algorithm to select a user-defined number (k) of templates. The K-means algorithm, which is implemented using the SciPy Python package, iteratively looks for an optimal way to partition specimens into k clusters so that the mean within-cluster Euclidean distance between specimens and the centroid is minimized [36]. Within each cluster, the specimen closest to the centroid is selected as a template, so that k templates are selected [12]. Step-by-step instructions for executing K-means template selection using the mouse data and landmarks are available in S1 Appendix. Parameter settings used for K-means template selection are listed in S3 Table (also see S1 Appendix for detailed explanations).

It should be noted that for the K-means template selection procedure to work correctly, 3D models of all specimens should be complete and segmented to contain the same anatomical content. Because the workflow uses the entire model to generate point clouds, inclusion of incomplete specimens or extraneous anatomical elements will create spurious outcome.

### MALPACA process

MALPACA with K-means selected templates was used to landmark both the mouse and ape samples. To initiate MALPACA, the user simply needs to input the location of 3D models, specimens designated as templates, and landmark sets corresponding to the templates (S1 Appendix). The 3D models for the MALPACA pipeline should be in the PLY format. For each template, ALPACA is run independently to landmark the target specimens to landmark them (for details of ALPACA, please see [10]). After a target specimen is landmarked by each template, each of the final x, y, and z coordinates of every landmark on this target specimen is the median of the corresponding estimates generated by all individual templates. The three coordinates (i.e., x, y, and z) of any landmark of a target specimen may come from any of the templates. Therefore, the final MALPACA-derived estimates of a specimen can be contributed by multiple templates. Note that we take the median rather than the mean from the corresponding ALPACA-derived estimates for each landmark estimate. This is to reduce the potential skew may be created by outlier estimates generated due to various factors (registration failure, particular template and subject are too different, etc.) associated with using arithmetic means.

The MALPACA module returns both the final median landmark estimates and estimates from individual templates as files on disk. Because ALPACA registers the template to a target specimen to project template landmarks to the surface of the target, the estimated landmarks from both ALPACA and MALPACA are in the target specimen’s own coordinate system. While we chose median to estimate final landmark location due to its robustness to outliers, providing all the output gives the user flexibility to implement their own estimation methods or evaluate the performance of each template in estimating certain landmark positions on certain specimens. For example, users can extract the contribution of each template to a particular target specimen. We also provide an example to extract outlier estimates given by specific templates (see later section “*Post-hoc* quality check and refined MALPACA”). Users can opt to remove these outliers to potentially improve the final results. Step-by-step instructions on how to execute the multi-template landmarking can be found in S1 Appendix in the Supporting Information. For availability of landmarks estimated by K-means based MALPACA in this study, please also see S1 Appendix.

### Evaluating MALPACA performance

Performance evaluation of MALPACA against baseline ALPACA is done by comparing how closely the MALPACA and each template’s ALPACA-derived landmark estimates approximate our gold standard (GS), manual landmarks. This can be conveniently done because our method outputs the landmark estimates given by each selected template. All three groups of landmark coordinates (MALPACA derived, ALPACA derived and manual) are directly comparable without requiring them to be superimposed by GPA. For the mouse sample, in addition to the K-means selected templates, we acquired the synthetic template constructed by Maga et al. [20], which is the average model of the same sample used in their study. This synthetic template was also the template used in the original ALPACA evaluation study [10]. We then ran ALPACA based on this synthetic template for the mouse sample to compare with the K-means based MALPACA.

We used the root mean square error (RMSE) to quantify the overall positional deviation between two landmark sets for a given specimen. RMSE represents the square root of the mean sum of squared errors between two landmark sets as shown in the Eq (1) below.


$$RMSE\;=\sqrt{\frac{\sum_{i=1}^{N}\left[{\left({\hat{x}}_{i}-x_{i}\right)}^{2}+{\left({\hat{y}}_{i}-y_{i}\right)}^{2}+{\left({\hat{z}}_{i}-z_{i}\right)}^{2}\right]}{N}}$$ (1)

In Eq (1), *N* represents the sample size, ${\hat{x}}_{i}$, ${\hat{y}}_{i}$, and ${\hat{z}}_{i}$ represent the coordinates of the *i*th landmark in one landmark set (e.g., a MALPACA or ALPACA-derived estimated landmark set), and *x*_*i*_, *y*_*i*_ and *z*_*i*_ represents the coordinates of the *i*th landmark in another landmark set (e.g., the manual landmark set). Thus, RMSE serves as a single value to summarize the overall deviations between all estimated and GS landmarks for a specimen. The smaller the RMSE is, the less deviation it is between an estimated and manual landmark sets (or any two landmark sets) for a specimen.

The RMSEs reported are in millimeters (mm) because the landmarks are collected in coordinate systems on the scale of millimeters. We also report RMSEs as percentage of the centroid size, calculated from each specimen’s GS (manual) landmark set, so that they can be understood in context of the body size. Statistical significance of the differences between the MALPACA and ALPACA derived RMSE values is assessed via one-sided Welch t-test.

In addition to RMSEs, we compute the Euclidean distance or error between the estimates of individual landmarks and their corresponding GS landmarks as a comparison of MALPACA and ALPACA performance. One-sided Welch t-tests are used to test the number of MALPACA-GS individual landmark errors that are significantly smaller or larger than the corresponding ALPACA-GS errors. If the MALPACA-GS error for an individual landmark is significantly smaller than the ALPACA-GS errors, MALPACA outperforms ALPACA in estimating the position of this landmark.

To further evaluate the performance of MALPACA-estimated landmarks in morphometric analysis, we perform separate GPAs for MALPACA-derived estimates, ALPACA-derived estimates, and GS landmarks. We then extract size and shape variables, namely:

1. pairwise Procrustes distances,
2. principal components (PCs), and
3. centroid sizes, from each GPA-aligned landmark set.

Procrustes distances quantify overall shape differences. PCs are components ranked by proportions of the total variance they explain. Centroid sizes are common size measures for specimens based on their landmark sets. Morphometric analyses use these variables to represent overall shape and size variations [35]. We calculate correlations between estimated and GS landmarks in these size and shape variables. Joint GPA between MALPACA and GS landmarks is also carried out to visualize differences between estimated mean shapes.

### Assessing the magnitude of digitation errors in MALPACA

As indicated above, both intra and interobserver errors are common in manual landmarking [9, 13, 15, 16]. Thus, it is important to evaluate the MALPACA RMSEs in context of manual digitization errors. Our mouse sample has been landmarked only a single time, so it is not possible to calculate observer errors. Instead, we rely on intraobserver errors computed by Percival et al.’s [9] as the reference for manual errors for the thirty-four of the landmarks in their study that are replicated in our analysis. We compare the published mean intraobserver errors for these thirty-four landmarks from Percival et al. [9] with the mean MALPACA-GS and ALPACA-GS errors for the same set of landmarks in our study. No GPA alignment is needed for this comparison because Percival et al. [9] also computed intraobserver errors in their mouse specimen’s own coordinate system. For the ape sample, each specimen has been landmarked twice by the same observer. The RMSEs between the two manual landmark sets are used as an estimate of intraobserver errors. In addition, we compute Euclidean distances between corresponding landmarks from the two manual landmark sets as intraobserver error of individual landmarks.

### Robustness of K-means based template selection using permutation tests

This study uses permutation analysis to evaluate whether K-means is an acceptable method to select templates when no prior information is available, compared to randomly selecting specimens as templates (See Table 1 for the K-means selected templates for the mouse and ape samples). Each permutation is based on using a random choice of specimens as templates to run MALPACA. For the mouse sample, 100 permutations are carried out to generate 100 random combinations of seven templates. For each permutation, a MALPACA is carried out to landmark the remaining 54 mouse specimens, generating 54 RMSEs between the estimated and GS landmarks of each specimen. The mean RMSE for each MALPACA run is then calculated, resulting in 100 mean RMSEs for the permutation analysis. We then compare the mean RMSE from the mouse K-means based MALPACA with the pooled 100 mean RMSEs derived from all the permutated MALPACAs. If the mean RMSE of the K-means based MALPACA falls below the majority of the 100 mean RMSEs given by the permutated MALPACAs, the K-means based MALPACA is considered to outperform MALPACAs using randomly selected templates. In addition, we compare the RMSE for each of the 54 target specimen from the K-means based MALPACA to all 5,400 RMSEs (100 permutations × 54 target specimens) derived from the permutated MALPACAs.

For the ape sample, the same type of permutation analysis is carried out for evaluating the performance of six K-means based templates. We carry out 50 MALPACA runs based on 50 random combinations of six templates given by 50 rounds of the permutation analysis. The mean RMSE from the K-means based MALPACA is compared to the pooled 50 mean RMSEs given by the permutated MALPACAs. We then compare the RMSE of each of the 46 ape target specimens to the pooled 2,300 RMSEs (50 permutations × 46 target specimens).

The evaluation of MALPACA performance is carried out in R [37]. RMSEs of the data are calculated using the SlicerMorphR package (https://github.com/SlicerMorph/SlicerMorphR). Generalized Procrustes Analysis (GPA) is performed using the geomorph R package for calculating correlations between estimated and manually placed landmarks in centroid sizes, pairwise Procrustes distances, and principal component scores [38].

### *Post-hoc* quality check and refined MALPACA

To test whether outlier estimates given by individual templates may negatively impact the final output, we perform a *post hoc* analysis to detect and remove outliers as an attempt to further reduce the errors between the MALPACA-derived estimates and GS. In particular, this analysis is applied to the multi-species ape sample, since using the templates from one species to landmark specimens of another morphologically distinct species is more likely to generate poor landmark estimates. Including these poor results may reduce the accuracy of the final estimates. Thus, we expect the outlier extraction analysis can identify these poor estimates as outliers and remove them to enhance landmarking results. Procedures described here are available as R functions incorporated to SlicerMorphR package. See S1 Appendix for instructions on use.

To determine whether an estimated landmark on a specimen derived from a template is an outlier, we first calculate the Euclidean distance between this estimated landmark and its corresponding MALPACA-derived estimate. We then define a heuristic threshold as two standard deviations above the mean of the pooled distances between each estimated landmark from a template and its corresponding MALPACA final output for one specimen. If the distance for an estimated landmark derived from a template exceeds the threshold, this landmark is considered as an outlier. The outliers are then removed from recalculation of the median landmark positions. Accordingly, RMSEs are calculated between these new median estimates and GS landmarks to see if there is an improvement.

As an attempt to eliminate the potentially negative effect of including poor estimates from the templates of other ape species, we also design a “species-specific” approach that performs three separate MALPACA runs. Each run landmarks one species by directly calculating medians between the ALPACA estimates from the two templates of that species (“species-specific MALPACA” in the main text). RMSEs between landmarks generated by each approach and the GS are calculated to compare the performance of the species-specific MALPACA to the original MALPACA that uses results given by all templates. We also assess correlations in centroid sizes, pairwise Procrustes distances, and PC scores derived from each method.

## Results and discussion

All MALPACA analyses were run on a 64-bit Windows 10 desktop with an Intel Core i5 3.10 GHz CPU, and 16 GB RAM. All MALPACA landmark estimates rely on templates selected by the K-means multi-template method. For the mouse dataset, the execution time is 69s for a single sample using ALPACA or 7.2h for the whole sample using all seven templates, which includes the template selection. For the ape sample, the execution time is 240s for a single specimen using ALPACA or 18.4h for the whole sample using all six templates.

### MALPACA performance over ALPACA

**Fig 1:**
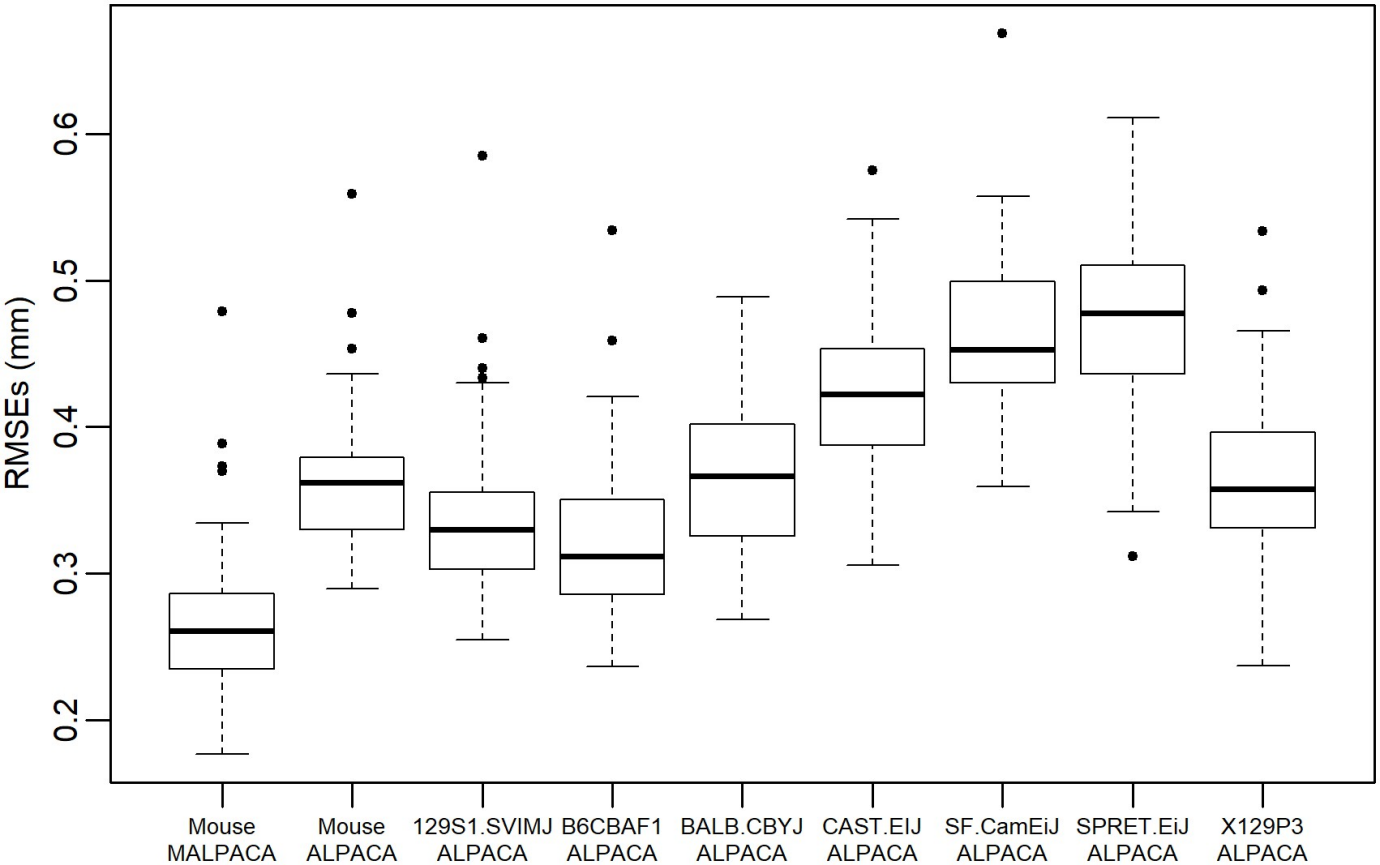
Comparison of RMSEs for the mouse sample. RMSEs calculated between estimated landmarks and the “gold standard”. Each box represents the RMSEs of 54 mouse specimens calculated by a specific analysis. “ALPACA”: Mouse ALPACA landmarks are estimated using the synthetic mouse template used in the original ALPACA paper. Other columns are ALPACA-derived estimates using each specified template. To see RMSEs expressed as percentage of specimen centroid sizes based on “Gold Standard” manual landmarks, please see S2 Fig.

**Fig 2:**
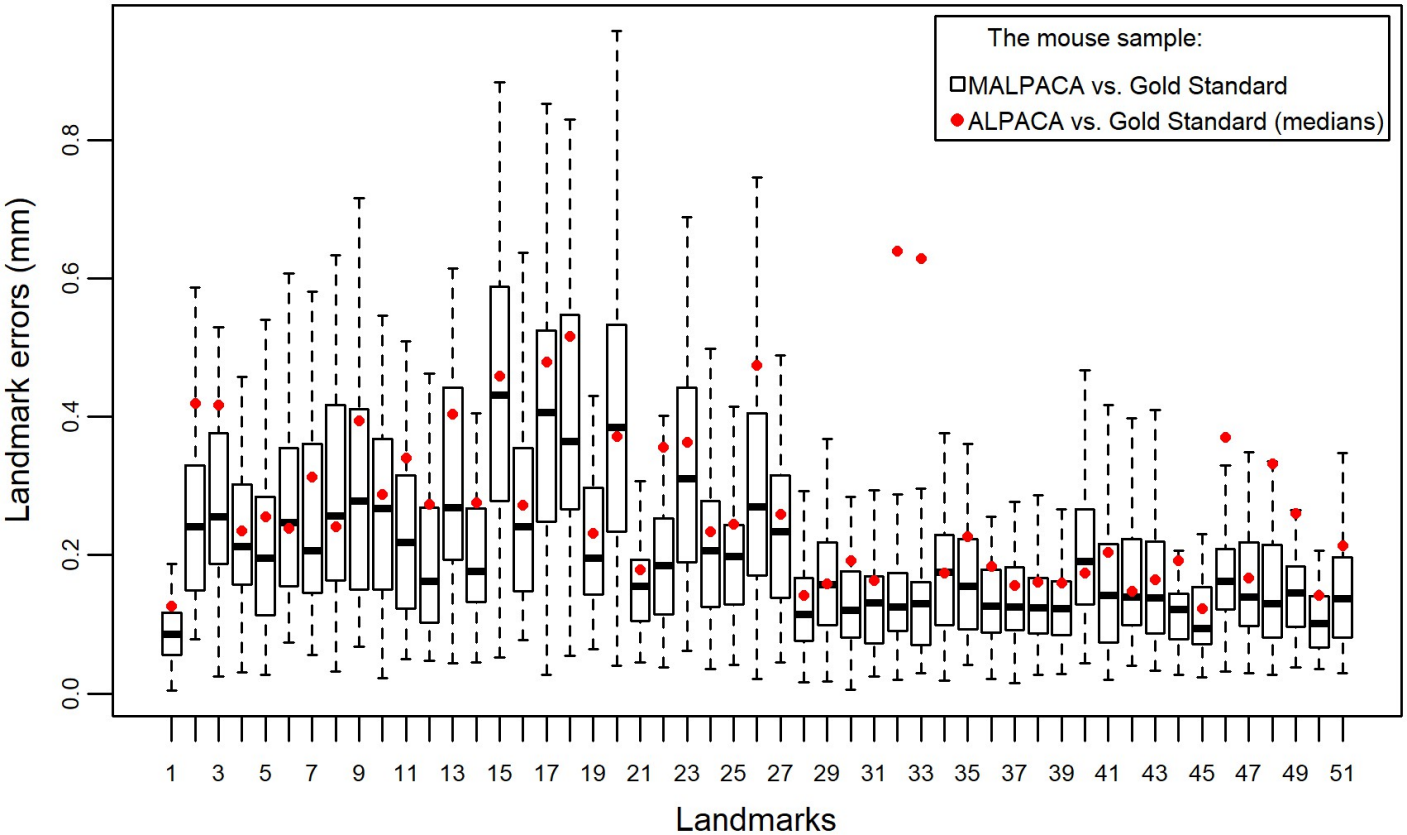
Comparison of individual landmark errors for the mouse sample. Errors represented by Euclidean distances (for errors expressed in percentage of centroid sizes, see S3 Fig). Each column represents errors between MALPACA-derived estimates of all 54 mouse specimens for one landmark and their corresponding Gold Standard (GS) landmarks. Red dots: each represents the median of errors between the estimates of the synthetic template ALPACA of all 54 mouse specimens for one landmark and their corresponding GS landmarks.

The mouse MALPACA landmarking yields significantly smaller RMSEs than those generated by ALPACA based on any K-means selected template or the synthetic template (all p-values from one-sided Welch t-tests < 8.52 × 10^−7^) (Fig 1, S2 Fig and S4 Table). For 37 out of 51 landmarks, the MALPACA-based errors are significantly smaller than the ALPACA-based errors using the synthetic template (p-values < 0.05 for one-sided t-tests for assessing if MALPACA-errors are smaller) (Fig 2, S3 Fig and S5 Table). For the remaining 14 landmarks, the MALPACA-based errors are not significantly larger than the ALPACA ones (p-values > 0.37 for one-sided Welch t-tests assessing if ALPACA-based errors are smaller) (Fig 2 and S6 Table). The joint GPA of MALPACA-based and GS landmarks yields highly consistent mean shapes (S4 Fig).

**Fig 3:**
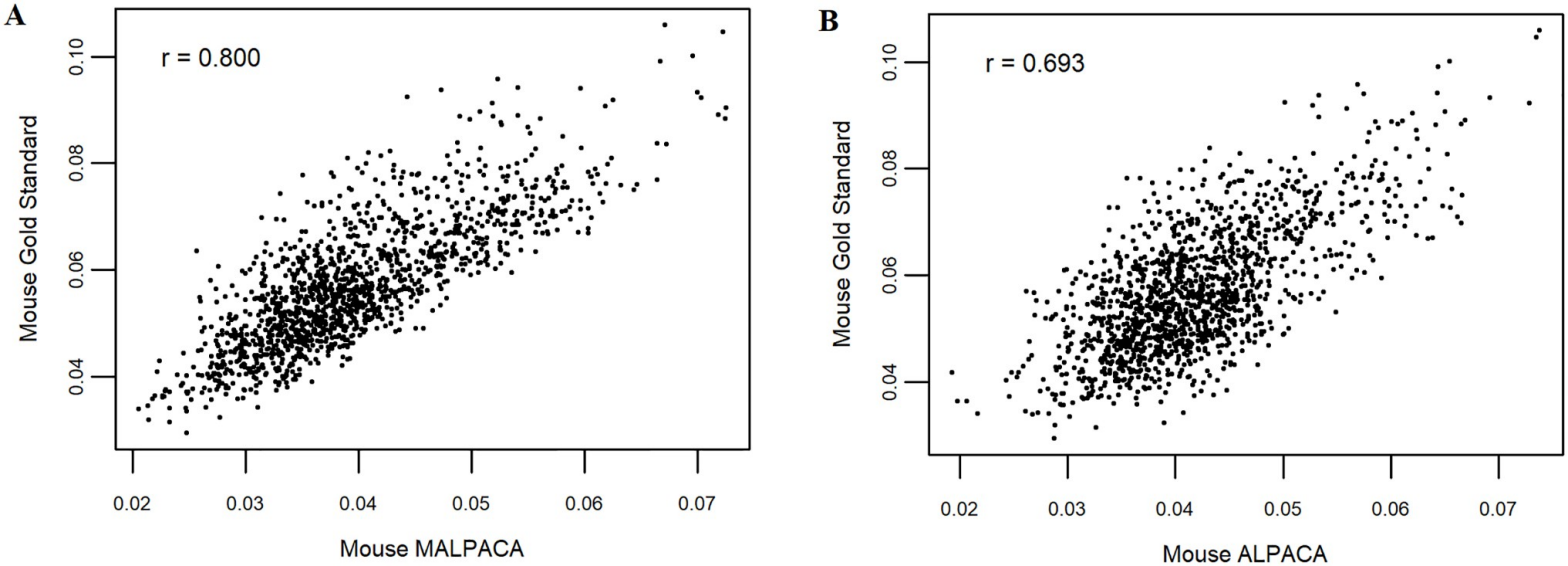
Comparison of the pairwise distances derived from the mouse estimated and GS landmarks. (A) MALPACA vs. GS. (B) ALPACA vs. GS based on the synthetic template. “r” = correlation coefficient. The plot is based on distances between all pairs of specimens within the mouse sample derived from separate GPA of estimated and GS landmarks.

To test for differences in the common morphometric variables (Procrustes distances, centroid sizes, and PC ordination scores), we have investigated correlations of these variables from the separate generalized Procrustes alignments of MALPACA-derived estimates and GS landmarks. For the mouse data, our results show that the Procrustes distances calculated from MALPACA-derived estimates and GS landmarks are highly correlated in pairwise distances with a 0.8 correlation coefficient (denoted as r) (Fig 3A). Centroid sizes calculated from MALPACA-derived estimates and GS landmarks are almost identical with the correlation coefficients exceed 0.99 (S7 Table). For PC scores, Procrustes distances, and centroid sizes, the correlations of MALPAC-derived estimates with GS exceed that of ALPACA and GS (Fig 3B; S7 Table).

**Fig 4:**
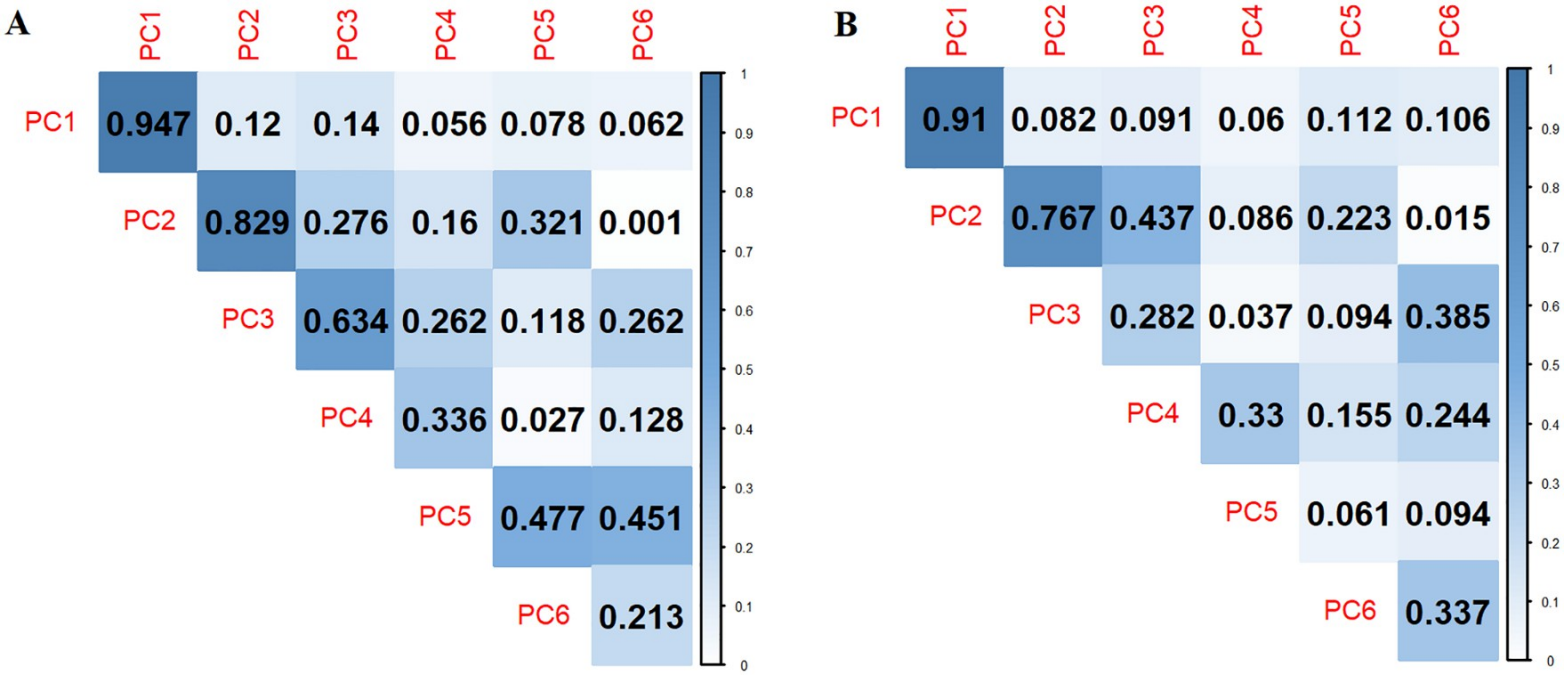
Correlations in the first six Principal Components (PCs) between estimated and GS landmarks. (A) MALPCA vs. GS. (B) ALPACA (the synthetic template) vs. GS. PC scores are derived from separate GPAs of estimated and manual landmarks. Each grid represents the correlation coefficient between a PC from an automated landmarking analysis and a PC from the GS, depending on its row and column names.

To compare the similarity of morphospaces derived from MALPACA and GS landmarks, we relied on the strength of the correlations of corresponding PC scores from different datasets. The MALPACA-GS correlation is especially strong in PC 1 scores as the correlation coefficient in PC 1 scores reaches 0.947 (Fig 4A). MALPACA-derived estimates and GS landmarks also yield high correlation in PC 2 scores (r = 0.83), with a gradual decline of correlation coefficients with the other PC scores. The ALPACA-GS correlations in scores of PC 1 and PC 2 are still strong but relatively weaker than the MALPACA-GS correlations (Fig 4B).

**Fig 5:**
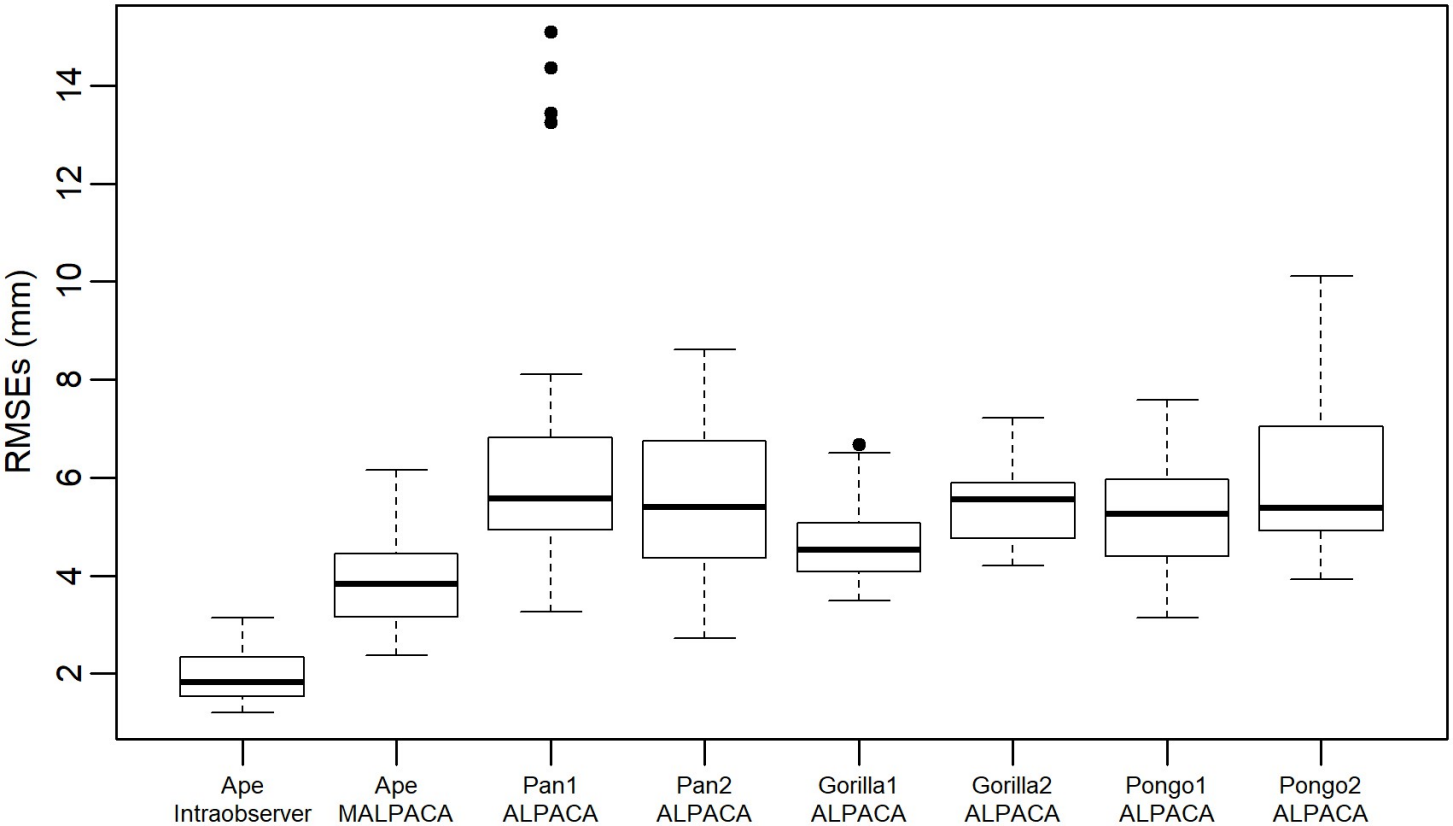
Comparison of RMSEs for the ape sample. RMSEs calculated between estimated landmarks and the GS landmarks. Each column represents the RMSEs of 46 ape specimens calculated by a specific analysis. “Ape Intraobserver” refers to the RMSEs between two manual landmark datasets of the ape sample. See S4 Fig for RMSEs as percentage of centroid sizes. See S2 Table for the template used for each ALPACA based on a K- means selected template.

**Fig 6:**
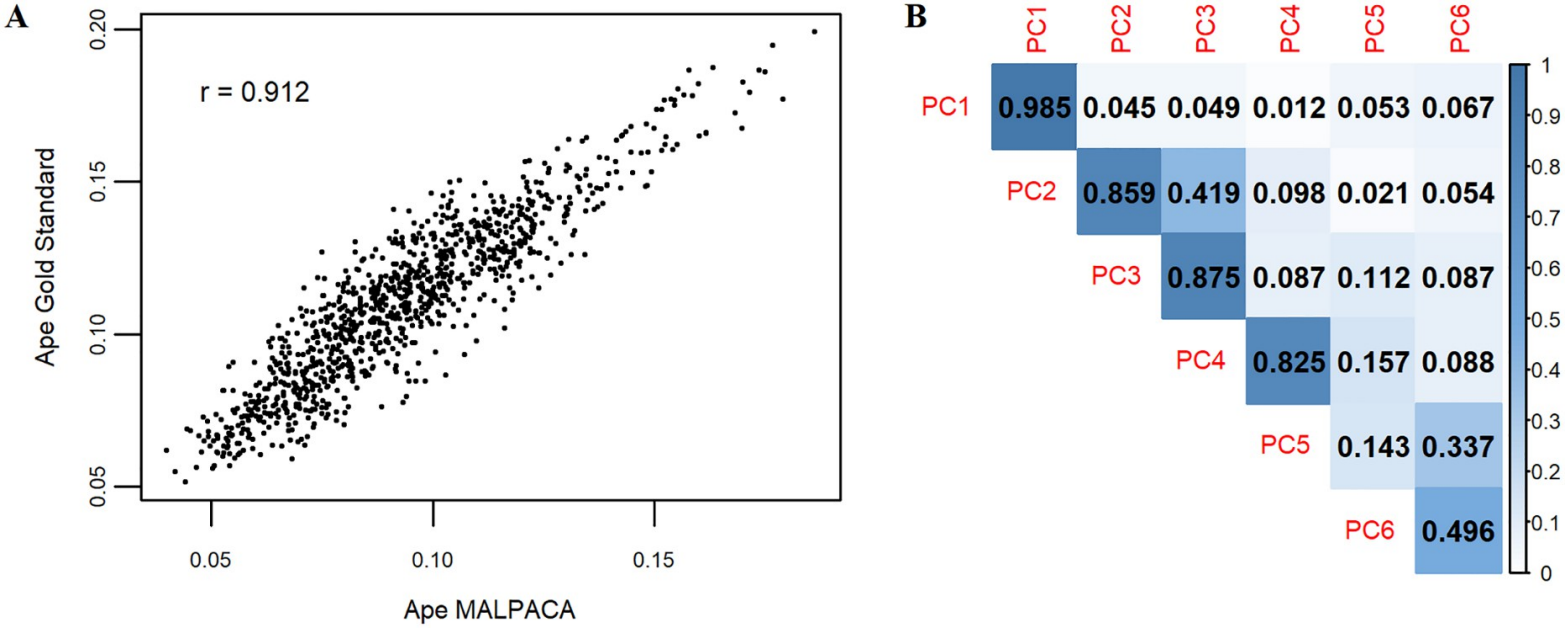
Ape MALPACA-Gold standard correlations in shape variables. (A) Estimated against GS pairwise Procrustes distances. (B) Correlations in the scores of the first six PCs (principal components) between estimated and GS landmarks. Each grid represents the correlation coefficient between a PC from an automated landmarking analysis and a PC from the GS, depending on its row and column names. Procrustes distances and PC scores are derived from separate GPAs of estimated and manual landmarks.

Overall, the ape MALPACA-ALPACA performance evaluations are in line with the results from the mouse dataset. MALPACA yields landmark estimates with significantly smaller RMSEs than any ALPACA estimate based on a K-means selected template (all p-values < 0.02) (Fig 5, S5 Fig and S8 Table). The mean shapes of MALPACA-based and GS landmarks derived from their joint GPA are highly consistent (S6 Fig). All centroid sizes from MALPACA and ALPACA show nearly perfect correlations with the GS as the correlation coefficients exceed 0.99, while the MALPACA-GS correlation is the highest (S9 Table). Both pairwise Procrustes distances and PC 1 scores computed from MALPACA-derived estimated landmarks very strongly correlates with those derived from GS (r = 0.912 and 0.985 respectively) (Fig 6A). The MALPACA-derived estimates and GS also yield strong correlations in PC 2, PC 3 and PC 4 scores as the correlation coefficients all exceed 0.8 (Fig 6B).

Overall, our study confirms the expectation that MALPACA outperforms ALPACA because multiple templates can better capture gross variations within a sample. In both the analyses of the mouse and ape samples, MALPACA outperforms each ALPACA run by estimating landmarks significantly closer to the GS (manual landmarks) and generating size and shape variables in morphometric analyses more similar to those derived from the GS.

Note that the ALPACA/MALPACA pipeline requires that the models are prepared consistently so that only the same anatomical structures are preserved across samples [10]. For example, if a few cranial models have floating polygons or extra postcranial components attached, the landmarking accuracy of these models can be negatively impacted [10]. Nevertheless, the ALPACA/MALPACA pipeline is robust with respect to small missing regions in specimens (e.g., a few broken teeth or small holes at the surface), and such specimens can still be used in the automated landmarking pipeline, provided that the landmark set does not contain any landmark that falls into the missing section (see the original ALPACA study [10] for detailed discussion about the quality of 3D models). When in doubt of whether small missing parts on a specimen or the quality of a 3D model will impact the ALPACA/MALPACA performance, users can use the ALPACA module in the SlicerMorph extension to conveniently test landmarking performance for this individual specimen [10, 20] (For tutorials, please see S1 Appendix and the ALPACA tutorial at https://github.com/SlicerMorph/Tutorials/tree/main/ALPACA).

### Manual landmark errors

**Fig 7:**
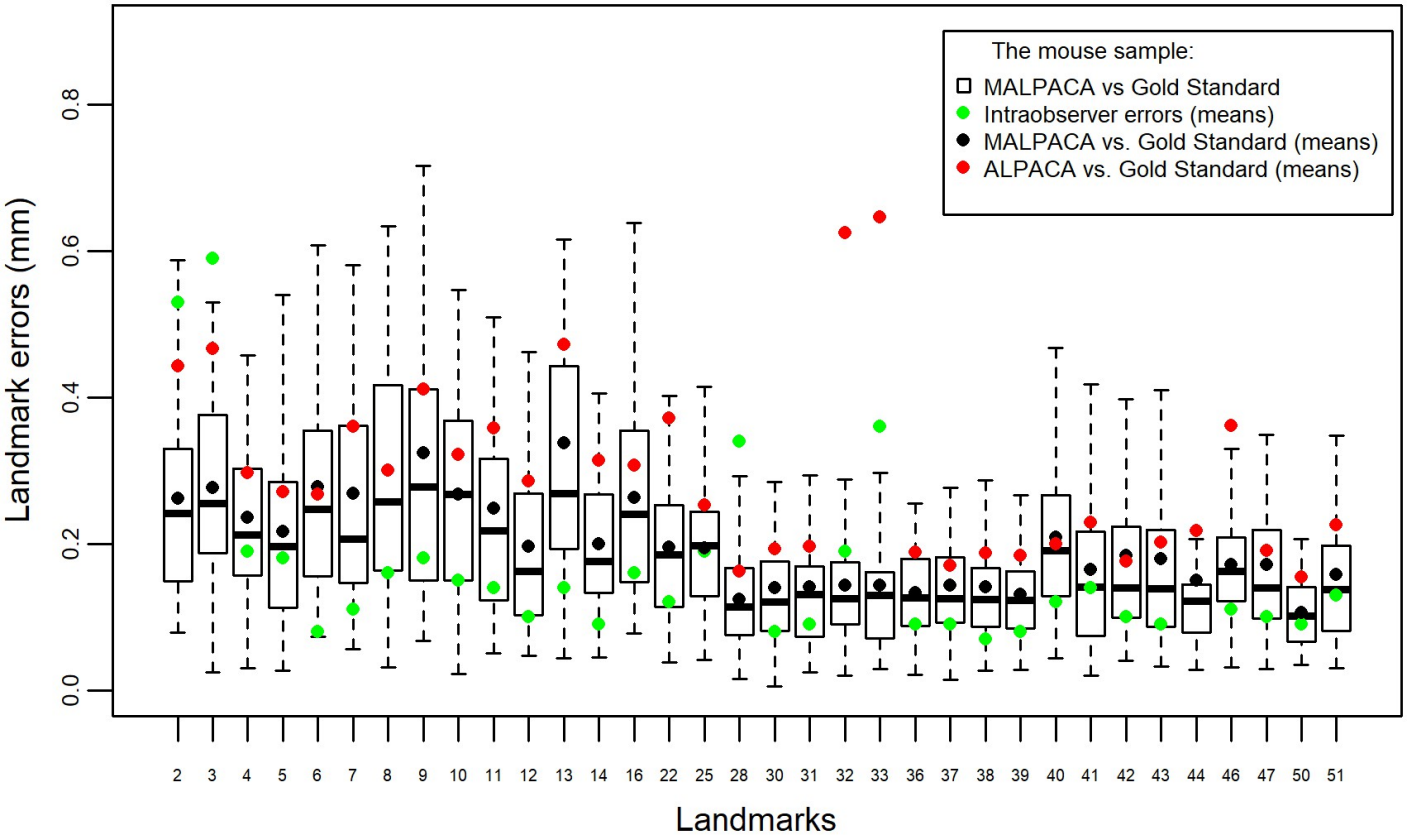
Individual landmark errors of the mouse sample comparing to intraobserver errors. Similar to Fig 2, columns represent errors between MALPACA and the GS for each landmark. Black dots: mean errors of the mouse MALPACA. Green dots: mean intraobserver errors computed by Percival et al. [9]. The labels on the x-axis are landmarks in this study that overlap with those from Percival et al. [9].

Fig 7 compares the mouse manual landmarking errors (intraobserver errors) calculated by Percival et al [9] to the errors between estimated and GS landmarks in our study for the 34 landmarks that are shared between these two studies. In general, intraobserver errors are the smallest with a mean error 0.163 mm. The ALPACA-GS errors are much larger as the mean error reaches 0.294 mm. The MALPACA-GS method outperforms ALPACA-GS with a mean error 0.2 mm and is more comparable to the manual landmarking error.

**Fig 8:**
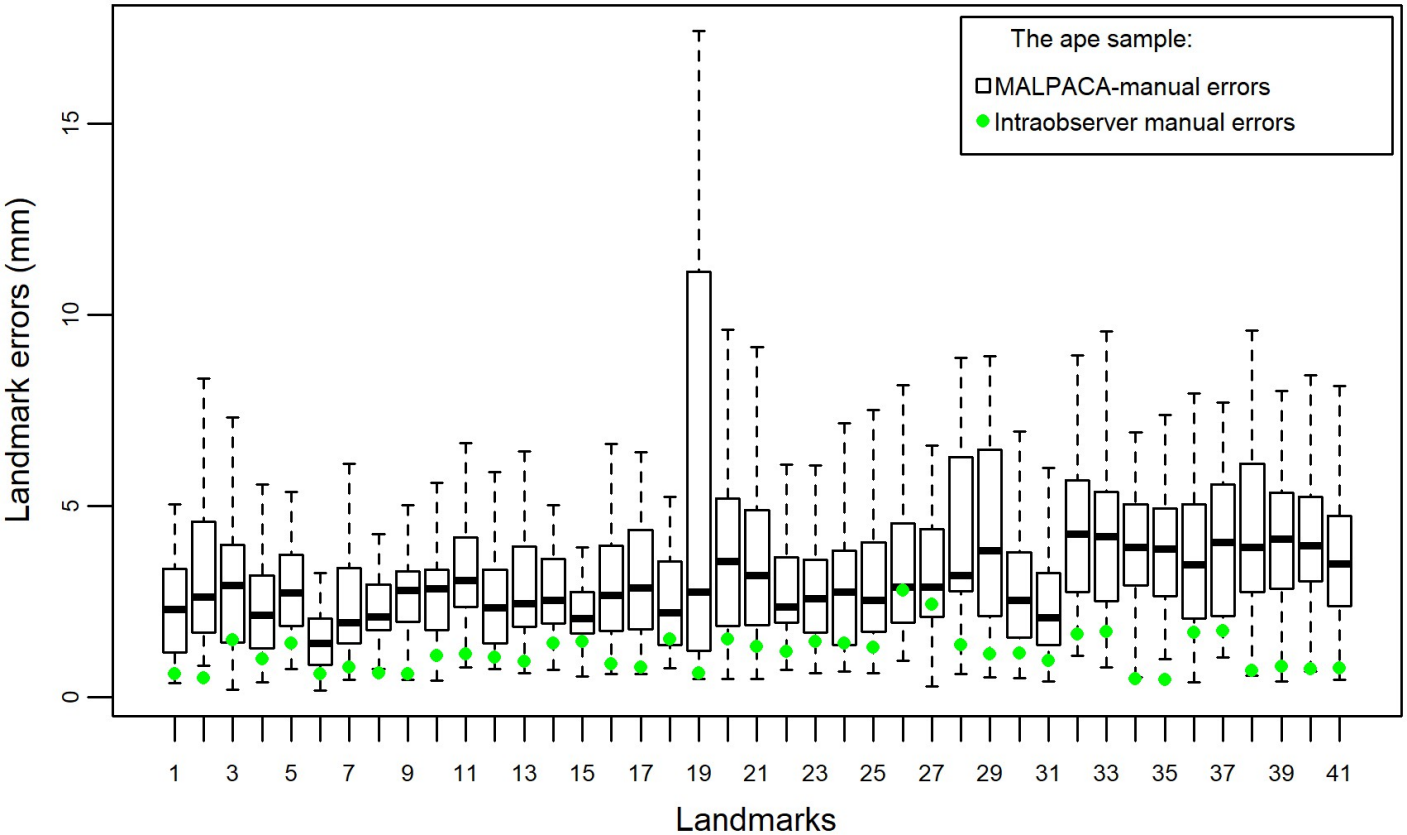
Individual landmark errors of the ape sample. Errors represented by Euclidean distances (for errors expressed in percentage of centroid sizes, see S3 Fig). Each column represents errors between MALPACA-derived estimates of all 46 ape specimens for the one landmark and their corresponding Gold Standard (GS) landmarks. Each green dot represents the median of the intraobserver manual errors of all 46 ape specimens for one landmark between two manual landmark sets.

For the ape sample, manual intraobserver errors are measured by the RMSEs, between two landmark sets. Again, the manual errors are significantly smaller than the errors between any set of estimates and GS landmarks (Fig 5, S5 Fig). The one-sided Welch t-test for assessing whether the manual errors are smaller than the MALPACA-manual errors yields a p-value of 2.381 × 10^−19^. Comparing individual landmark errors show that, for 38 of 41 landmarks, the intraobserver manual errors are significantly smaller than the MALPACA-manual errors (p-values < 0.05 based on the one-sided t-test that assesses if individual intraobserver landmark errors are smaller) (Fig 8, S7 Fig and S10 Table).

In both the mouse and ape samples, the deviations between MALPACA and GS are obviously much larger than the errors between two manual landmarking trials. This is as expected, as intraobserver errors created by the same expert are usually very small. Still, the MALPACA-GS errors in landmark positions are closer to the intraobserver errors than the ALPACA-GS errors.

Inter-observer errors are usually larger than intraobserver errors [13, 15, 16]. These errors can be as significant as some biologically meaningful variations, in particular those that are subtle but biologically meaningful, such as intraspecific variations and sexual dimorphism [9, 13, 15, 16]. This is especially the case when pooling discrete landmarks or a combination of discrete landmarks and sliding semilandmarks collected by different operators [16]. Furthermore, interobserver errors can be unpredictable as they have a variety of causes, including subjective understanding of anatomical variations, vague landmark protocols, and different landmark annotation tools [15, 16]. In recent years, pooling data collected by multiple researchers has become increasingly common [13, 15, 16]. Consequently, while research collaboration and data sharing are greatly increasing time efficiency for data collection and sample size for higher statistical power, controlling interobserver errors is becoming more difficult and complicated. This can create issues in accurately detecting subtle biological meaningful variations, consistency and reproducibility [13, 15, 16]. MALPACA, by contrasts, produces highly consistent landmarks when the same templates are used. Researchers can focus on carefully landmarking a short list of templates that can be shared with others. In this way, MALPACA can greatly facilitate data sharing while also ensuring consistency and reproducibility.

### The performance of K-means selected templates

Template selection for properly representing the whole sample is a critical factor in determining the performance of any template based method to avoid potential biases in the outcome especially when the sample is highly variable [26, 28, 29]. For example, in the field of automatic segmentation based on neuroimage registrations, various multi-atlas selection methods have been designed with differential accuracy for delineating organs [26, 28]. In this study, we propose a convenient method to identify multiple samples that can be used as templates for a study population by applying K-means clustering to the PCA scores derived from the downsampled point cloud data of their 3D models. It is expected that this approach will be able to capture gross patterns of overall shape variations when there is no prior information available to the investigator to guide the template selection.

**Fig 9:**
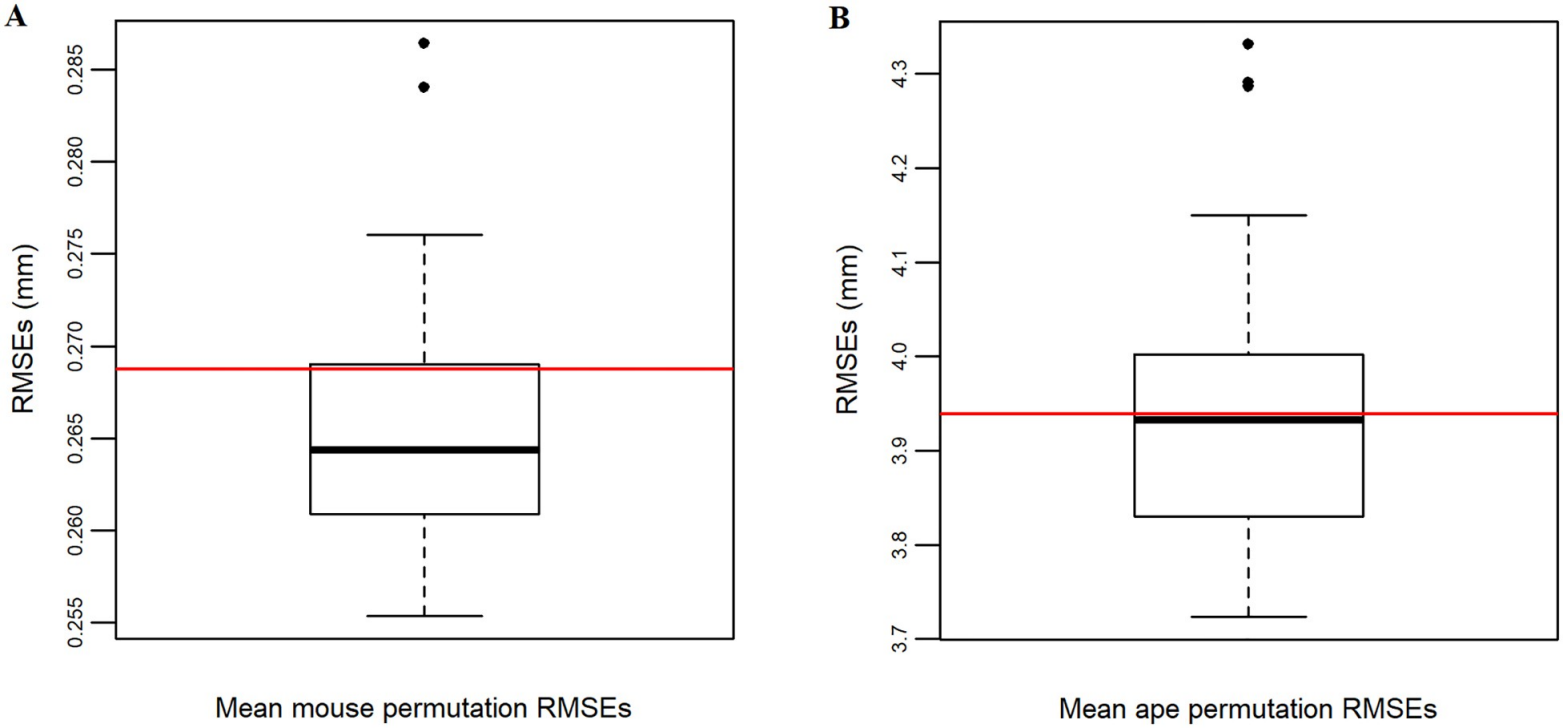
Permutation analyses for evaluating the performance of the K-means template selection method. (A) The boxplot shows the mean RMSEs of the 100 MALPACA runs from the mouse permutation analysis. The red horizontal line represents the mean RMSE from the mouse K-means based MALPACA. (B) The boxplot shows the mean RMSEs of the 50 MALPACA runs from the ape permutation analysis. The red horizontal line represents the mean RMSE from the ape K-means based MALPACA.

Permutation tests are carried out to assess whether K-means based templates outperform randomly selected templates as a way to determine the efficacy of K-means multi-template selection. For the mouse sample, the mean RMSE from the K-means based MALPACA is smaller than 28 of 100 (28%) mean RMSE scores from the permutated MALPACA runs (Fig 9A). This suggests that the K-means based MALPACA in general outperforms 28% of the MALPACA runs using randomly selected templates. Furthermore, 25 out of 54 specimens (46.2%) have RMSEs from the K-means based MALPACA smaller than the 50th percentile of the 5,400 RMSEs of individual specimens from the permutation analysis.

For the ape sample, the mean RMSE from the K-means based MALPACA is smaller than 24 of 50 (48%) mean RMSE scores derived from the permutated MALPACA runs (Fig 9B). This suggests that the K-means based MALPACA for the mouse sample in general outperforms the 48% of the MALPACA runs based on randomly selected templates. The comparison of individual RMSEs for the ape dataset is consistent with the analysis of the mouse sample. Overall, 20 out of 46 specimens (43.5%) have RMSEs from the K-means based MALPACA below the 50th percentile of the 4,600 RMSEs from the permutation analysis. In addition, the ranges of RMSEs derived from the mouse and ape K-means based MALPACA both overlap with the RMSEs derived from the permutation analysis (S11 Table).

These results show that running MALPACA with K-means selected templates neither outperforms nor underperforms the use of randomly selected templates. Furthermore, as shown in this study, the K-means based MALPACA consistently generates more accurate landmarks than does any single template-based analysis. Therefore, though the K-means template selection method may miss the template sets that are significantly better than the random template sets, it can effectively avoid selection of template sets that significantly underperform the randomly selected templates. As a result, the K-means approach to template selection provides increased consistency in the performance of MALPACA.

Investigators can skip the template selection procedure if they have other information that can be used to determine the templates to use (e.g., prior data, similar genetic background etc.). However, if there is uncertainty around what templates should be used, our K-means multi-template selection method provides a reasonable and safe solution for choosing specimens as templates for automated landmarking. Furthermore, the K-means template selection method is very efficient and convenient to run with our graphic user-interface (GUI) module, facilitating easy experimentation with this selection method when conducting MALPACA.

There are certain assumptions behind this approach that are important to consider before using K-means template selection. First, there is no rule for determining the number k for the K-means method. We use seven templates for the mouse sample and six templates for the ape sample for experiment purposes, which approximately reflect 10–12% of our sample sizes. More investigation needs to be done to identify what is the optimal number of templates (k) to use in a study to balance additional computational time versus improvement in estimates. However, we suspect this optimization will be highly dependent on the nature of the dataset being analyzed.

Second, K-means multi-template selection is determined by the sparse point clouds with point-to-point correspondence, which ultimately depends on the registration between each specimen and the reference. For convenience, this study selects the first one in the specimen list as the reference. If a different specimen is selected as the reference, the registration will be slightly different as will the point clouds. This may lead to a slightly different set of templates. Thus, it is important to assess how different choices of reference in point cloud generation may influence K-means multi-template selection, hence the final results of MALPACA in the future.

It is also important to note that the input models of the K-means template selection approach should contain the same corresponding structures across samples. For example, if 3D models of skull variably contain pieces of vertebrate, parts of mandibular joint, other non-cranial elements across samples, they will influence the analysis, because ultimately the analysis is dependent on the extracted point cloud. If a sample is partial or broken (e.g., missing a large section alveolar row on maxilla), it should be left out of the template selection analysis, because the correspondence of the point cloud generated from this sample is unlikely to match to that of others.

If users are uncertain about whether the quality of a 3D model is sufficient for the K-means template selection approach, they can use the GUI module to run the K-means pipeline on a small subset of the sample as a quick test run (see S1 Appendix for details). This GUI module will produce a PCA plot that shows the distribution of specimens according to the shape differences in their point cloud data. When an extreme outlier exists in the PCA plot, i.e., a specimen is widely separated from the rest of the sample, it may indicate that the quality of this specimen is too low so that its point cloud does not match well with the others. Because all the point clouds are exported, users can import the problematic one into Slicer to perform a visual check. On the other hand, as discussed previously, the ALPACA/MALPACA pipeline has a higher tolerance for small broken or missing parts on the models. Therefore, if a few models are imperfect for the K-means approach but are still proper for ALPACA/MALPACA (e.g., using the ALPACA module for a quick test as discussed previously), users can simply remove them from the K-means templates selection pipeline and add them back to the automated landmarking process after templates are selected.

### *Post-hoc* quality check and refined MALPACA performance

The MALPACA pipeline allows for conveniently designing *post-hoc* analyses to assess landmarking quality for further boosting performance because all estimates given by each template are exported. It is not easy for researchers to evaluate how well the automated landmarking performed without manually landmarking the target specimen and calculating the errors, or alternatively, visualizing the estimate on the target model. Both approaches are too tedious or downright unfeasible for a large study.

Consequently, we provide an example of how individual estimates can be conveniently used for a *post-hoc* evaluation of the quality of landmark estimates using simple heuristics in lieu of gold standard landmarks. The essence of the *post-hoc* quality check is to determine whether certain estimates generated from a template deviate too much from the corresponding estimates generated by other templates, thus can be treated as outliers.

**Fig 10:**
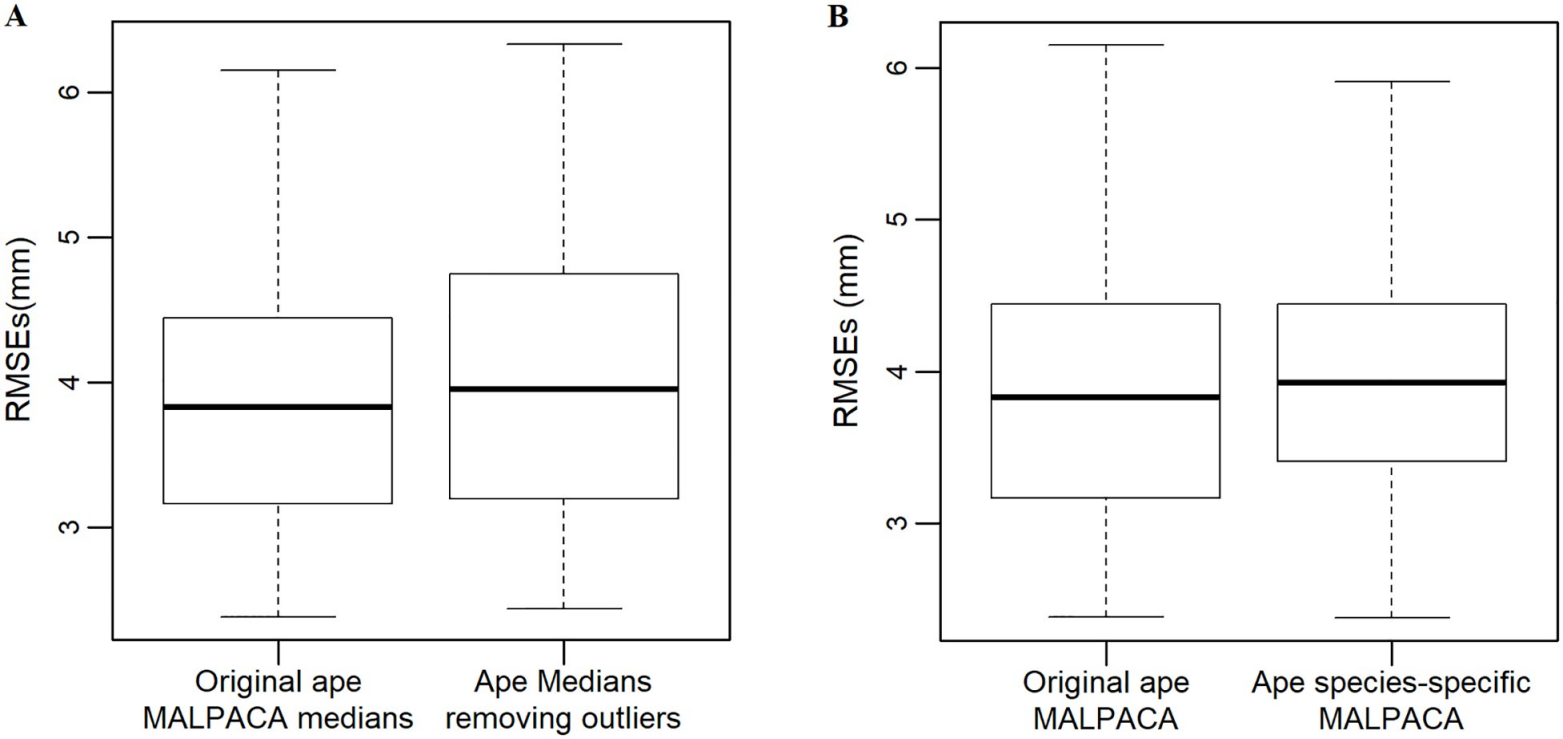
RMSEs comparison boxplots for ape refined MALPACAs. (A) The boxplot for comparing the performance of the ape MALPACA and the new median estimates achieved after removing outlier estimates demonstrated in S8 Fig. Comparison is based on calculating RMSEs between estimates and the GS. Two-sided Welch t-test shows that the RMSEs yielded from these two analyses are not significantly different with a p-value 0.5751. (B) RMSEs between the original ape MALPACA and “species-specific” MALPACA for the ape sample. Species-specific MALPACA refers to performing a MALPACA for one species only using templates of that species. RMSEs are calculated between estimates and the GS.

The outlier detection and removal functions successfully pinpoint the outlier estimates for the four *Gorilla* specimens with extreme RMSE values derived from the juvenile *Pan* template (USNM176236), along with other outliers given by other templates that exceed the threshold (Fig 5; S1 File). This is because differences between this juvenile *Pan* template and the adult *Gorilla* specimens are large hence the global registration in ALPACA poorly aligned their point clouds. This leads to erroneous landmarking results. After removing the identified outlier estimates, the RMSEs between the new medians and GS are not significantly smaller than the RMSEs derived from the original MALPACA median estimates and GS, suggesting similar performance (p-value = 0.5751; see Fig 10A). This is because the MALPACA landmark estimates are based on medians, which are more robust to outliers than means.

It should be noted that we choose to only remove the outlier estimates derived from this juvenile *Pan* template rather than removing all its estimates because the juvenile *Pan* template may still yield better estimates for certain landmarks on other specimens. If some estimates given by the juvenile *Pan* template deviate much from those given by the other templates, the outlier removal method can also automatically delete them. Users can opt to remove all estimates given by a template, like the *juvenile* Pan, if they find out that it yields poor estimates for many specimens. They can also define their own heuristic standards for outlier detection, such as increasing the heuristic standard to only pinpoint exceptionally worse estimates that can indicate poor alignment between a template and some target specimens.

Still, the issue derived from using a juvenile *Pan* template to landmark adult *Gorilla* specimens is indicative of situation when morphologically well-differentiated species exist within a study sample and including results from the templates of other species may have the potential to hamper the accuracy of the final estimates for specimens from each species. As a result, when templates from all distinct ape species are used for landmarking the whole sample, the final output of the MALPACA, i.e., the original MALPACA, may be sub-optimal. This motivates us to develop a “species-specific” approach of MALPACA. With the output estimates given by each individual template in hand, the species-specific MALPACA is easily done by calculating median values between the two landmark sets of each ape species derived from its two templates. The original and species-specific MALPACA estimates show largely consistent results, again demonstrating the advantage of using the median to buffer the impact of outlier estimates (Fig 10B and S8 Fig). On the other hand, the species-specific MALPACA-derived estimates do yield scores of PC 2 and PC 3 more correlated to those yielded by manual landmarks as the correlation coefficients both exceed 0.9, suggesting an improvement in morphometric analysis (S8B Fig).

Overall, the quality check of landmark estimates and refined MALPACA results show the flexibility and convenience of designing *post-hoc* analysis based on MALPACA output. We encourage users to try different approaches based on estimates of individual template and the structures of their samples. GUI modules for MALPACA and K-means multi-template selection are included in the existing open-access SlicerMorph module to facilitating the usage and exploration of these methods. For example, users can use the GUI module of MALPACA along with a *post-hoc* outlier detection analysis to conduct pilot studies using a subset of a large sample for efficiently evaluating the performance of K-means selected templates or experimenting with different template choices.

## Conclusion

We demonstrate that the multi-template automated landmarking method MALPACA outperforms ALPACA by estimating landmarks significantly closer to the “gold standard” manual landmarks. MALPACA is particularly suitable for landmarking morphologically diverse samples, such as the multi-taxa samples commonly encountered in evolutionary studies. The K-means template selection approach can yield a sufficiently good template set for MALPACA when users have no prior information for choosing templates, though this method is optional. Furthermore, we provide an example to show the flexibility of designing a *post-hoc* quality check for the performance of each template without a “gold standard” landmark set. Users are encouraged to use the accompanying GUI tools in SlicerMorph and the SlicerMorphR R package to explore different template choices and investigate their performance in MALPACA. Overall, MALPACA, as an open-source, lightweight, and user-friendly tool, offers the potential for large-scale collaboration and data sharing for morphometric analysis while ensuring accuracy, consistency, and reproducibility, thus helping to bring morphometrics fully into the “era of big data”.

## Supporting information

S1 Data Data and materials availability. The mouse data is freely available as part of https://doi.org/10.1111/joa.12645 and is also available at SlicerMorph/Mouse_Models (github.com). The raw DICOM sequences of hominoids used here are free for non-commercial use from the Smithsonian Institution’s National Museum of Natural History (NMNH) (http://humanorigins.si.edu/evidence/3d-collection/primate). Please contact the institution at https://dpo.si.edu to obtain access to the models. The ape manual landmark data are part of https://doi.org/10.1002/ajpa.24214. MALPACA derived LM estimated used in the ape study are available as part of the SlicerMorph/Mouse_Models (github.com) repository.
(DOCX)

S1 Fig Anatomical landmarks used in this study. (A) 51 landmarks for the mouse sample. (B) 41 landmarks for the ape sample. For data availability, see S1 Data.
(TIF)

S2 Fig Comparison of RMSEs (as percentage of centroid sizes) for the mouse sample. RMSEs calculated between estimated landmarks and the “gold standard”. Each box represents the RMSEs of 54 mouse specimens calculated by a specific analysis. “ALPACA”: Mouse ALPACA is estimated using the synthetic mouse template used in the original ALPACA paper. Other boxes are ALPACA-derived estimates using specified template. Centroid sizes are calculated from the Gold Standard landmark set.
(TIF)

S3 Fig Individual landmark errors (as percentage of centroid sizes) for the mouse sample. Errors represent by Euclidean distance transformed into percentage of centroid sizes. Each box represents errors between MALPACA-derived estimates of all 54 mouse specimens for one landmark and their corresponding Gold Standard (GS) landmarks. Red dots: each represents the median of errors between the estimates of the synthetic template ALPACA of all 54 mouse specimens for one landmark and their corresponding GS landmarks. Centroid sizes are calculated from the Gold Standard landmark set.
(TIF)

S4 Fig Superimposition of joint GPA for mouse MALPACA and manual landmarks (XY dimensions) based on the mouse sample. Light blue dots: all manual landmarks. Dark blue cross: mean manual landmarks. Light red dots: all MALPACA-derived estimated landmarks. Deep red cross: mean MALPACA-derived estimated landmarks.
(TIF)

S5 Fig Comparison of RMSEs (as percentage of centroid sizes) for the ape sample. RMSEs calculated between estimated landmarks and the “gold standard”. Each box represents the RMSEs of 46 ape specimens calculated by a specific analysis. “Ape Intraobserver” refers to the RMSEs between two manual landmark datasets of the ape sample. See S4 Fig for RMSEs as percentage of centroid sizes. See Table 2 for the template used for each ALPACA based on a K- means selected template. Centroid sizes are calculated from the Gold Standard landmark set.
(TIF)

S6 Fig Superimposition of joint GPA for ape MALPACA and manual landmarks (XY dimensions) based on the ape sample. Light blue dots: all manual landmarks. Dark blue cross: mean manual landmarks. Light red dots: all MALPACA-derived estimated landmarks. Deep red cross: mean MALPACA-derived estimated landmarks.
(TIF)

S7 Fig Individual landmark errors of the ape sample as in percentage of centroid sizes. Boxes represent errors between MALPACA-derived estimates and the Gold Standard (GS) landmarks. Green dots represent median intraobserver manual landmark errors between two manual landmark sets. Centroid sizes are calculated from the Gold Standard landmark set.
(TIF)

S8 Fig Performance of ape species-specific MALPACA comparing to manual landmarks. (A) Correlation in the pairwise Procrustes distances derived from species-specific MALPACA and manual landmarks. (B) Correlations in PC scores derived from species-specific MALPACA and manual landmarks. Each grid represents the correlation coefficient between a PC from an automated landmarking analysis and a PC from the Gold Standard, depending on its row and column names.
(TIF)

S1 Table Specimen ID for the mouse sample. Sample size = 61. The K-means selected templates are bold and marked by “template”.
(DOCX)

S2 Table Specimen IDs and species designation for the ape sample. Sample size = 52. The two templates selected by K-means per species (six templates total) are bold and marked by “template” in the parenthesis. The remaining 46 specimens are being landmarked.
(DOCX)

S3 Table Settings for K-means multi-template selection. MALPACA uses the default settings listed in the ALPACA module of the SlicerMorph extension. See Porto et al. [10] for detail. Spacing factor determines the point cloud density for K-means. See section 2 of the supplementary material for details. In general, the settings ensure that each point cloud used for the K-means template selection has 700 to 800 points. Also see S1 Appendix for explanations of these settings.
(DOCX)

S4 Table P-values from one sided Welch t-tests comparing mouse MALPACA and ALPACA RMSEs. One-sided t-tests compare whether mouse MALPACA RMSEs significantly smaller than ALPACA RMSEs using the synthetic template and individual mouse template.
(DOCX)

S5 Table P-values of one-sided Welch t-tests that compare if MALPACA individual landmark errors are smaller than ALPACA errors. MALPACA errors versus manual landmarks significantly smaller than ALPACA errors in the 37 landmarks listed.
(DOCX)

S6 Table P-values of one-sided Welch t-tests that compare if ALPACA individual landmark errors are smaller than MALPACA errors for the mouse sample. Only listing landmarks not listed in S5 Table, i.e., MALPACA-derived estimates do not show significantly smaller errors than ALPACA ones in these 14 landmarks according to the one-sided t-test in S5 Table.
(DOCX)

S7 Table Correlations between estimated and manual landmarks in centroid sizes for the mouse sample. (DOCX)

S8 Table P-values from one sided Welch t-tests comparing ape MALPACA and ALPACA RMSEs derived from individual template. One-sided t-tests compare whether ape MALPACA RMSEs significantly smaller than ALPACA RMSEs using an individual ape template.
(DOCX)

S9 Table Correlations between estimated and manual landmarks in centroid sizes for the ape sample. (DOCX)

S10 Table Correlations between estimated and manual landmarks in centroid sizes for the ape sample. (DOCX)

S11 Table RMSEs of K-means-based MALPACAs comparing to the permutated RMSEs. The values in the paratheses are corresponding quantile values of permutation analyses.
(DOCX)

S1 File Boxplots for the ape individual estimates and the threshold for choosing outliers. Each graph shows the boxplot of distances between individual estimates of one ape specimen to the corresponding MALPACA final output. The red horizontal line represents the threshold, which is two standard deviations above the mean of the distances for that specimen.
(PDF)

S1 Appendix Step-by-step instructions for running K-means template selection and MALPACA. (DOCX)

## References

[1] Adams DC, Rohlf FJ, Slice DE. A field comes of age: geometric morphometrics in the 21st century. Hystrix. 2013;24: 7–14. doi: 10.4404/hystrix-24.1–6283

[2] Baab KL, McNulty KP, Rohlf FJ. The shape of human evolution: a geometric morphometrics perspective. Evol Anthropol Issues News Rev. 2012;21: 151–165. doi: 10.1002/evan.21320 22907868

[3] Latif A, Kuijpers MA, Rachwalski M, Latief BS, Kuijpers-Jagtman AM, Fudalej PS. Morphological variability in unrepaired bilateral clefts with and without cleft palate evaluated with geometric morphometrics. J Anat. 2020;236: 425–433. doi: 10.1111/joa.13118 31792971 PMC7018634

[4] Motch Perrine SM, Cole TM, Martínez-Abadías N, Aldridge K, Jabs EW, Richtsmeier JT. Craniofacial divergence by distinct prenatal growth patterns in Fgfr2 mutant mice. BMC Dev Biol. 2014;14: 1–17. doi: 10.1186/1471-213X-14-8 24580805 PMC4101838

[5] Motch Perrine SM, Wu M, Stephens NB, Kriti D, Van Bakel H, Jabs EW, et al. Mandibular dysmorphology due to abnormal embryonic osteogenesis in FGFR2-related craniosynostosis mice. Dis Model Mech. 2019;12: dmm038513. doi: 10.1242/dmm.038513 31064775 PMC6550049

[6] Muñoz MM, Price SA. The future is bright for evolutionary morphology and biomechanics in the era of big data. Integr Comp Biol. 2019;59: 599–603. doi: 10.1093/icb/icz121 31353403

[7] Rutland JW, Bellaire CP, Yao A, Arrighi-Allisan A, Napoli JG, Delman BN, et al. The Expanding Role of Geometric Morphometrics in Craniofacial Surgery. J Craniofac Surg. 2021;32: 1104–1109. doi: 10.1097/SCS.0000000000007362 34779599

[8] Aneja D, Vora SR, Camci ED, Shapiro LG, Cox TC. Automated detection of 3d landmarks for the elimination of non-biological variation in geometric morphometric analyses. 2015 IEEE 28th International Symposium on Computer-Based Medical Systems. IEEE; 2015. pp. 78–83. 10.1109/CBMS.2015.86 PMC4526271 26258171

[9] Percival CJ, Devine J, Darwin BC, Liu W, van Eede M, Henkelman RM, et al. The effect of automated landmark identification on morphometric analyses. J Anat. 2019;234: 917–935. doi: 10.1111/joa.12973 30901082 PMC6539672

[10] Porto A, Rolfe S, Maga AM. ALPACA: A fast and accurate computer vision approach for automated landmarking of three-dimensional biological structures. Methods Ecol Evol. 2021;12: 2129–2144. doi: 10.1111/2041-210X.13689 35874971 PMC9291522

[11] Pui S, Minoi J-L. A Non-template Based Automatic Landmarking on 3D Face Data. Proceedings of the 3rd International Conference on Video and Image Processing. 2019. pp. 212–216.

[12] Young R, Maga AM. Performance of single and multi-atlas based automated landmarking methods compared to expert annotations in volumetric microCT datasets of mouse mandibles. Front Zool. 2015;12: 1–12. doi: 10.1186/s12983-015-0127-8 26628903 PMC4666065

[13] Daboul A, Ivanovska T, Bülow R, Biffar R, Cardini A. Procrustes-based geometric morphometrics on MRI images: An example of inter-operator bias in 3D landmarks and its impact on big datasets. PloS One. 2018;13: e0197675. doi: 10.1371/journal.pone.0197675 29787586 PMC5963746

[14] Percival CJ, Green R, Marcucio R, Hallgrímsson B. Surface landmark quantification of embryonic mouse craniofacial morphogenesis. BMC Dev Biol. 2014;14: 1–12. doi: 10.1186/1471-213X-14-31 25059626 PMC4222779

[15] Robinson C, Terhune CE. Error in geometric morphometric data collection: Combining data from multiple sources. Am J Phys Anthropol. 2017;164: 62–75. doi: 10.1002/ajpa.23257 28573732

[16] Evin A, Bonhomme V, Claude J. Optimizing digitalization effort in morphometrics. Biol Methods Protoc. 2020;5: bpaa023. doi: 10.1093/biomethods/bpaa023 33324759 PMC7723759

[17] Fruciano C. Measurement error in geometric morphometrics. Dev Genes Evol. 2016;226: 139–158. doi: 10.1007/s00427-016-0537-4 27038025

[18] Bromiley PA, Schunke AC, Ragheb H, Thacker NA, Tautz D. Semi-automatic landmark point annotation for geometric morphometrics. Front Zool. 2014;11: 1–21. doi: 10.1186/s12983-014-0061-124401080

[19] Devine J, Aponte JD, Katz DC, Liu W, Vercio LDL, Forkert ND, et al. A registration and deep learning approach to automated landmark detection for geometric morphometrics. Evol Biol. 2020;47: 246–259. doi: 10.1007/s11692-020-09508-8 33583965 PMC7880197

[20] Maga AM, Tustison NJ, Avants BB. A population level atlas of Mus musculus craniofacial skeleton and automated image-based shape analysis. J Anat. 2017;231: 433–443. doi: 10.1111/joa.12645 28656622 PMC5554826

[21] Rolfe S, Pieper S, Porto A, Diamond K, Winchester J, Shan S, et al. SlicerMorph: An open and extensible platform to retrieve, visualize and analyze 3D morphology. Methods Ecol Evol. 2021;12: 1816–1825. doi: 10.1111/2041-210X.13669 PMC12094517 40401087

[22] Kikinis R, Pieper SD, Vosburgh KG. 3D Slicer: a platform for subject-specific image analysis, visualization, and clinical support. Intraoperative imaging and image-guided therapy. Springer; 2014. pp. 277–289. doi: 10.1007/978-1-4614-7657-3_19

[23] Doshi J, Erus G, Ou Y, Resnick SM, Gur RC, Gur RE, et al. MUSE: Multi-atlas region Segmentation utilizing Ensembles of registration algorithms and parameters, and locally optimal atlas selection. Neuroimage. 2016;127: 186–195. doi: 10.1016/j.neuroimage.2015.11.073 26679328 PMC4806537

[24] Iglesias JE, Sabuncu MR. Multi-atlas segmentation of biomedical images: a survey. Med Image Anal. 2015;24: 205–219. doi: 10.1016/j.media.2015.06.012 26201875 PMC4532640

[25] Rohlfing T, Brandt R, Menzel R, Russakoff DB, Maurer CR. Quo vadis, atlas-based segmentation? Handbook of biomedical image analysis. Springer; 2005. pp. 435–486. doi: 10.1007/0-306-48608-3_11

[26] Schipaanboord B, Boukerroui D, Peressutti D, van Soest J, Lustberg T, Dekker A, et al. An evaluation of atlas selection methods for atlas-based automatic segmentation in radiotherapy treatment planning. IEEE Trans Med Imaging. 2019;38: 2654–2664. doi: 10.1109/TMI.2019.2907072 30969918

[27] Wang H, Yushkevich P. Multi-atlas segmentation with joint label fusion and corrective learning—an open source implementation. Front Neuroinformatics. 2013;7: 27. doi: 10.3389/fninf.2013.00027 24319427 PMC3837555

[28] Antonelli M, Cardoso MJ, Johnston EW, Appayya MB, Presles B, Modat M, et al. GAS: A genetic atlas selection strategy in multi-atlas segmentation framework. Med Image Anal. 2019;52: 97–108. doi: 10.1016/j.media.2018.11.007 30476698

[29] Gooding MJ. Evaluation of Atlas Selection: How Close Are We to Optimal Selection? Auto-Segmentation for Radiation Oncology. CRC Press; 2021. pp. 19–38.

[30] Rolfe S, Davis C, Maga AM. Comparing semi-landmarking approaches for analyzing three-dimensional cranial morphology. Am J Phys Anthropol. 2021;175: 227–237. doi: 10.1002/ajpa.24214 33483951 PMC12088985

[31] Rusinkiewicz S, Levoy M. Efficient variants of the ICP algorithm. Proceedings third international conference on 3-D digital imaging and modeling. IEEE; 2001. pp. 145–152.

[32] Rusu RB, Blodow N, Beetz M. Fast point feature histograms (FPFH) for 3D registration. 2009 IEEE international conference on robotics and automation. IEEE; 2009. pp. 3212–3217.

[33] Zhou Q-Y, Park J, Koltun V. Open3D: A modern library for 3D data processing. ArXiv Prepr ArXiv180109847. 2018.

[34] Schroeder W, Martin KM, Lorensen WE. The visualization toolkit: an object-oriented approach to 3D graphics. 4th ed. Prentice-Hall, Inc.; 2006.

[35] Zelditch ML, Swiderski DL, Sheets HD. Geometric morphometrics for biologists: a primer. academic press; 2012.

[36] Virtanen P, Gommers R, Oliphant TE, Haberland M, Reddy T, Cournapeau D, et al. SciPy 1.0: fundamental algorithms for scientific computing in Python. Nat Methods. 2020;17: 261–272. doi: 10.1038/s41592-019-0686-2 32015543 PMC7056644

[37] R Core Team. R: A language and environment for statistical computing. Vienna, Austria: R Foundation for Statistical Computing; 2021. https://www.R-project.org/.

[38] Adams DC, Collyer M, Kaliontzopoulou A, Baken EK. Geomorph: Software for geometric morphometric analyses. R package version 4.0. 2021. https://cran.r-project.org/package=geomorph.

