# Supplementary material for: Automated landmarking via multiple templates
Source: PLoS One. 2022 Dec 1;17(12):e0278035. doi: 10.1371/journal.pone.0278035 (PMC9714854; doi:10.1371/journal.pone.0278035)
Supplement: S1 Data — The mouse data is freely available as part of https://doi.org/10.1111/joa.12645 and is also available at SlicerMorph/Mouse_Models (github.com). The raw DICOM sequences of hominoids used here are free for non-commercial use from the Smithsonian Institution’s National Museum of Natural History (NMNH) (http://humanorigins.si.edu/evidence/3d-collection/primate). Please contact the institution at https://dpo.si.edu to obtain access to the models. The ape manual landmark data are part of https://doi.org/10.1002/ajpa.24214. MALPACA derived LM estimated used in the ape study are available as part of the SlicerMorph/Mouse_Models (github.com) repository. (DOCX) [file pone.0278035.s001.docx]

The mouse data is freely available as part of <https://doi.org/10.1111/joa.12645> and is also available at [SlicerMorph/Mouse_Models (github.com)](https://github.com/SlicerMorph/mouse_models). The raw DICOM sequences of hominoids used here are free for non-commercial use from the Smithsonian Institution's National Museum of Natural History (NMNH) (<http://humanorigins.si.edu/evidence/3d-collection/primate>). Please contact the institution at [https://dpo.si.edu](https://dpo.si.edu/) to obtain access to the models. The ape manual landmark data are part of <https://doi.org/10.1002/ajpa.24214>. MALPACA derived LM estimated used in the ape study are available as part of the [SlicerMorph/Mouse_Models (github.com)](https://github.com/SlicerMorph/mouse_models) repository.
