# Supplementary figures and images for: Automated landmarking via multiple templates
Source: PLoS One. 2022 Dec 1;17(12):e0278035. doi: 10.1371/journal.pone.0278035 (PMC9714854; doi:10.1371/journal.pone.0278035)

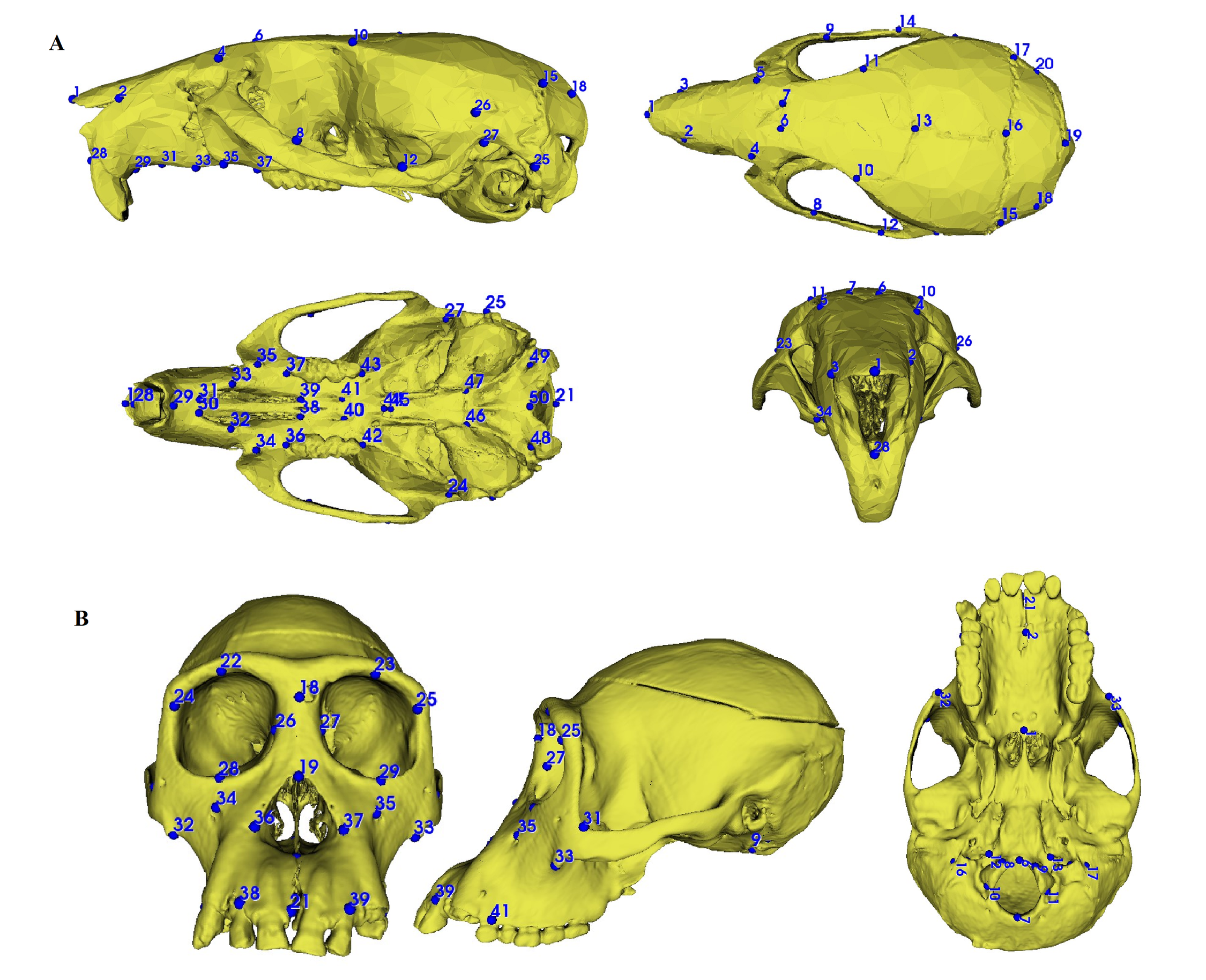

Supplement: S1 Fig — (A) 51 landmarks for the mouse sample. (B) 41 landmarks for the ape sample. For data availability, see S1 Data. (TIF) [file pone.0278035.s002.tif]

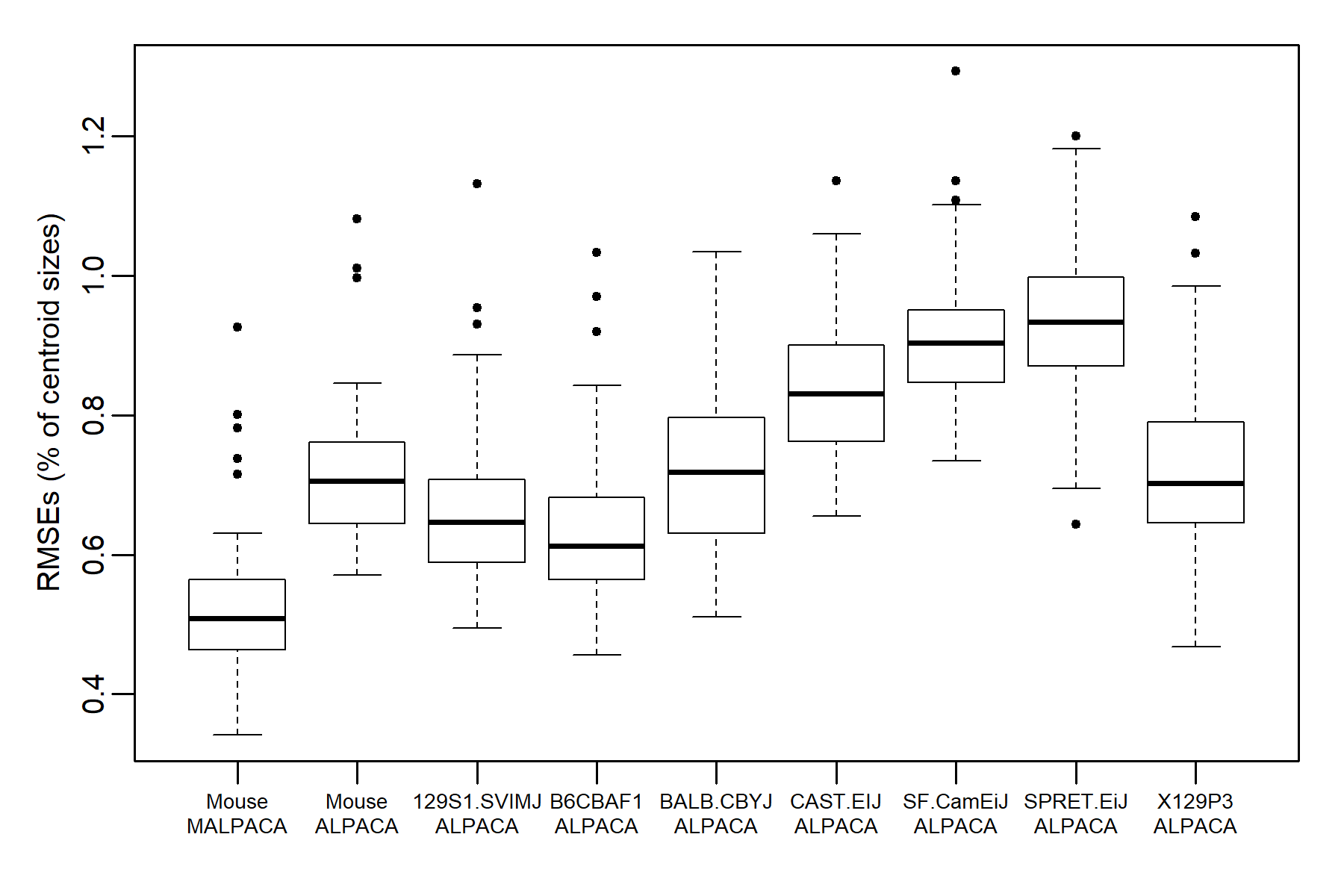

Supplement: S2 Fig — RMSEs calculated between estimated landmarks and the “gold standard”. Each box represents the RMSEs of 54 mouse specimens calculated by a specific analysis. “ALPACA”: Mouse ALPACA is estimated using the synthetic mouse template used in the original ALPACA paper. Other boxes are ALPACA-derived estimates using specified template. Centroid sizes are calculated from the Gold Standard landmark set. (TIF) [file pone.0278035.s003.tif]

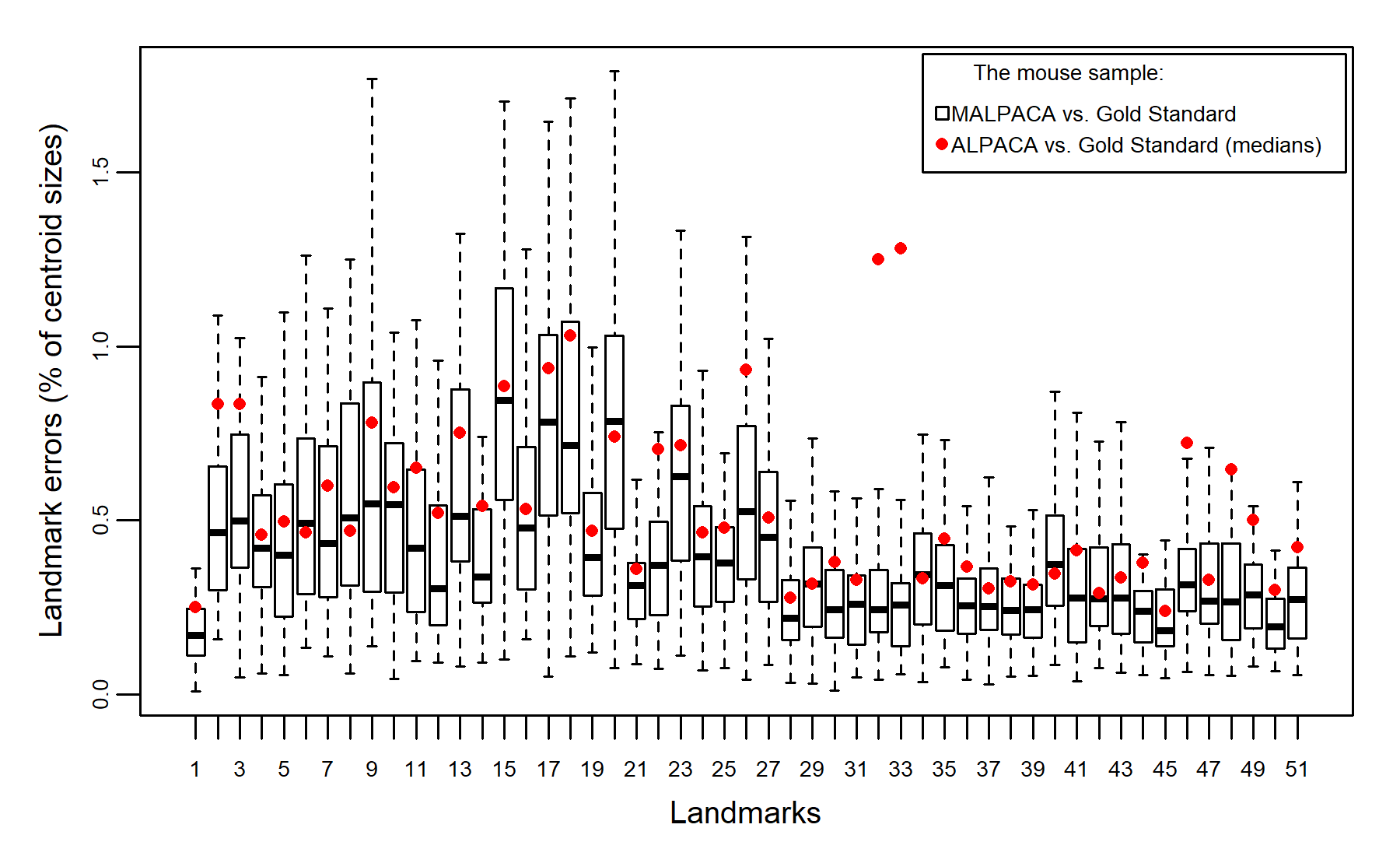

Supplement: S3 Fig — Errors represent by Euclidean distance transformed into percentage of centroid sizes. Each box represents errors between MALPACA-derived estimates of all 54 mouse specimens for one landmark and their corresponding Gold Standard (GS) landmarks. Red dots: each represents the median of errors between the estimates of the synthetic template ALPACA of all 54 mouse specimens for one landmark and their corresponding GS landmarks. Centroid sizes are calculated from the Gold Standard landmark set. (TIF) [file pone.0278035.s004.tif]

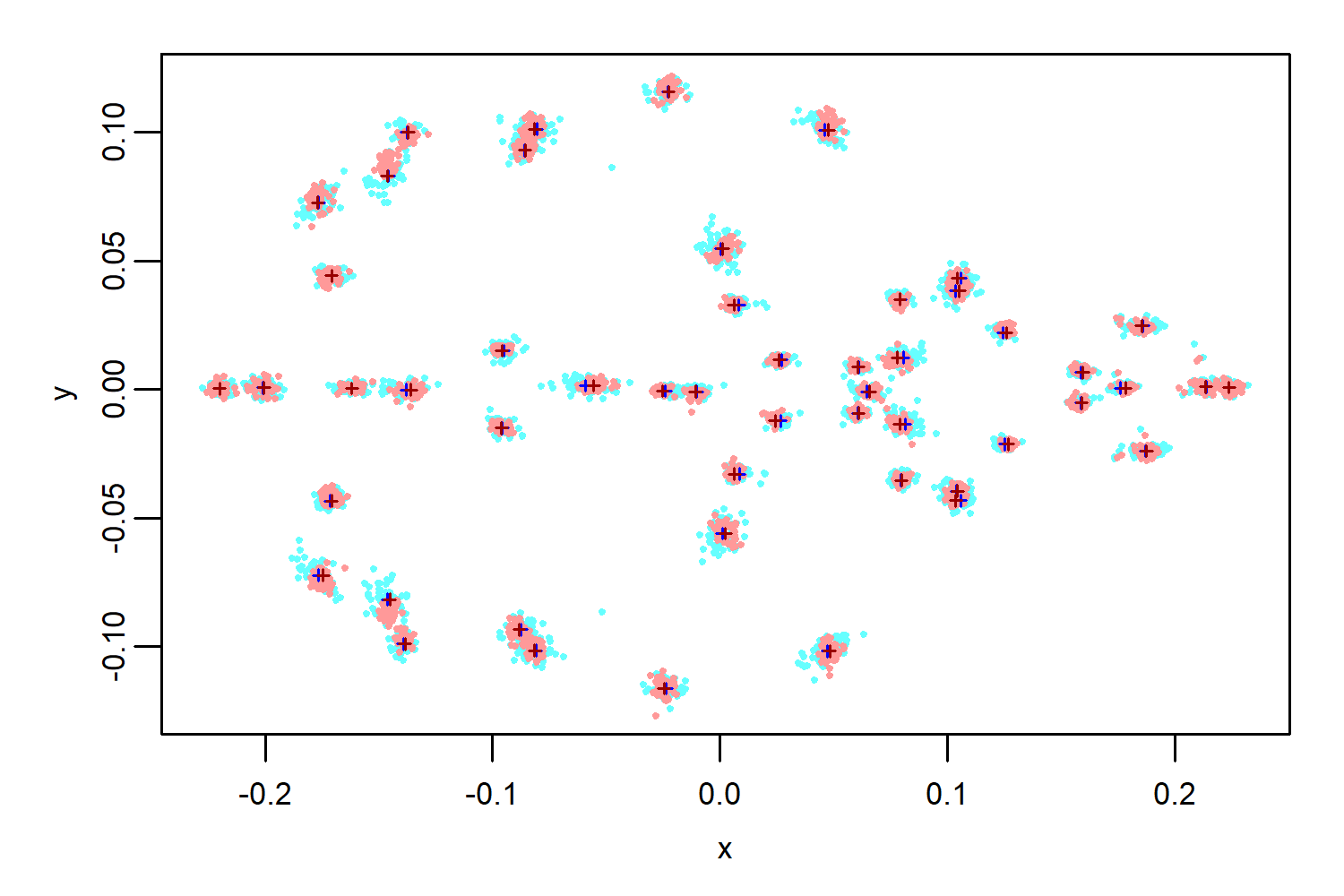

Supplement: S4 Fig — Light blue dots: all manual landmarks. Dark blue cross: mean manual landmarks. Light red dots: all MALPACA-derived estimated landmarks. Deep red cross: mean MALPACA-derived estimated landmarks. (TIF) [file pone.0278035.s005.tif]

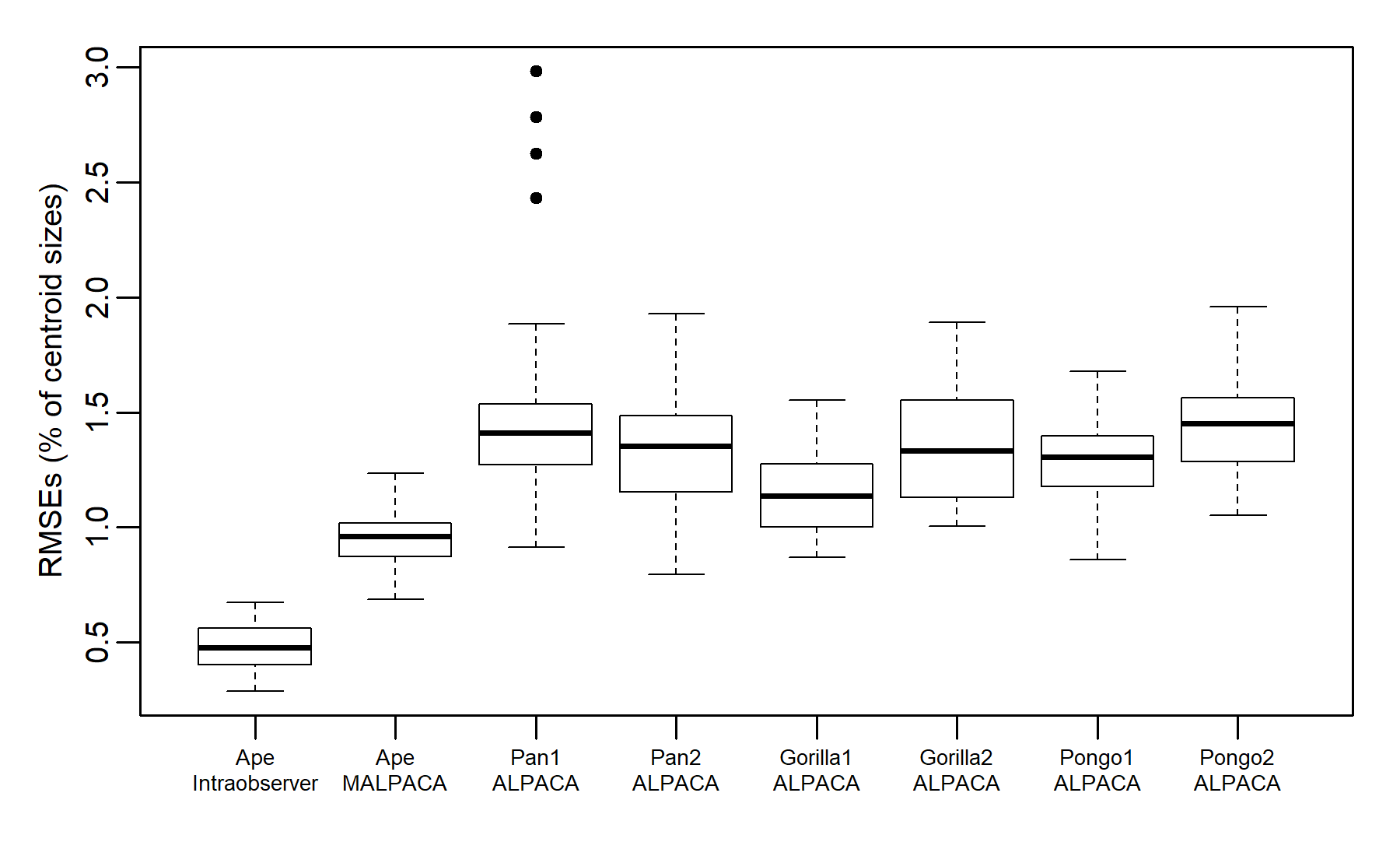

Supplement: S5 Fig — RMSEs calculated between estimated landmarks and the “gold standard”. Each box represents the RMSEs of 46 ape specimens calculated by a specific analysis. “Ape Intraobserver” refers to the RMSEs between two manual landmark datasets of the ape sample. See S4 Fig for RMSEs as percentage of centroid sizes. See Table 2 for the template used for each ALPACA based on a K- means selected template. Centroid sizes are calculated from the Gold Standard landmark set. (TIF) [file pone.0278035.s006.tif]

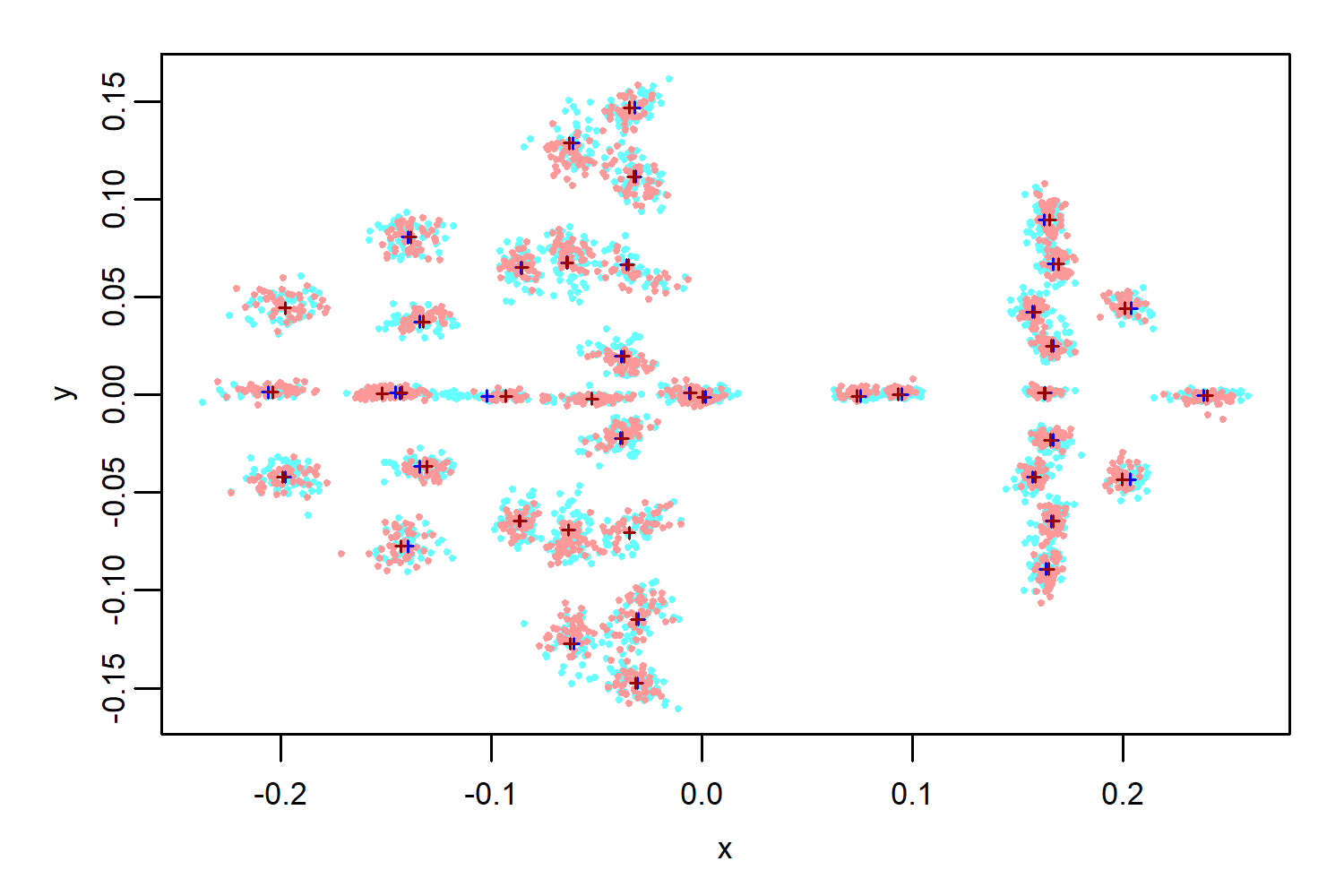

Supplement: S6 Fig — Light blue dots: all manual landmarks. Dark blue cross: mean manual landmarks. Light red dots: all MALPACA-derived estimated landmarks. Deep red cross: mean MALPACA-derived estimated landmarks. (TIF) [file pone.0278035.s007.tif]

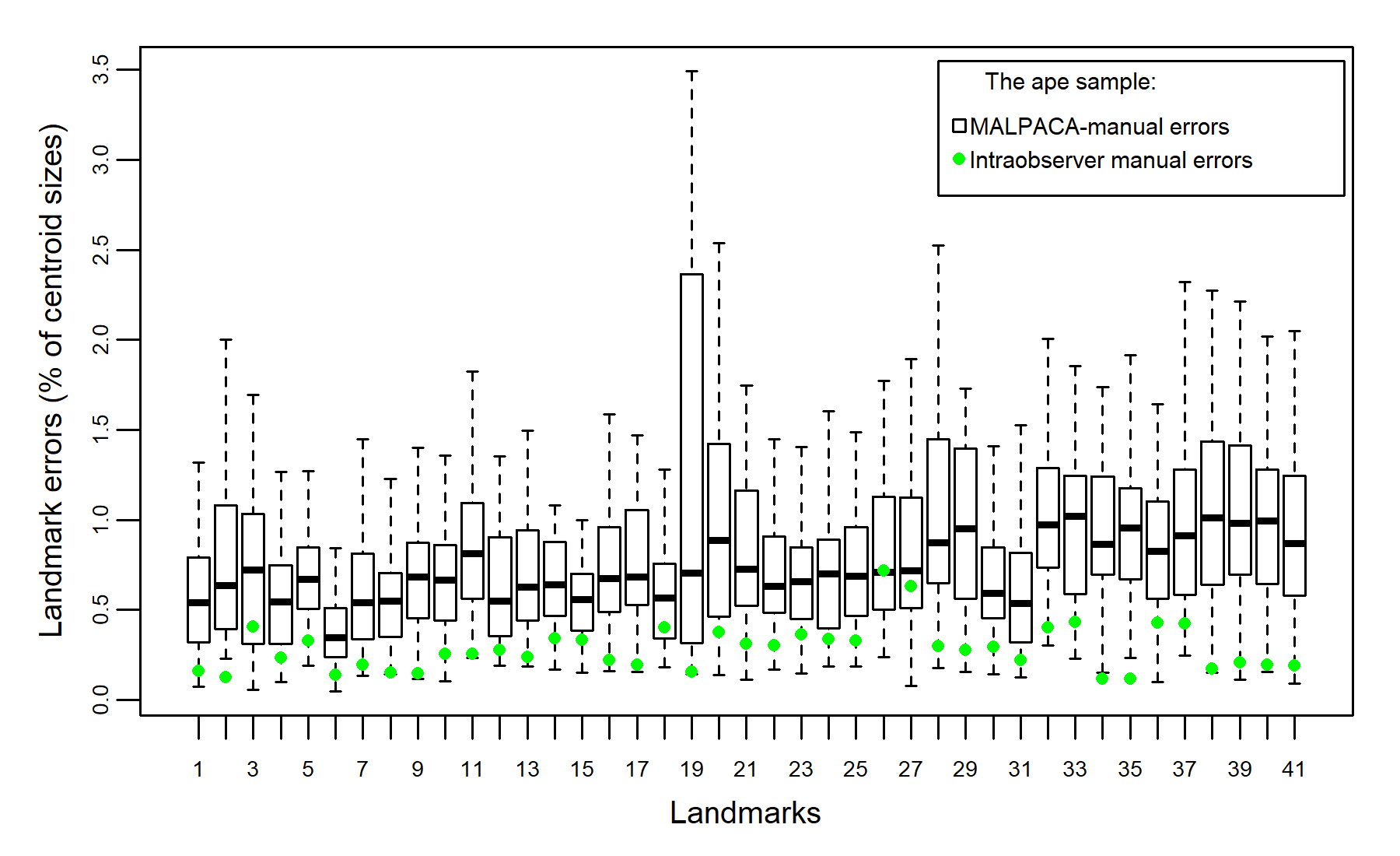

Supplement: S7 Fig — Boxes represent errors between MALPACA-derived estimates and the Gold Standard (GS) landmarks. Green dots represent median intraobserver manual landmark errors between two manual landmark sets. Centroid sizes are calculated from the Gold Standard landmark set. (TIF) [file pone.0278035.s008.tif]

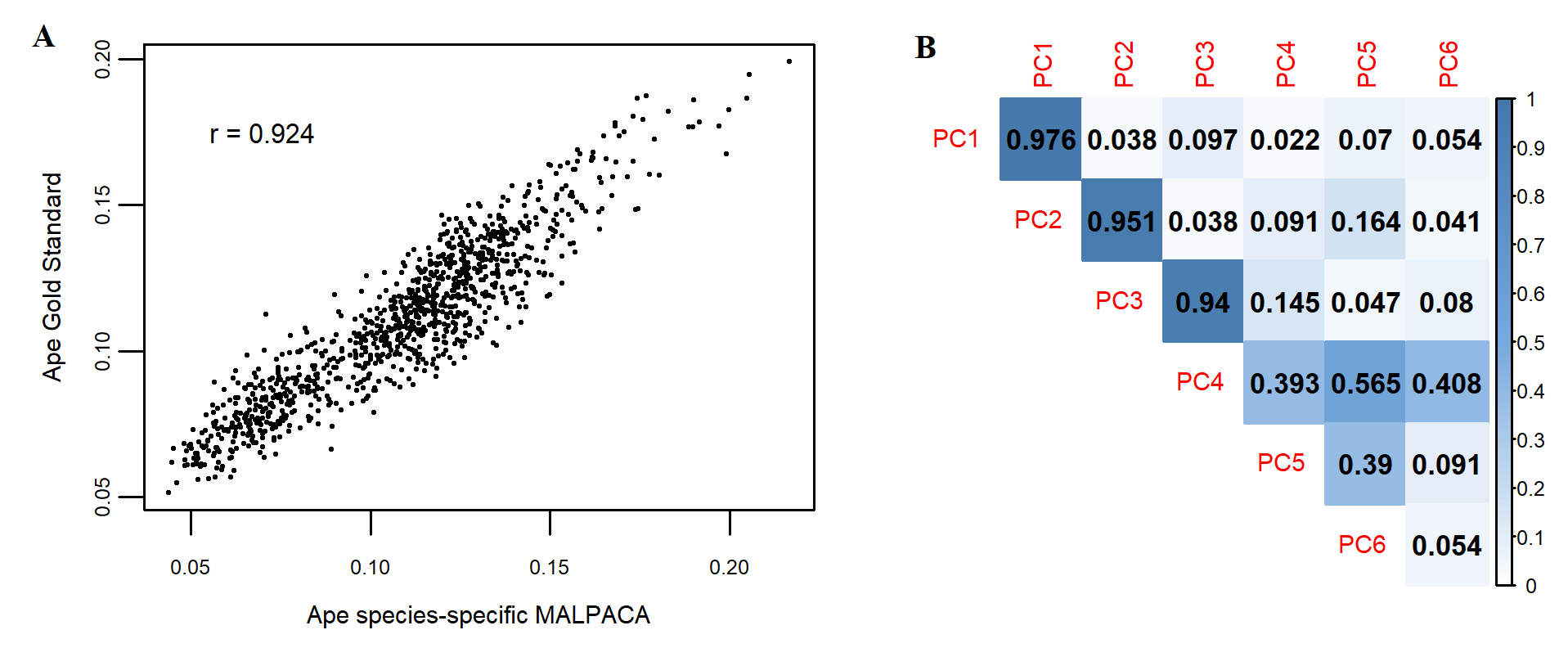

Supplement: S8 Fig — (A) Correlation in the pairwise Procrustes distances derived from species-specific MALPACA and manual landmarks. (B) Correlations in PC scores derived from species-specific MALPACA and manual landmarks. Each grid represents the correlation coefficient between a PC from an automated landmarking analysis and a PC from the Gold Standard, depending on its row and column names. (TIF) [file pone.0278035.s009.tif]
