# Supplementary material for: Automated landmarking via multiple templates
Source: PLoS One. 2022 Dec 1;17(12):e0278035. doi: 10.1371/journal.pone.0278035 (PMC9714854; doi:10.1371/journal.pone.0278035)
Supplement: S1 Table — Sample size = 61. The K-means selected templates are bold and marked by “template”. (DOCX) [file pone.0278035.s010.docx]

| Mouse ID | Mouse ID | Mouse ID |
| --- | --- | --- |
| **129S1.SVIMJ (Template)** | C57BL.10J | NOD.SHILTJ |
| 129X1.SVJ | C57BL6.J | NOR.LTJ |
| A.J. | C57BLKS.J | NU.J |
| Ark.J | C57L.J | NZB.BINJ |
| B6AF1.J | CAF1.J | NZBWF1.J |
| B6C3F1.J | **CAST.EIJ (Template)** | NZO.HiLtJ |
| **B6CBAF1.J (Template)** | CB6F1.J | NZW.LACJ |
| B6D2F1.J | CBA.CAJ | PERC.EiJ |
| B6FVBF1.J | CBA.J | PL.J |
| B6SJLF1.J | CZECHII.EIJ | PWD.PhJ |
| B6129PF1.J | DBA.1J | PWK.PhJ |
| B6129SF1.J | DBA.2J | **SF.CamEiJ (Template)** |
| **BALB.CBYJ (Template)** | FVB.NJ | SJL.J |
| BALB.CH | I.LNJ | SKIVE.EiJ |
| BTBR.T.Itpr3tf | KK.HIJ | SM.J |
| BUB.BNJ | LEWES.J | **SPRET.EiJ (Template)** |
| C3D2F1.J | LG.J | SWR.J |
| C3H.HEJ | LP.J | TALLYHO.JNGJ |
| C3H.HEOUJ | MOLF.EiJ | **X129P3.J (Template)** |
| C3HEB.FEJ | MOLG.DnJ |  |
| C57BL.6NJ | MRL.MPJ |  |
