# Supplementary material for: Automated landmarking via multiple templates
Source: PLoS One. 2022 Dec 1;17(12):e0278035. doi: 10.1371/journal.pone.0278035 (PMC9714854; doi:10.1371/journal.pone.0278035)
Supplement: S2 Table — Sample size = 52. The two templates selected by K-means per species (six templates total) are bold and marked by “template” in the parenthesis. The remaining 46 specimens are being landmarked. (DOCX) [file pone.0278035.s011.docx]

| *Pan troglodytes* | *Gorilla gorilla* | *Pongo pygmaeus* |
| --- | --- | --- |
| **USNM084655 (Template)** | USNM174715 | **USNM142185 (Templates)** |
| USNM174701 | USNM174722 | USNM142188 |
| USNM174703 | USNM176209 | USNM142189 |
| USNM174704 | USNM176211 | USNM142194 |
| USNM174707 | USNM176216 | USNM145300 |
| USNM174710 | USNM176217 | USNM145302 |
| USNM176228 | USNM176219 | USNM145303 |
| **USNM176236 (Template)** | USNM220060 | USNM145307 |
| USNM220062 | USNM220324 | USNM145308 |
| USNM220063 | USNM252575 | USNM145309 |
| USNM220065 | USNM252577 | USNM153805 |
|  | USNM252578 | USNM153806 |
|  | USNM252580 | USNM153822 |
|  | USNM297857 | USNM153824 |
|  | USNM582726 | **USNM153830 (Template)** |
|  | USNM590942 | USNM197664 |
|  | USNM590947 | USNM399047 |
|  | USNM590951 | USNM588109 |
|  | **USNM590953 (Template)** |  |
|  | USNM590954 |  |
|  | USNM599165 |  |
|  | USNM599166 |  |
|  | **USNM599167 (Template)** |  |
