# Supplementary material for: Automated landmarking via multiple templates
Source: PLoS One. 2022 Dec 1;17(12):e0278035. doi: 10.1371/journal.pone.0278035 (PMC9714854; doi:10.1371/journal.pone.0278035)
Supplement: S3 Table — MALPACA uses the default settings listed in the ALPACA module of the SlicerMorph extension. See Porto et al. [10] for detail. Spacing factor determines the point cloud density for K-means. See section 2 of the supplementary material for details. In general, the settings ensure that each point cloud used for the K-means template selection has 700 to 800 points. Also see S1 Appendix for explanations of these settings. (DOCX) [file pone.0278035.s012.docx]

| **MALPACA** | **Spacing Factor (K-means)** | **Reference** | **Iterations** | **Set seed?** |
| --- | --- | --- | --- | --- |
| Mouse sample | 0.4 | 129S1.SVIMJ | 10,000 | Yes |
| Ape sample | 0.3 | USNM084655 | 10,000 | Yes |
