# Supplementary material for: Automated landmarking via multiple templates
Source: PLoS One. 2022 Dec 1;17(12):e0278035. doi: 10.1371/journal.pone.0278035 (PMC9714854; doi:10.1371/journal.pone.0278035)
Supplement: S4 Table — One-sided t-tests compare whether mouse MALPACA RMSEs significantly smaller than ALPACA RMSEs using the synthetic template and individual mouse template. (DOCX) [file pone.0278035.s013.docx]

| Mouse MALPACA one-sided Welch t-test for RMSEs | p-value |
| --- | --- |
| Vs. ALPACA (synthetic template) | 1.283 × 10^-16^ |
| Vs. 129S1.SVIMJ ALPACA | 8.493 × 10^-10^ |
| Vs. B6CBAF1 ALPACA | 8.522 × 10^-7^ |
| Vs. BALB.CBYJ ALPACA | 2.539 × 10^-16^ |
| Vs. CAST.EIJ ALPACA | 3.847 × 10^-28^ |
| Vs. SF.CAMEIJ ALPACA | 3.496 × 10^-36^ |
| Vs. SPRET.EIJ ALPACA | 5.313 × 10^-35^ |
| Vs. X129P3.J ALPACA | 2.211 × 10^-16^ |
