# Supplementary material for: Automated landmarking via multiple templates
Source: PLoS One. 2022 Dec 1;17(12):e0278035. doi: 10.1371/journal.pone.0278035 (PMC9714854; doi:10.1371/journal.pone.0278035)
Supplement: S5 Table — MALPACA errors versus manual landmarks significantly smaller than ALPACA errors in the 37 landmarks listed. (DOCX) [file pone.0278035.s014.docx]

| Landmark (Mouse) | p-value | Landmark (Mouse) | p-value |
| --- | --- | --- | --- |
| 1 | 7.262 × 10^-5^ | 28 | 0.007361715 |
| 2 | 1.738 × 10^-6^ | 30 | 0.00246 |
| 3 | 3.674 × 10^-6^ | 31 | 0.00107 |
| 4 | 0.0175 | 32 | 1.305 × 10^-36^ |
| 5 | 0.0256 | 33 | 7.705 × 10^-40^ |
| 7 | 0.00738 | 35 | 2.802 × 10^-5^ |
| 9 | 0.0315 | 36 | 0.000229 |
| 10 | 0.0324 | 37 | 0.0410 |
| 11 | 0.000713 | 38 | 0.00968 |
| 12 | 0.00369 | 39 | 0.000966 |
| 13 | 0.00373 | 41 | 0.00153 |
| 14 | 5.728 × 10^-5^ | 44 | 0.00636 |
| 18 | 0.00198 | 45 | 0.0190 |
| 19 | 0.0256 | 46 | 2.342 × 10^-15^ |
| 21 | 0.0419 | 48 | 1.554 × 10^-14^ |
| 22 | 3.912 × 10^-9^ | 49 | 1.377 × 10^-9^ |
| 25 | 0.00139 | 50 | 0.0005536854 |
| 26 | 0.000737 | 51 | 0.001063129 |
| 27 | 0.0305 |  |  |
