# Supplementary material for: Automated landmarking via multiple templates
Source: PLoS One. 2022 Dec 1;17(12):e0278035. doi: 10.1371/journal.pone.0278035 (PMC9714854; doi:10.1371/journal.pone.0278035)
Supplement: S6 Table — Only listing landmarks not listed in S5 Table, i.e., MALPACA-derived estimates do not show significantly smaller errors than ALPACA ones in these 14 landmarks according to the one-sided t-test in S5 Table. (DOCX) [file pone.0278035.s015.docx]

| **Landmark (Mouse)** | **p-value** | **Landmark (Mouse)** | **p-value** |
| --- | --- | --- | --- |
| 6 | 0.379 | 24 | 0.886 |
| 8 | 0.505 | 29 | 0.774 |
| 15 | 0.359 | 34 | 0.927 |
| 16 | 0.934 | 40 | 0.332 |
| 17 | 0.765 | 42 | 0.365 |
| 20 | 0.396 | 43 | 0.819 |
| 23 | 0.716 | 47 | 0.806 |
