# Supplementary material for: Automated landmarking via multiple templates
Source: PLoS One. 2022 Dec 1;17(12):e0278035. doi: 10.1371/journal.pone.0278035 (PMC9714854; doi:10.1371/journal.pone.0278035)
Supplement: S7 Table — (DOCX) [file pone.0278035.s016.docx]

|  | **Correlation coefficients** |
| --- | --- |
| MALPACA | 0.997 |
| ALPACA (Synthetic template) | 0.993 |
| 129S1.SVIMJ ALPACA | 0.996 |
| B6CBAF1 ALPACA | 0.994 |
| BALB.CBYJ ALPACA | 0.996 |
| CAST.EIJ ALPACA | 0.995 |
| SF.CAMEIJ ALPACA | 0.995 |
| SPRET.EIJ ALPACA | 0.994 |
| X129P3.J ALPACA | 0.995 |
