# Supplementary material for: Automated landmarking via multiple templates
Source: PLoS One. 2022 Dec 1;17(12):e0278035. doi: 10.1371/journal.pone.0278035 (PMC9714854; doi:10.1371/journal.pone.0278035)
Supplement: S8 Table — One-sided t-tests compare whether ape MALPACA RMSEs significantly smaller than ALPACA RMSEs using an individual ape template. (DOCX) [file pone.0278035.s017.docx]

| **Ape 6-template MALPACA RMSEs** | **p-value** |
| --- | --- |
| Vs. Pan 1 template USNM084655 ALPACA | 3.152 × 10^-7^ |
| Vs. Pan 2 template USNM176236 ALPACA | 1.306 × 10^-7^ |
| Vs. Gorilla 1 template USNM590953 ALPACA | 0.0179 |
| Vs. Gorilla2 template USNM599167 ALPACA | 4.329 × 10^-5^ |
| Vs. Pongo 1 template USNM142185 ALPACA | 1.817 × 10^-13^ |
| Vs. Pongo 2 template USNM153830 ALPACA | 2.738 × 10^-6^ |
