# Supplementary material for: Automated landmarking via multiple templates
Source: PLoS One. 2022 Dec 1;17(12):e0278035. doi: 10.1371/journal.pone.0278035 (PMC9714854; doi:10.1371/journal.pone.0278035)
Supplement: S9 Table — (DOCX) [file pone.0278035.s018.docx]

|  | **Correlations with manually placed landmarks in centroid sizes** |
| --- | --- |
| MALPACA | 0.999 |
| Pan 1 ALPACA (USNM084655) | 0.997 |
| Pan 2 ALPACA (USNM176236) | 0.998 |
| Gorilla 1 ALPACA (USNM590953) | 0.998 |
| Gorilla 2 ALPACA (USNM599167) | 0.997 |
| Pongo 1 ALPACA (USNM142185) | 0.997 |
| Pongo 2 ALPACA(USNM153830) | 0.997 |
| Species-specific MALPACA | 0.994 |
