# Supplementary material for: Automated landmarking via multiple templates
Source: PLoS One. 2022 Dec 1;17(12):e0278035. doi: 10.1371/journal.pone.0278035 (PMC9714854; doi:10.1371/journal.pone.0278035)
Supplement: S10 Table — (DOCX) [file pone.0278035.s019.docx]

| **Landmark (ape)** | **P-values** | **Landmark (ape)** | **P-values** |
| --- | --- | --- | --- |
| 1 | 3.254 × 10^-8^ | 22 | 8.522 × 10^-8^ |
| 2 | 2.0607 × 10^-11^ | 23 | 8.463 × 10^-7^ |
| 3 | 0.000681 | 24 | 1.405 × 10^-6^ |
| 4 | 0.0491 | 25 | 3.940 × 10^-6^ |
| 5 | 1.952 × 10^-5^ | 26 | 0.4099142 |
| 6 | 2.225 × 10^-6^ | 27 | 0.2822061 |
| 7 | 3.960 × 10^-7^ | 28 | 4.081 × 10^-5^ |
| 8 | 4.316 × 10^-13^ | 29 | 4.869 × 10^-6^ |
| 9 | 1.015 × 10^-15^ | 30 | 7.860 × 10^-8^ |
| 10 | 1.205 × 10^-7^ | 31 | 4.909 × 10^-6^ |
| 11 | 7.325 × 10^-10^ | 32 | 7.872 × 10^-11^ |
| 12 | 0.00415 | 33 | 3.388 × 10^-8^ |
| 13 | 3.233 × 10^-5^ | 34 | 3.688 × 10^-17^ |
| 14 | 1.093 × 10^-5^ | 35 | 4.092 × 10^-18^ |
| 15 | 0.00520 | 36 | 1.908 × 10^-5^ |
| 16 | 4.318 × 10^-10^ | 37 | 3.464 × 10^-6^ |
| 17 | 7.573 × 10^-11^ | 38 | 9.577 × 10^-13^ |
| 18 | 0.227 | 39 | 1.183 × 10^-13^ |
| 19 | 1.562 × 10^-8^ | 40 | 1.218 × 10^-14^ |
| 20 | 0.000237 | 41 | 3.310 × 10^-13^ |
| 21 | 5.922 × 10^-5^ |  |  |
