# Supplementary material for: Automated landmarking via multiple templates
Source: PLoS One. 2022 Dec 1;17(12):e0278035. doi: 10.1371/journal.pone.0278035 (PMC9714854; doi:10.1371/journal.pone.0278035)
Supplement: S11 Table — The values in the paratheses are corresponding quantile values of permutation analyses. (DOCX) [file pone.0278035.s020.docx]

|  | **0th** | **25th** | **50th** | **75th** | **100th** |
| --- | --- | --- | --- | --- | --- |
| Mouse MALPACA RMSEs | 0.177  (0.159) | 0.237  (0.231) | 0.261  (0.255) | 0.285  (0.286) | 0.479  (0.508) |
| Ape 6-template MALPACA RMSEs | 2.382  (2.078) | 3.172  (3.087) | 3.833  (3.747) | 4.437  (4.689) | 6.153  (6.713) |
