# Supplementary material for: Automated landmarking via multiple templates
Source: PLoS One. 2022 Dec 1;17(12):e0278035. doi: 10.1371/journal.pone.0278035 (PMC9714854; doi:10.1371/journal.pone.0278035)
Supplement: S1 File — Each graph shows the boxplot of distances between individual estimates of one ape specimen to the corresponding MALPACA final output. The red horizontal line represents the threshold, which is two standard deviations above the mean of the distances for that specimen. (PDF) [file pone.0278035.s021.pdf]

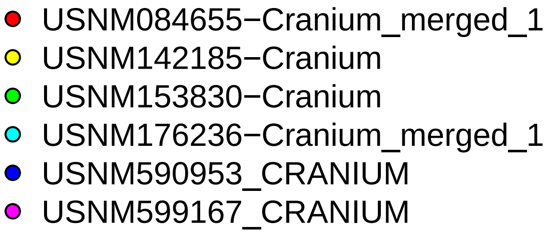
USNM084655−Cranium_merged_1
USNM142185−Cranium
USNM153830−Cranium
USNM176236−Cranium_merged_1
USNM590953_CRANIUM
USNM599167_CRANIUM

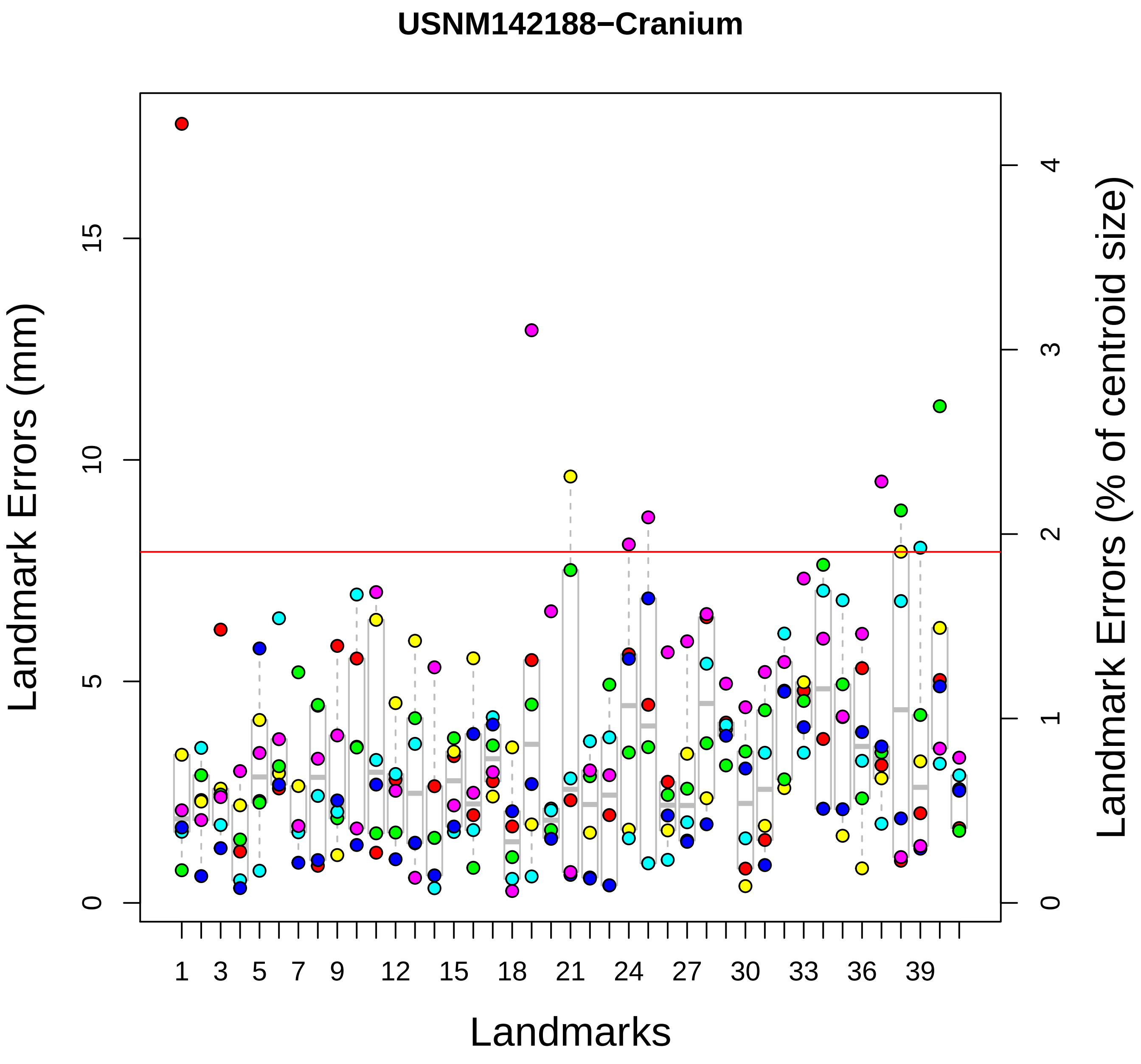
USNM142188−Cranium
Landmark Errors (mm)
Landmark Errors (% of centroid size)
Landmarks
0
5
10
15
0
1
2
3
4
1
3
5
7
9
12
15
18
21
24
27
30
33
36
39

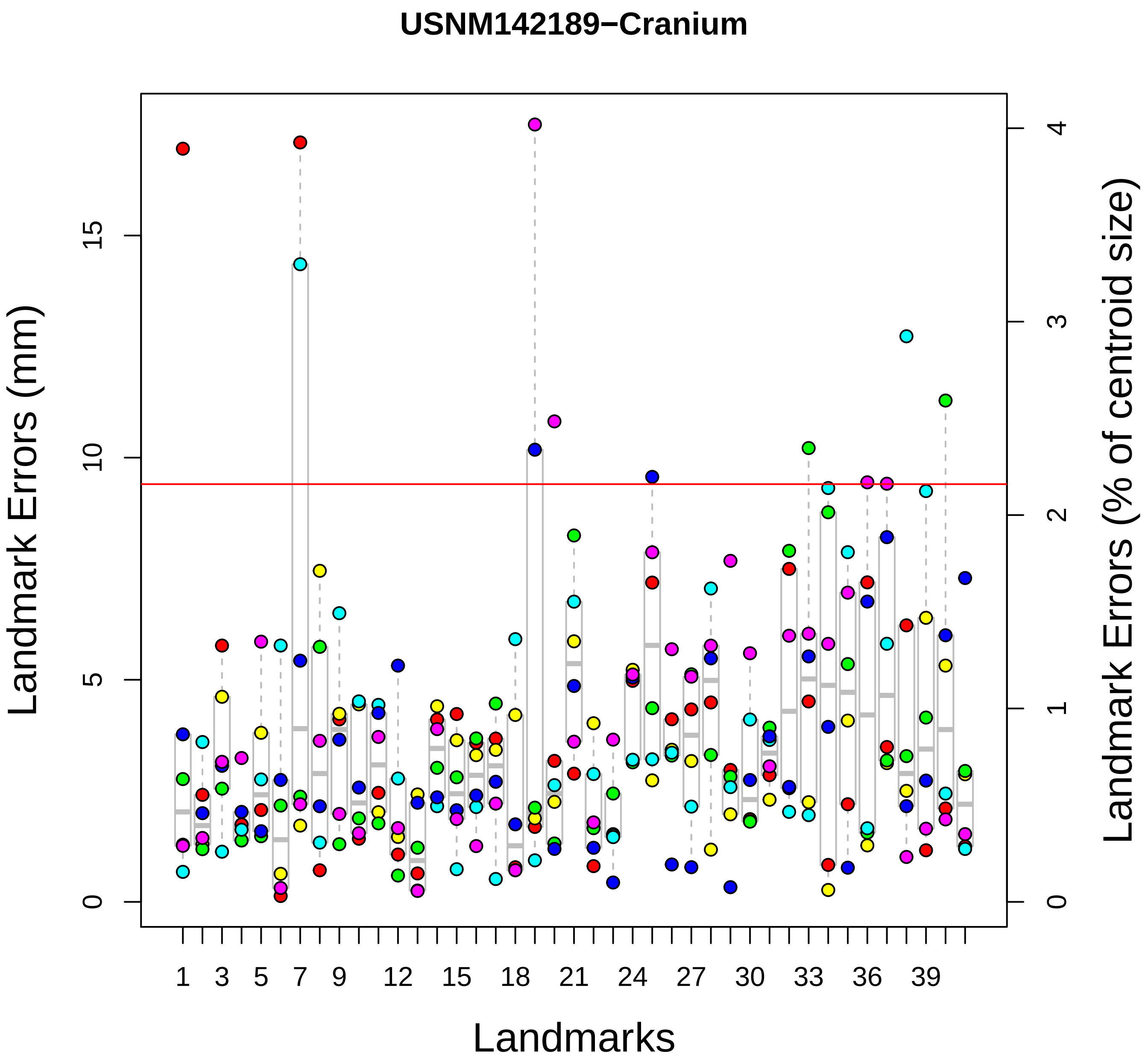
USNM142189−Cranium
Landmark Errors (mm)
Landmark Errors (% of centroid size)
Landmarks
0
5
10
15
0
1
2
3
4
1
3
5
7
9
12
15
18
21
24
27
30
33
36
39

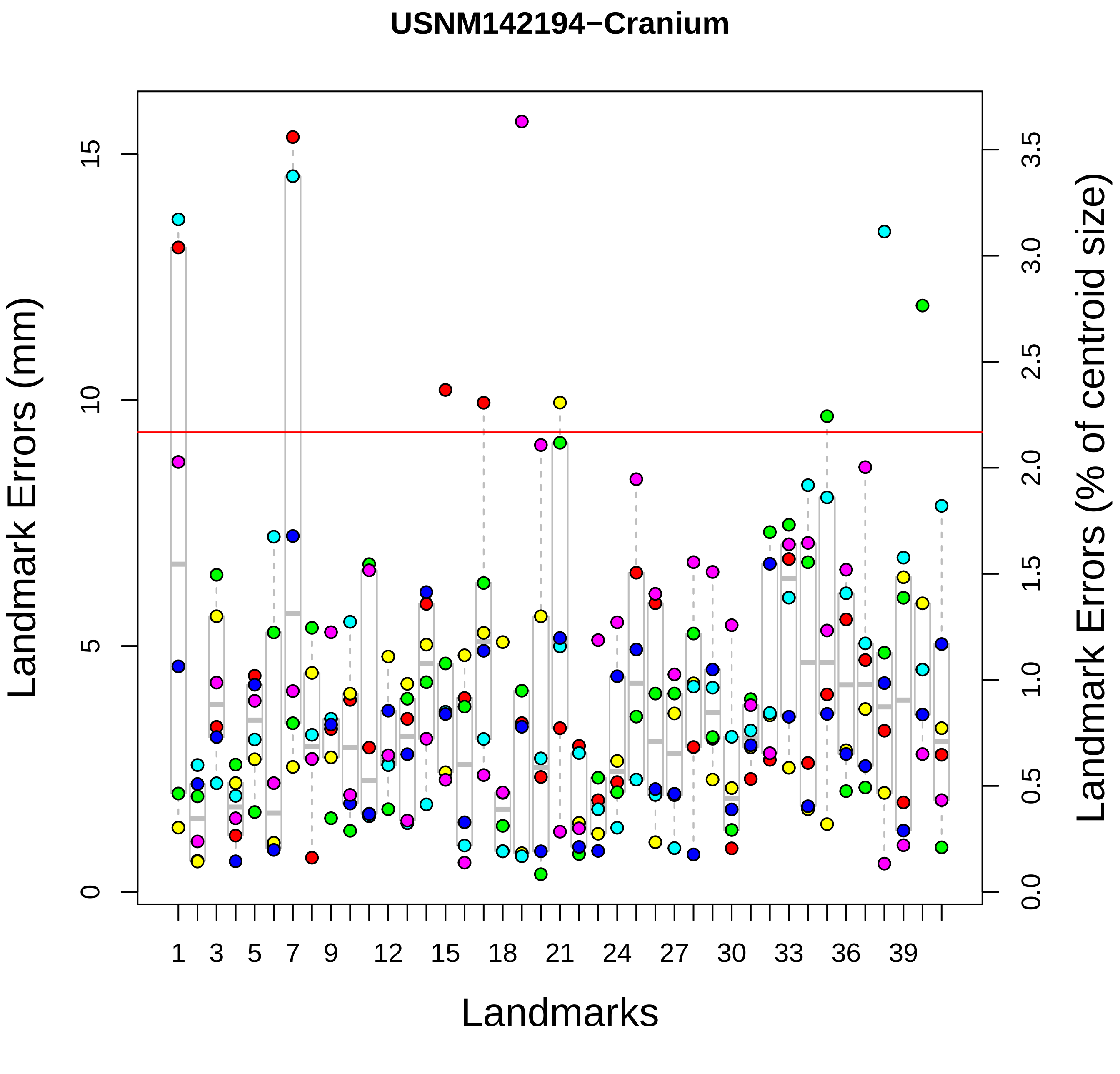
USNM142194−Cranium
Landmark Errors (mm)
Landmark Errors (% of centroid size)
Landmarks
0
5
10
15
0.0
0.5
1.0
1.5
2.0
2.5
3.0
3.5
1
3
5
7
9
12
15
18
21
24
27
30
33
36
39

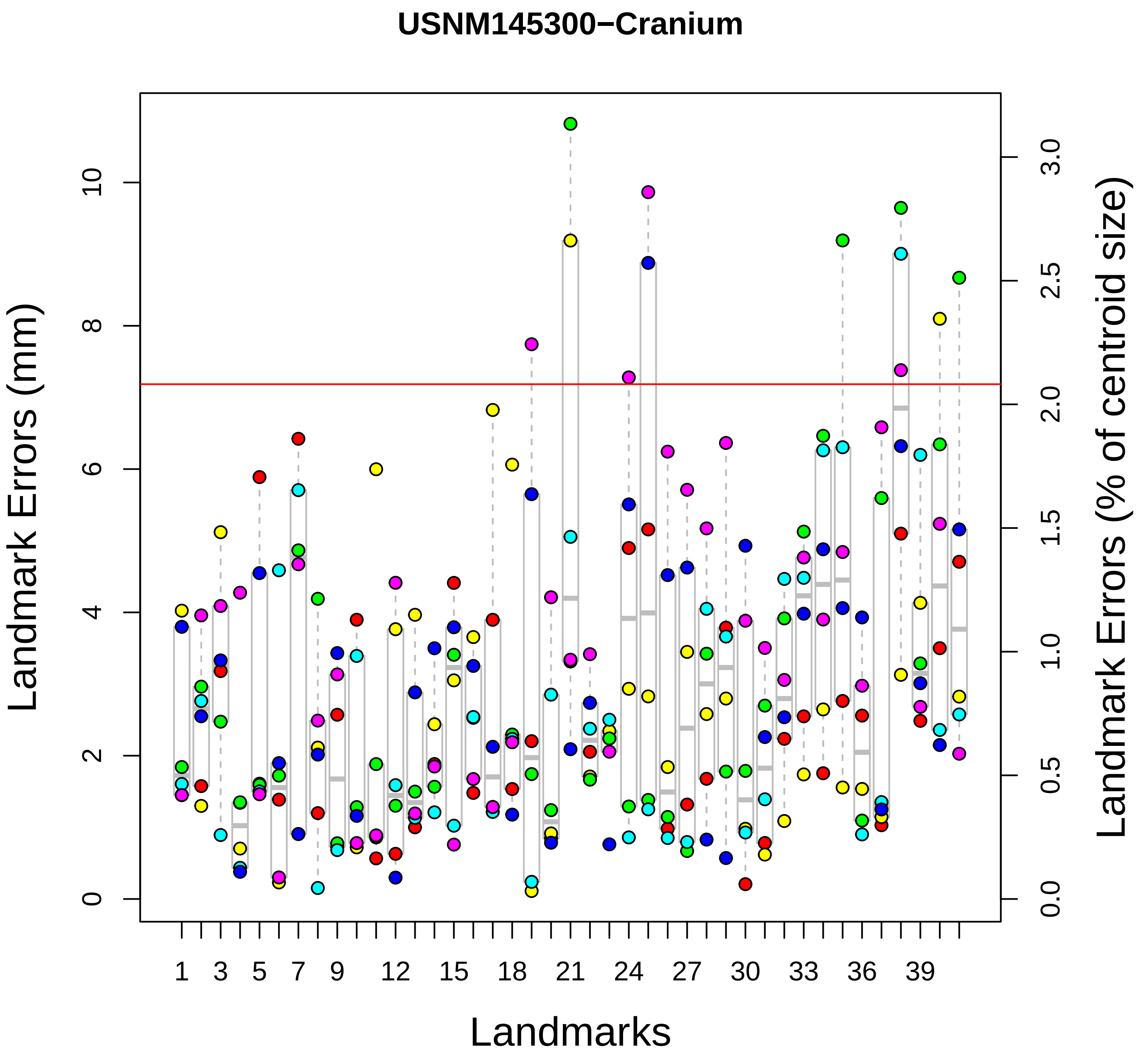

USNM145300−Cranium
Landmark Errors (mm)
Landmark Errors (% of centroid size)
Landmarks
0
2
4
6
8
10
0.0
0.5
1.0
1.5
2.0
2.5
3.0
1
3
5
7
9
12
15
18
21
24
27
30
33
36
39

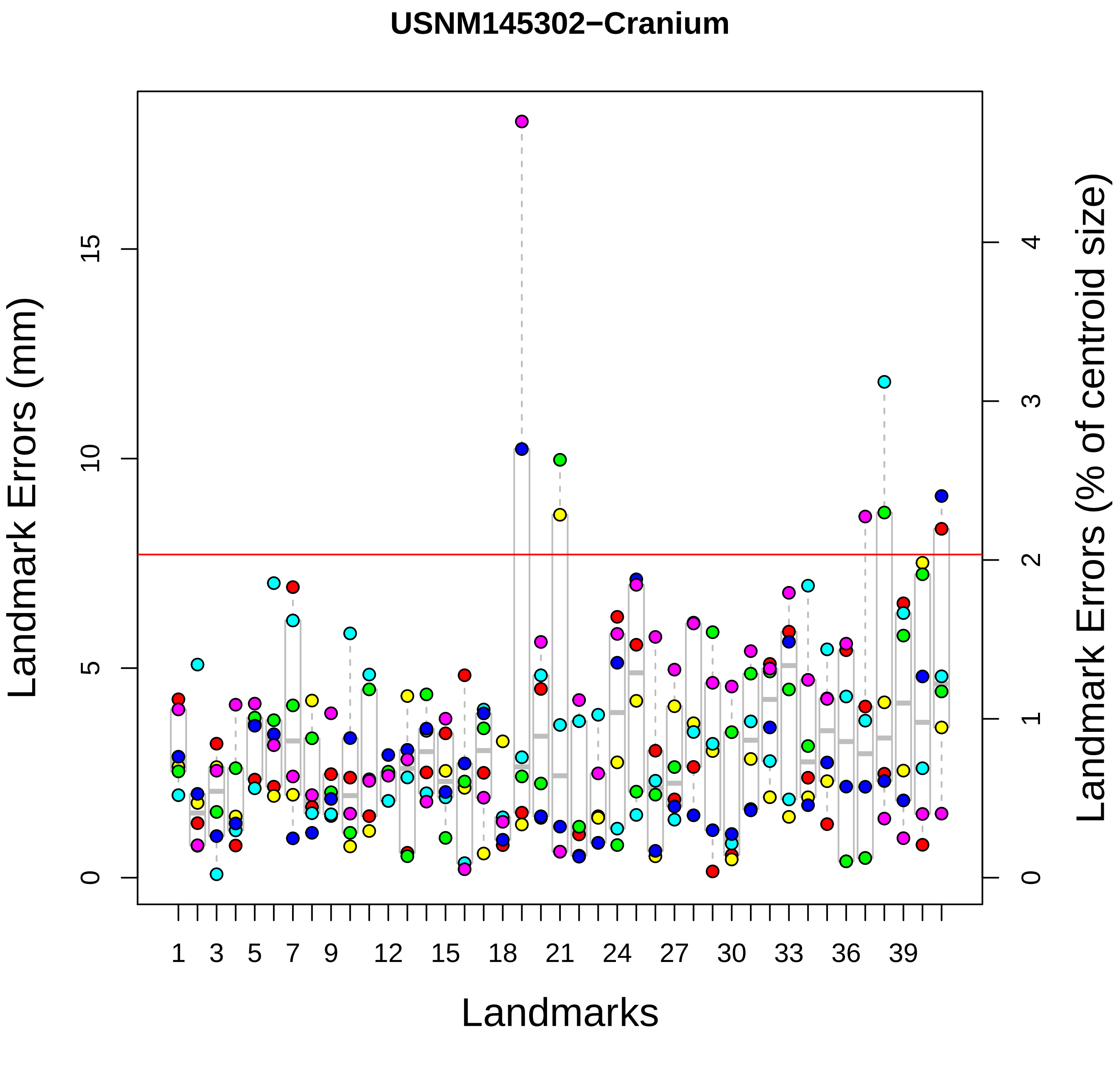

USNM145302−Cranium
Landmark Errors (mm)
Landmark Errors (% of centroid size)
Landmarks
0
5
10
15
0
1
2
3
4
1
3
5
7
9
12
15
18
21
24
27
30
33
36
39

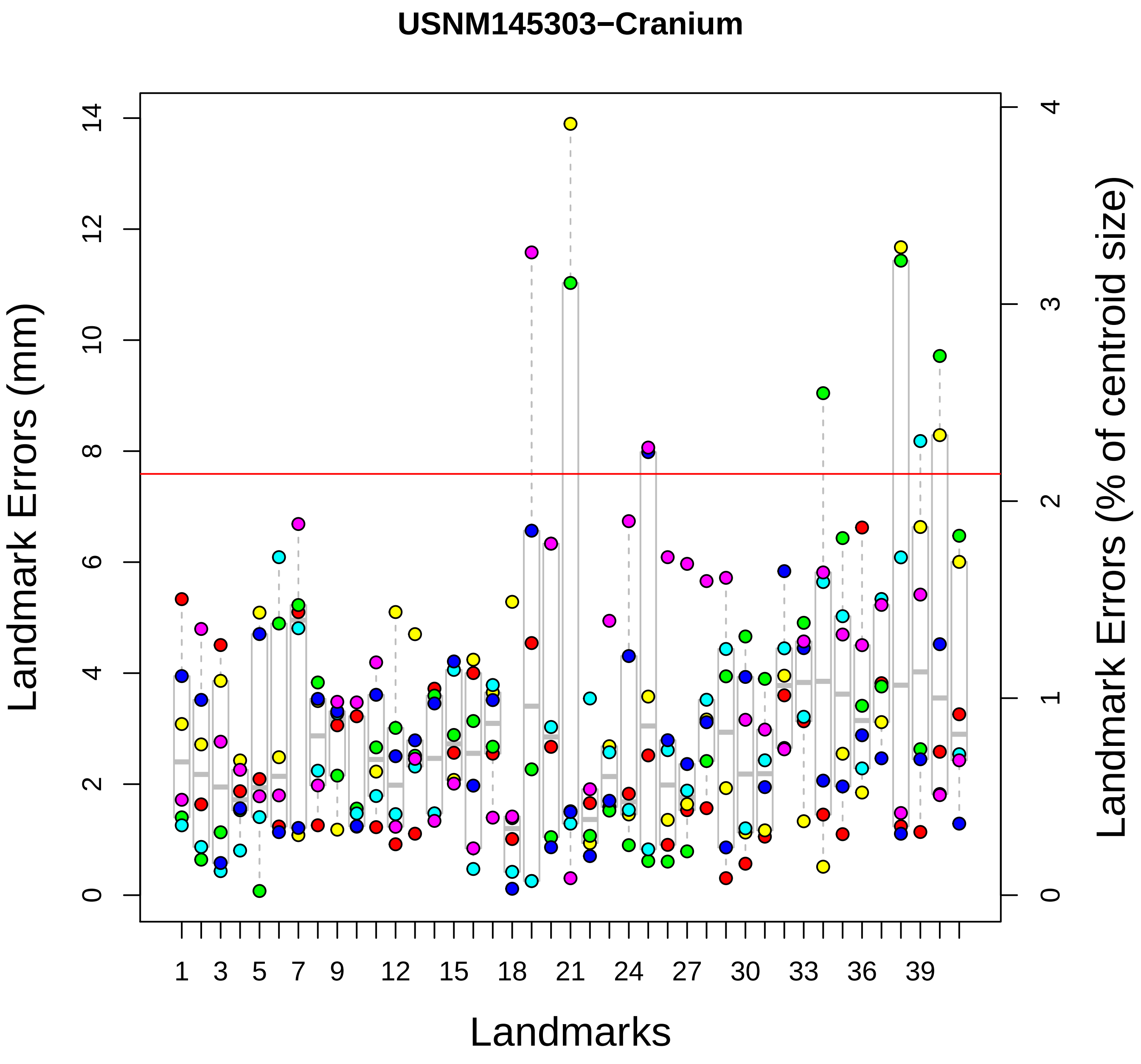
USNM145303−Cranium
Landmark Errors (mm)
Landmark Errors (% of centroid size)
Landmarks
0
2
4
6
8
10
12
14
0
1
2
3
4
1
3
5
7
9
12
15
18
21
24
27
30
33
36
39

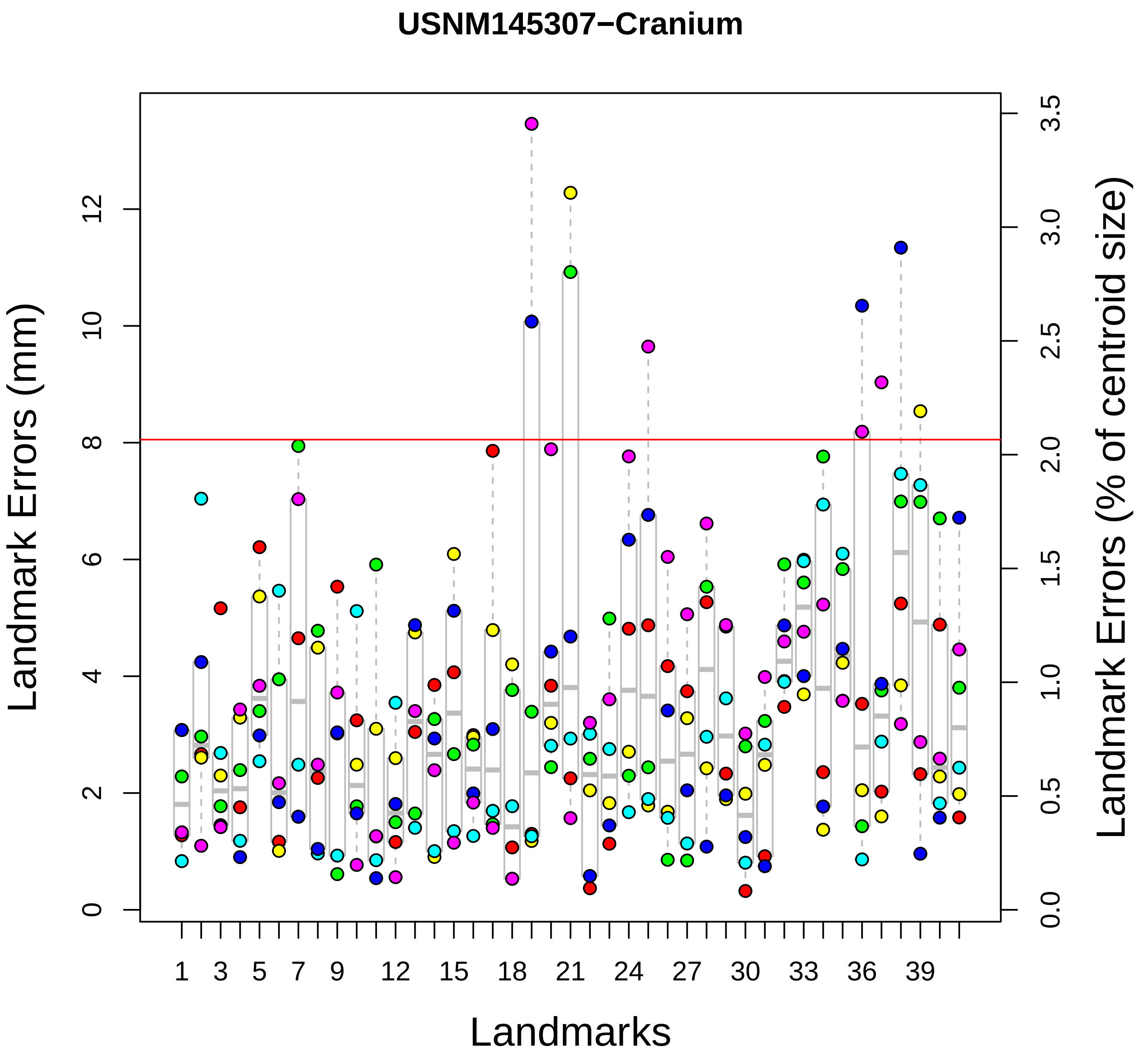
USNM145307−Cranium
Landmark Errors (mm)
Landmark Errors (% of centroid size)
Landmarks
0
2
4
6
8
10
12
0.0
0.5
1.0
1.5
2.0
2.5
3.0
3.5
1
3
5
7
9
12
15
18
21
24
27
30
33
36
39

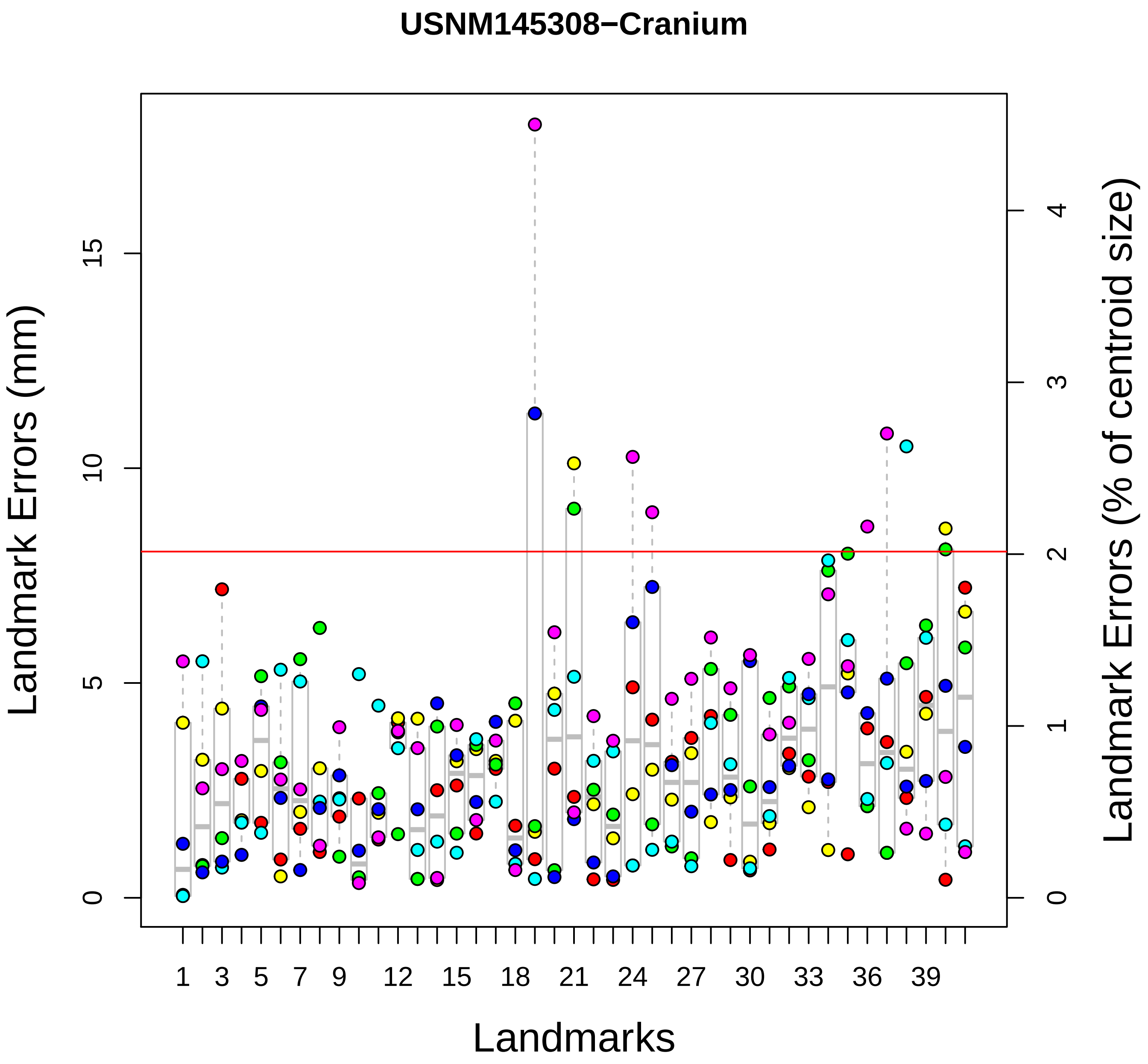

USNM145308−Cranium
Landmark Errors (mm)
Landmark Errors (% of centroid size)
Landmarks
0
5
10
15
0
1
2
3
4
1
3
5
7
9
12
15
18
21
24
27
30
33
36
39

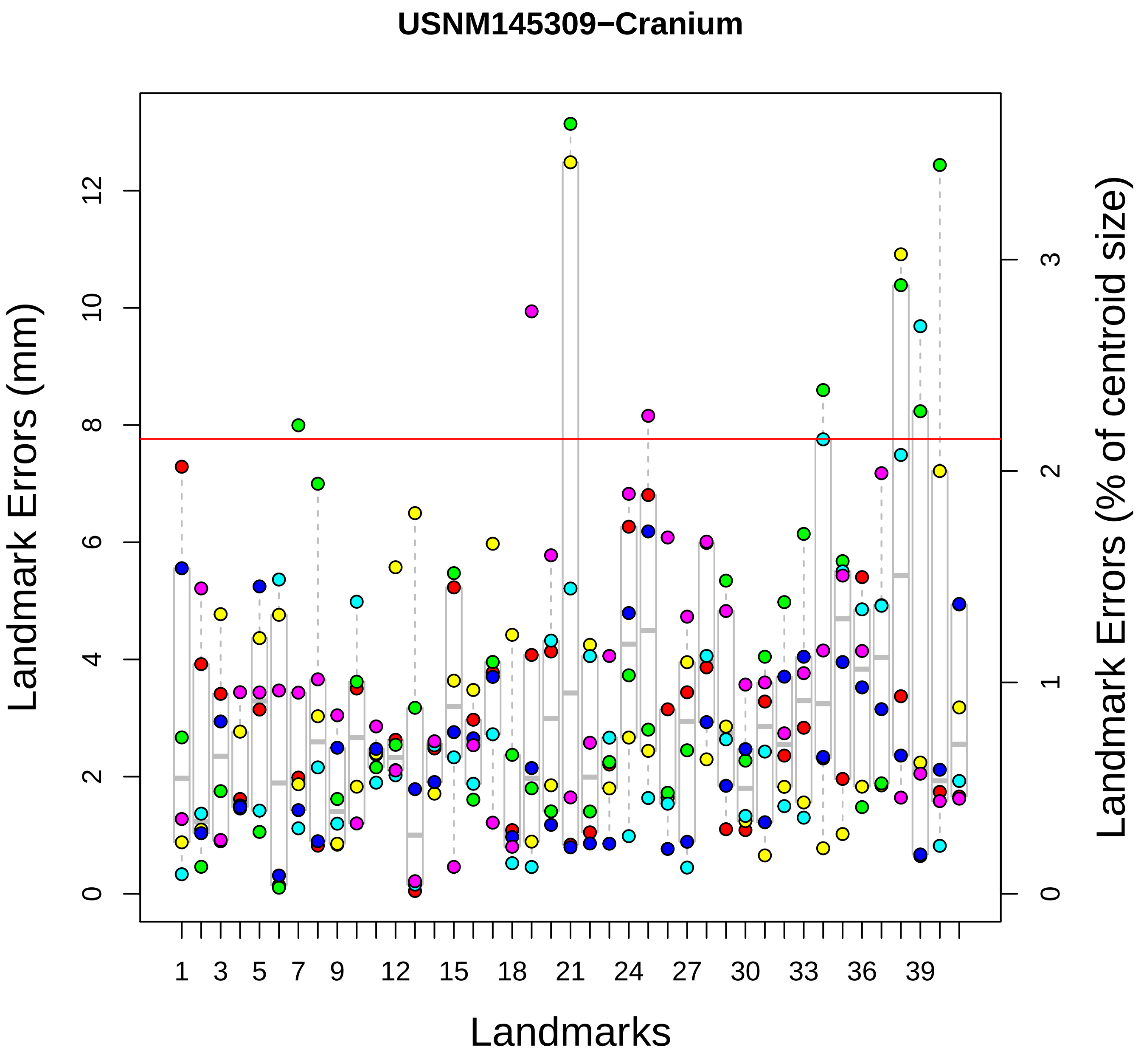

USNM145309−Cranium
Landmark Errors (mm)
Landmark Errors (% of centroid size)
Landmarks
0
2
4
6
8
10
12
0
1
2
3
1
3
5
7
9
12
15
18
21
24
27
30
33
36
39

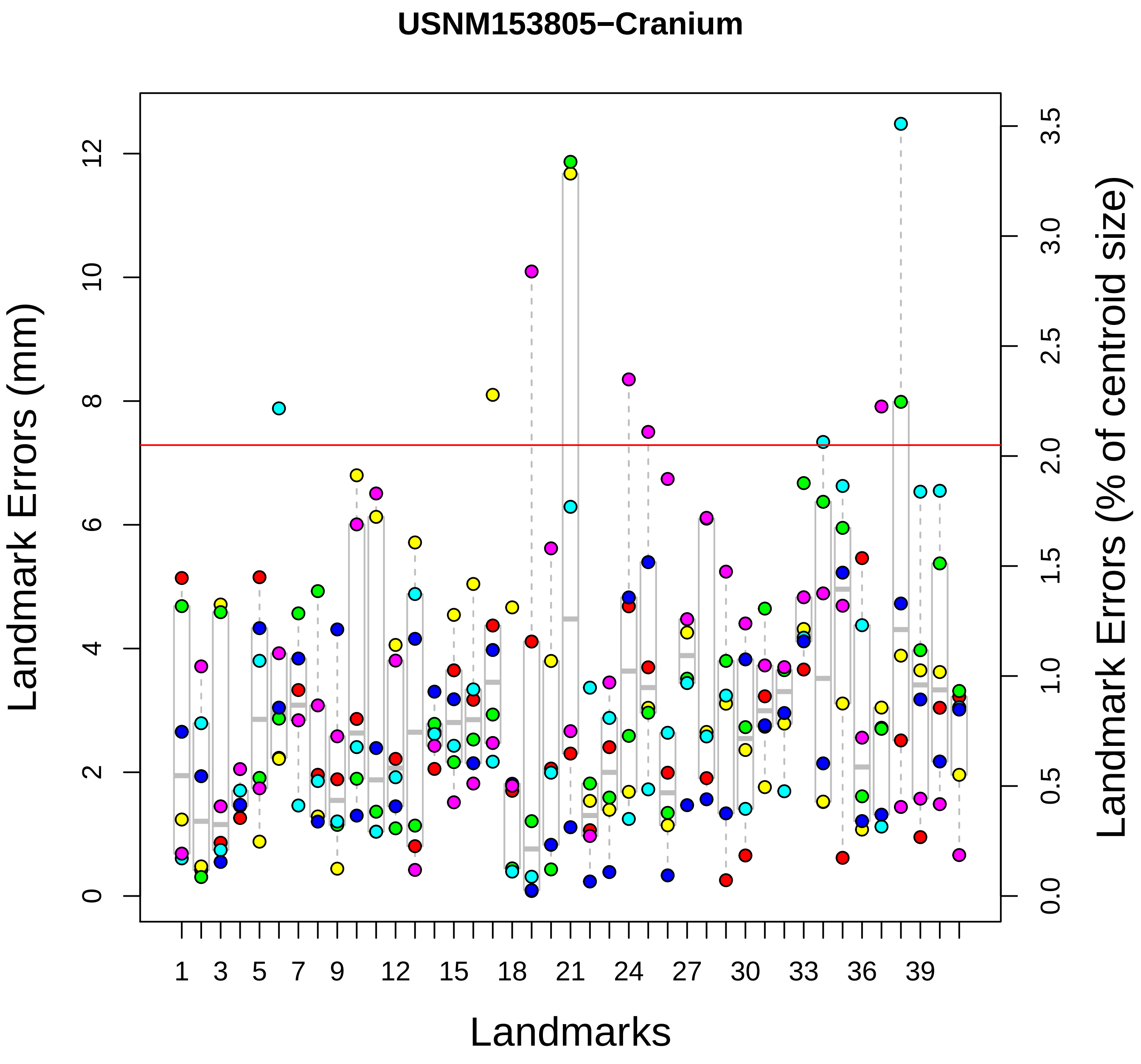
USNM153805−Cranium
Landmark Errors (mm)
Landmark Errors (% of centroid size)
Landmarks
0
2
4
6
8
10
12
0.0
0.5
1.0
1.5
2.0
2.5
3.0
3.5
1
3
5
7
9
12
15
18
21
24
27
30
33
36
39

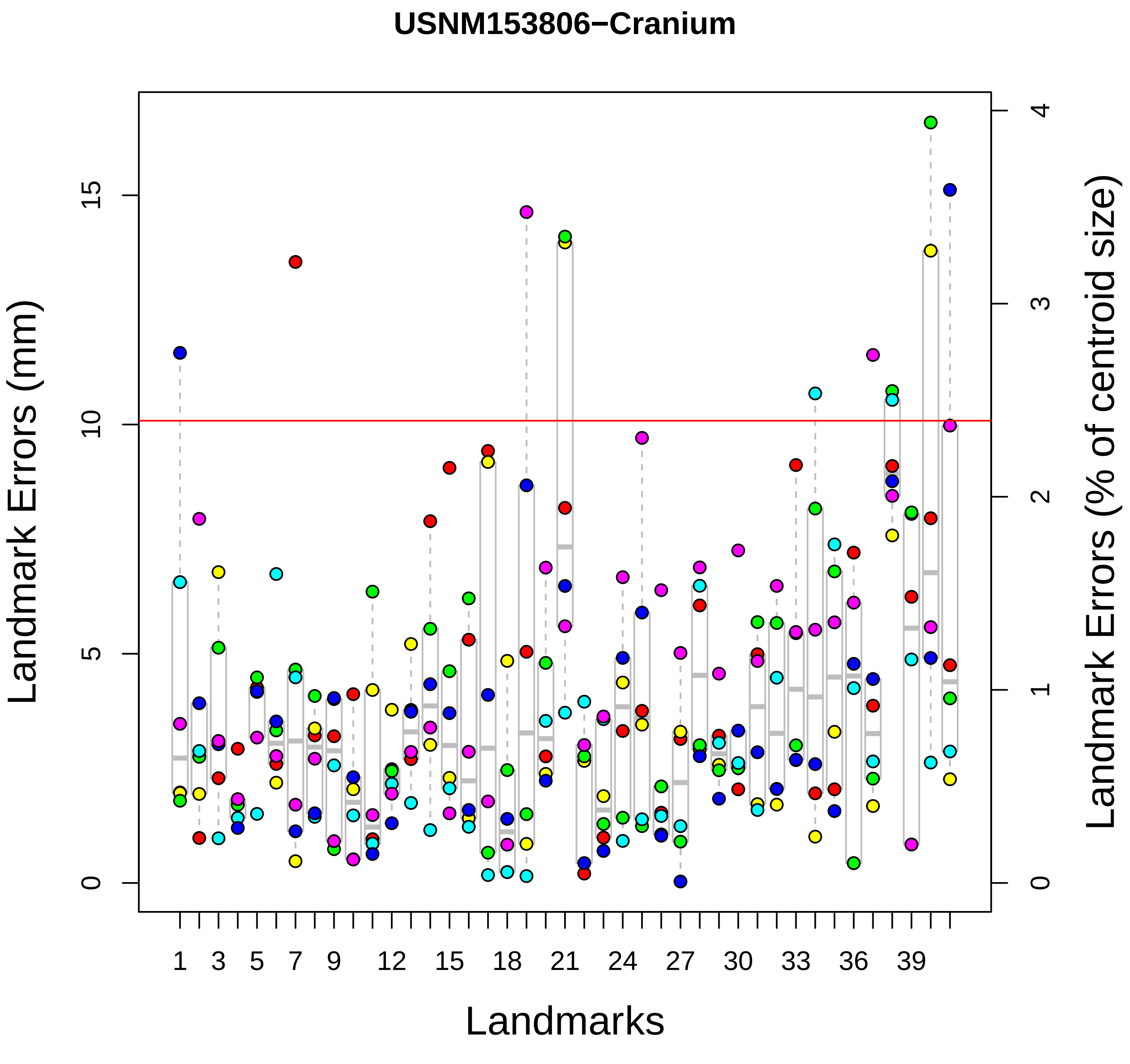

USNM153806−Cranium
Landmark Errors (mm)
Landmark Errors (% of centroid size)
Landmarks
0
5
10
15
0
1
2
3
4
1
3
5
7
9
12
15
18
21
24
27
30
33
36
39

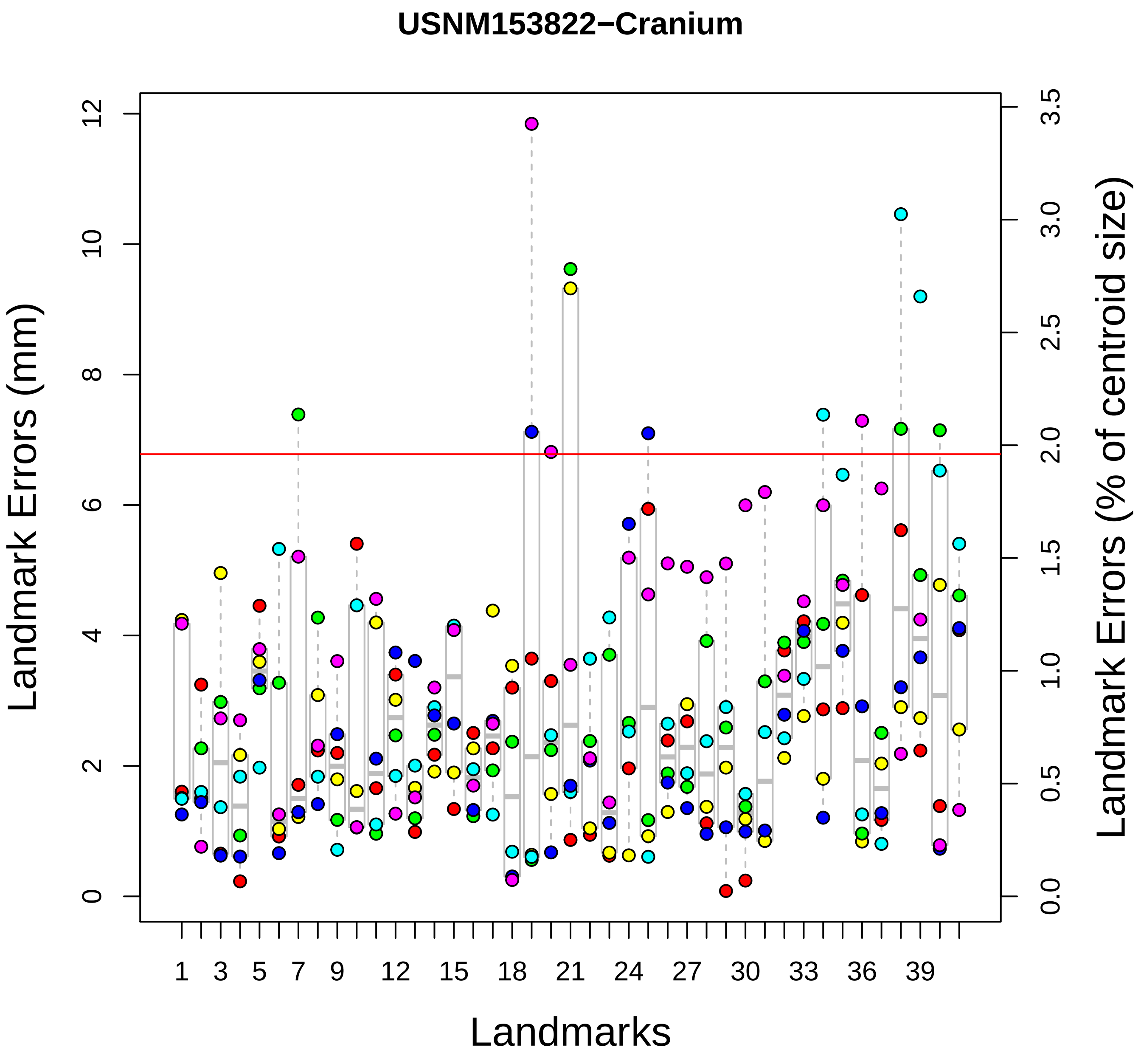
USNM153822−Cranium
Landmark Errors (mm)
Landmark Errors (% of centroid size)
Landmarks
0
2
4
6
8
10
12
0.0
0.5
1.0
1.5
2.0
2.5
3.0
3.5
1
3
5
7
9
12
15
18
21
24
27
30
33
36
39

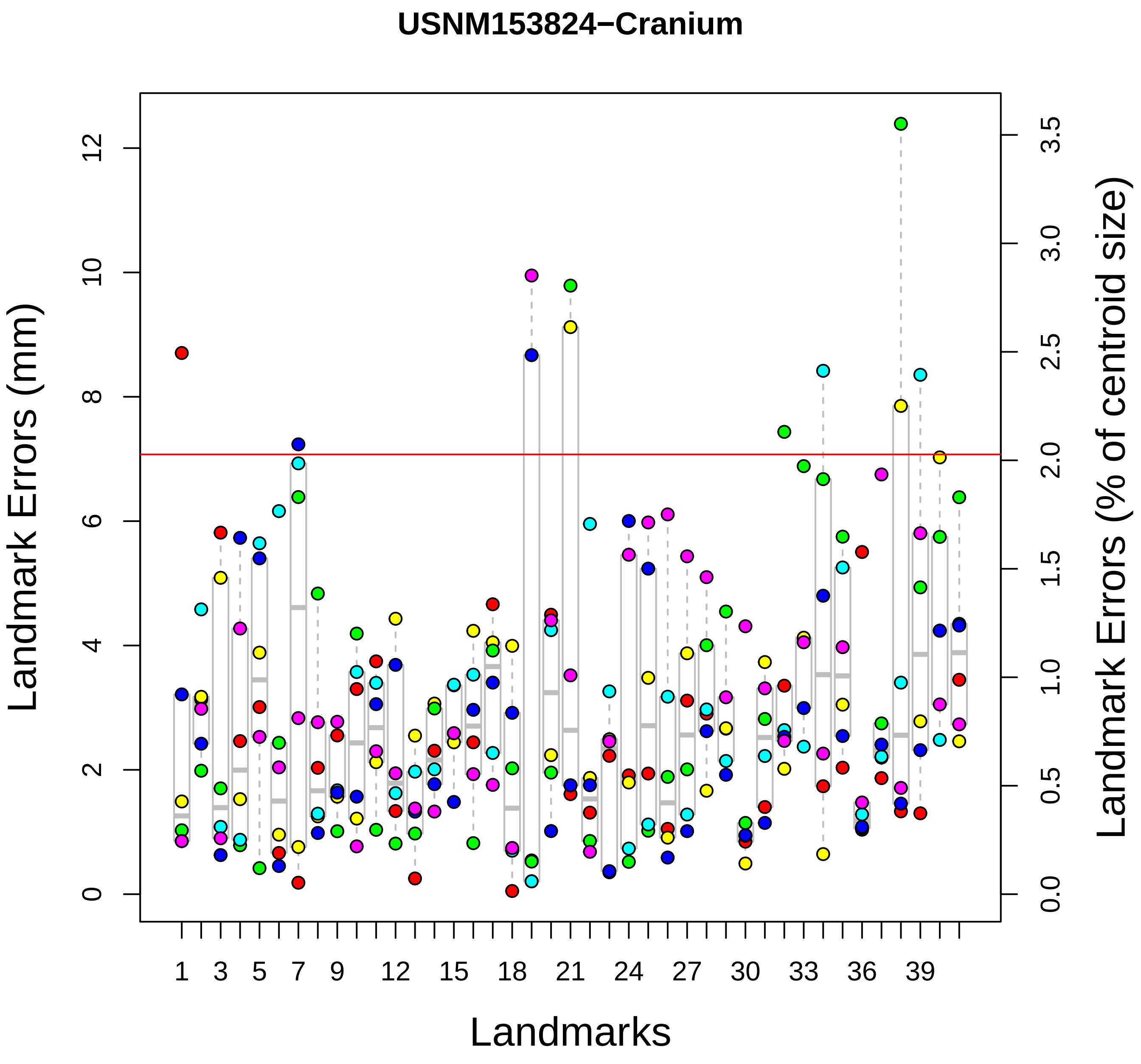
USNM153824−Cranium
Landmark Errors (mm)
Landmark Errors (% of centroid size)
Landmarks
0
2
4
6
8
10
12
0.0
0.5
1.0
1.5
2.0
2.5
3.0
3.5
1
3
5
7
9
12
15
18
21
24
27
30
33
36
39

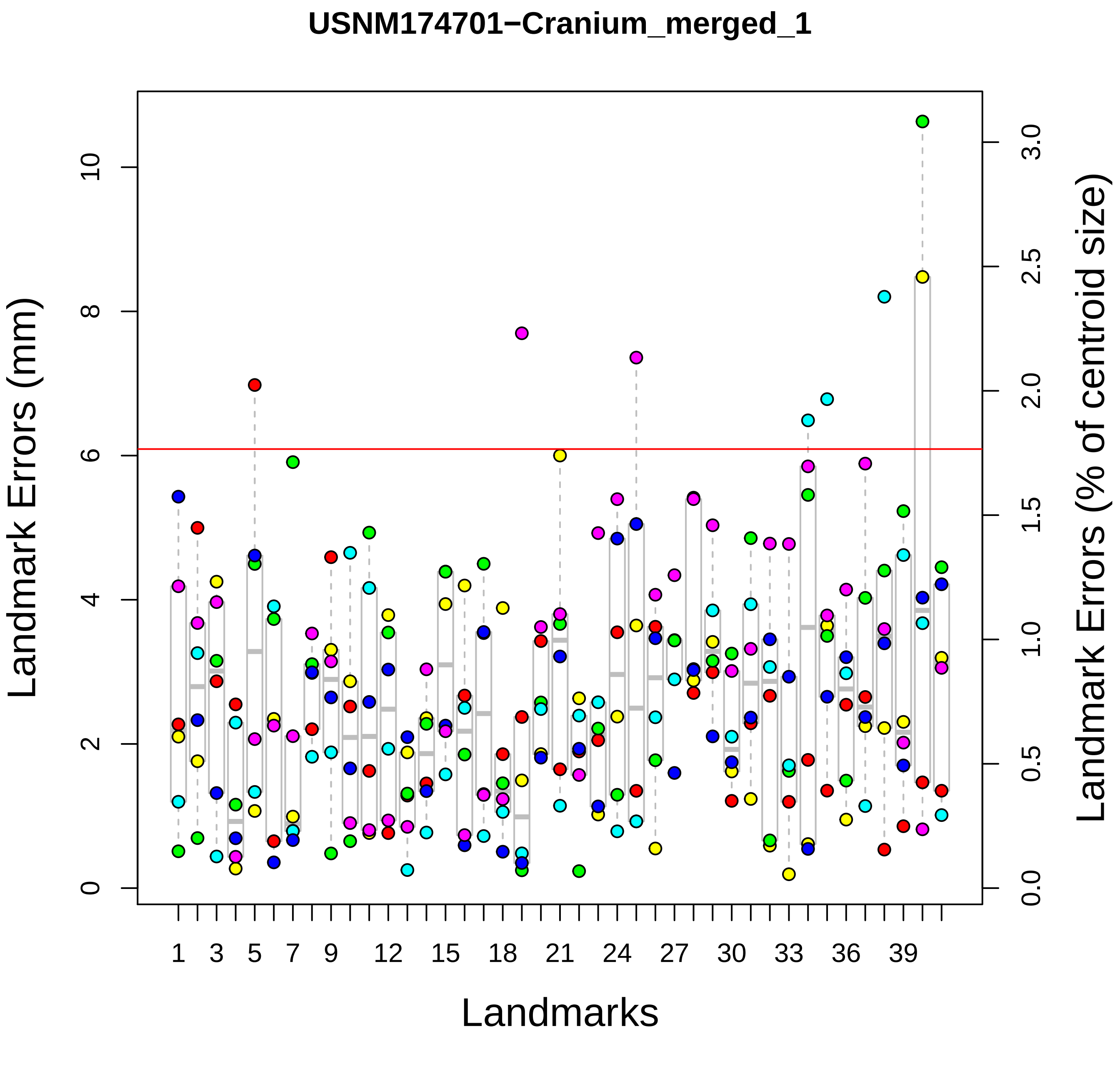
USNM174701−Cranium_merged_1
Landmark Errors (mm)
Landmark Errors (% of centroid size)
Landmarks
0
2
4
6
8
10
0.0
0.5
1.0
1.5
2.0
2.5
3.0
1
3
5
7
9
12
15
18
21
24
27
30
33
36
39

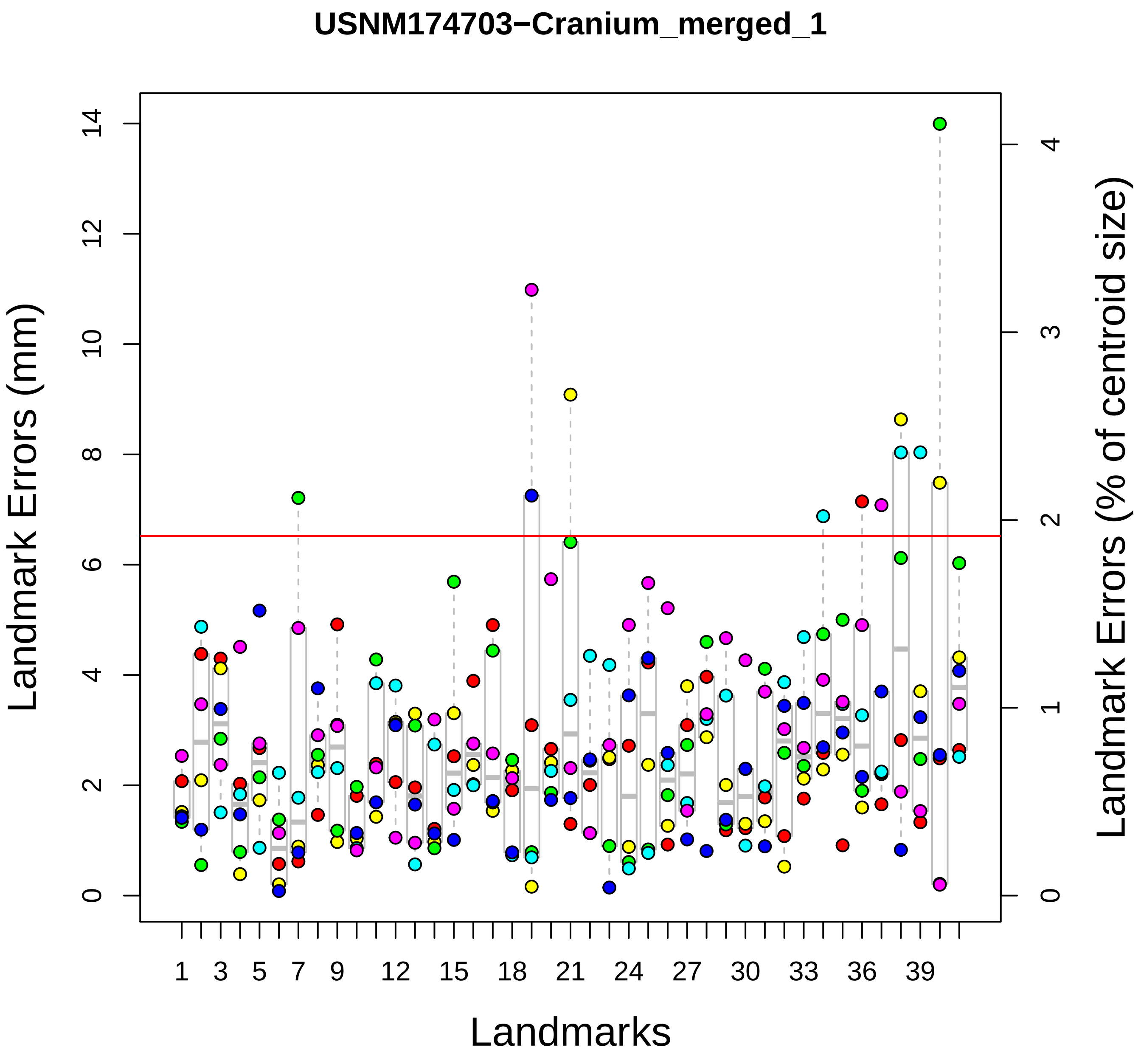
USNM174703−Cranium_merged_1
Landmark Errors (mm)
Landmark Errors (% of centroid size)
Landmarks
0
2
4
6
8
10
12
14
0
1
2
3
4
1
3
5
7
9
12
15
18
21
24
27
30
33
36
39

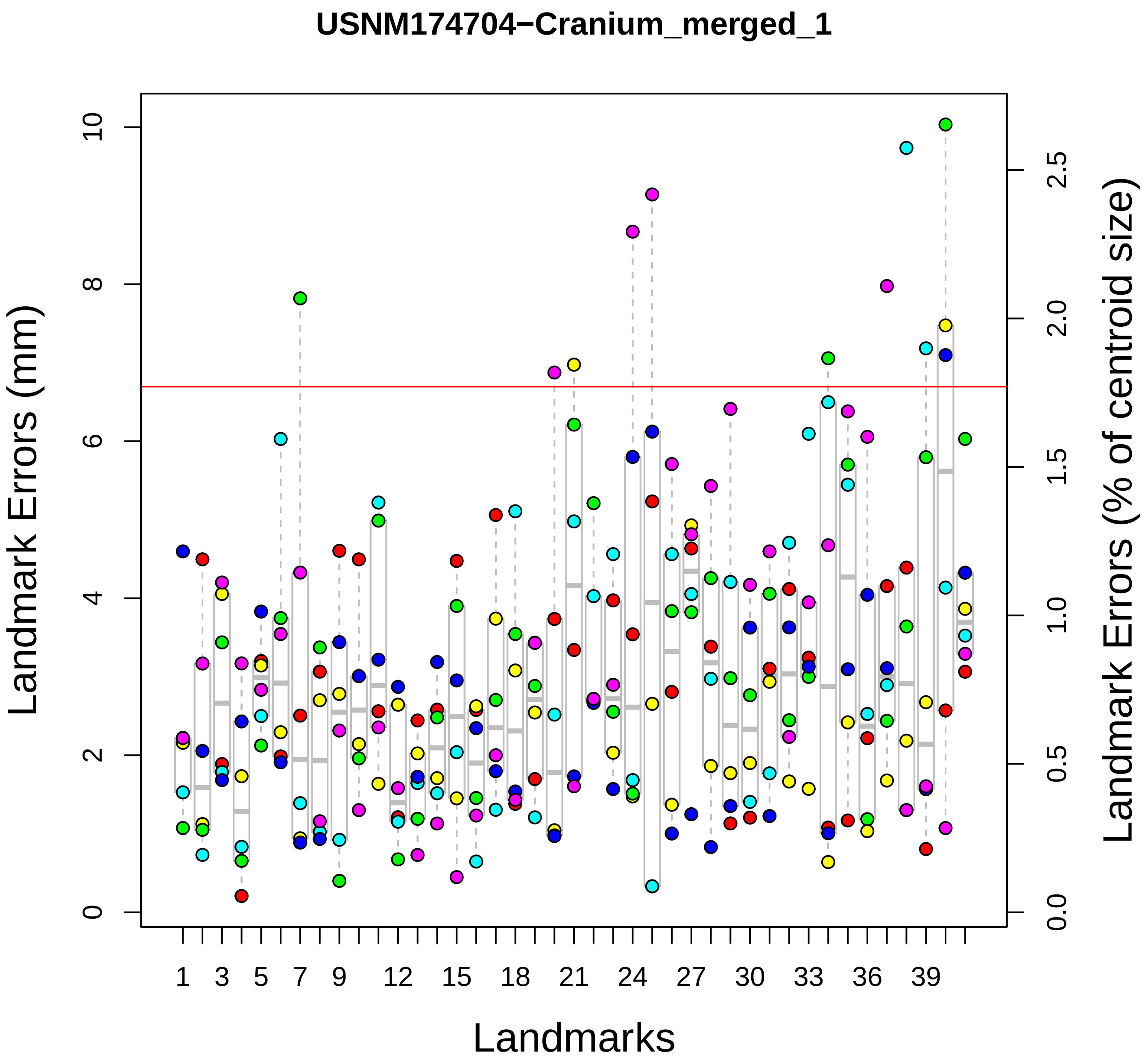
USNM174704−Cranium_merged_1
Landmark Errors (mm)
Landmark Errors (% of centroid size)
Landmarks
0
2
4
6
8
10
0.0
0.5
1.0
1.5
2.0
2.5
1
3
5
7
9
12
15
18
21
24
27
30
33
36
39

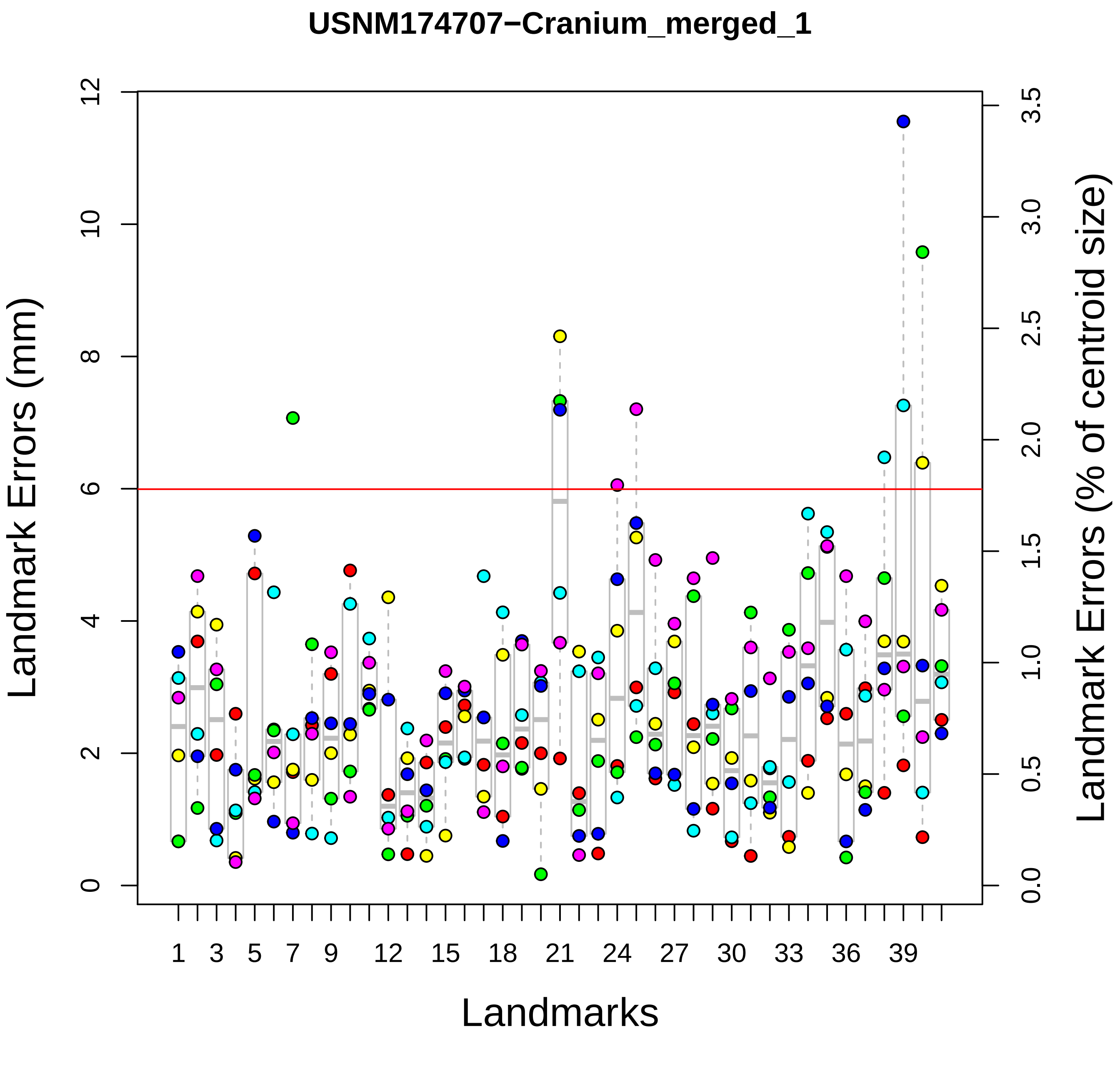
USNM174707−Cranium_merged_1
Landmark Errors (mm)
Landmark Errors (% of centroid size)
Landmarks
0
2
4
6
8
10
12
0.0
0.5
1.0
1.5
2.0
2.5
3.0
3.5
1
3
5
7
9
12
15
18
21
24
27
30
33
36
39

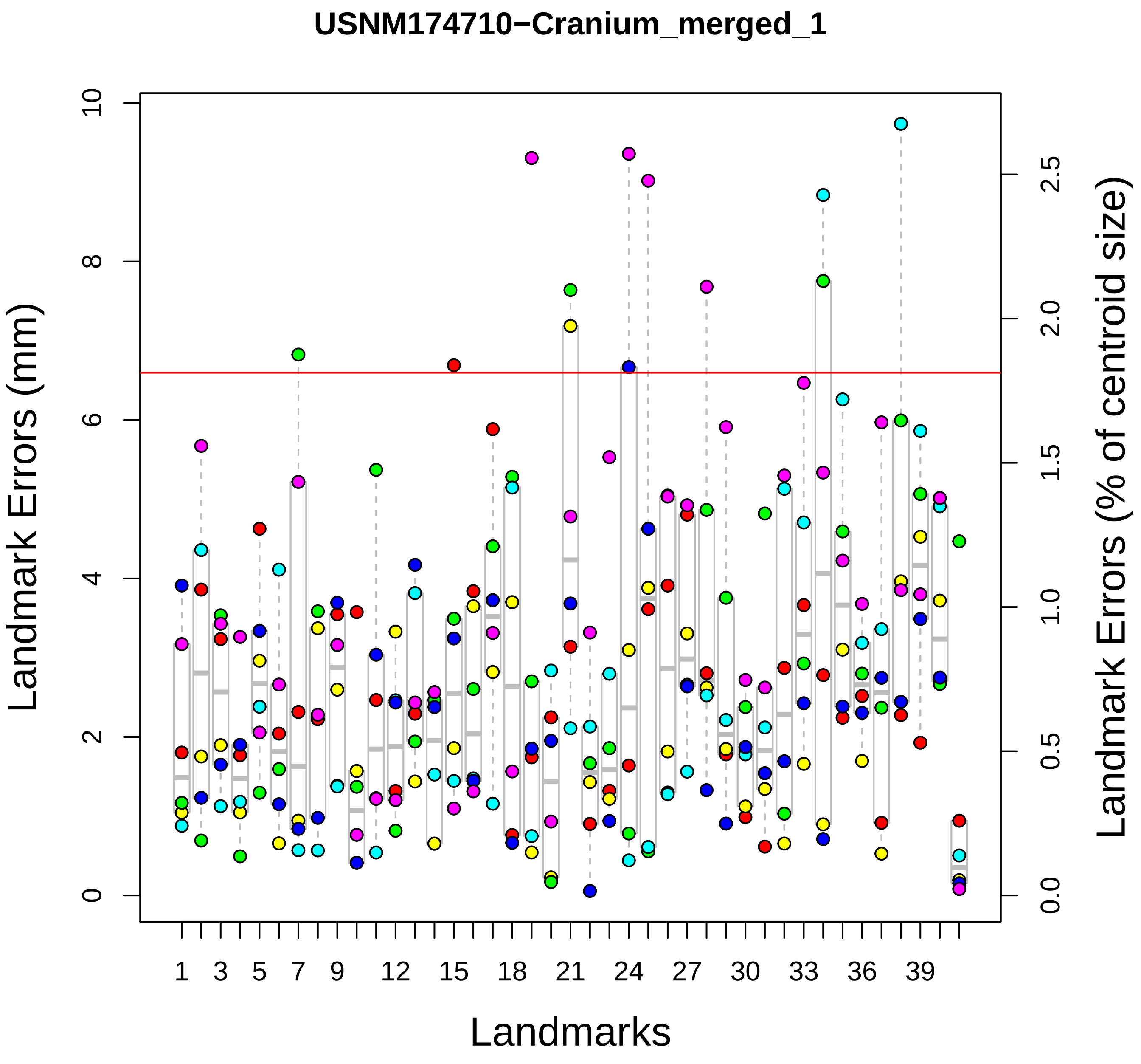

USNM174710−Cranium_merged_1
Landmark Errors (mm)
Landmark Errors (% of centroid size)
Landmarks
0
2
4
6
8
10
0.0
0.5
1.0
1.5
2.0
2.5
1
3
5
7
9
12
15
18
21
24
27
30
33
36
39

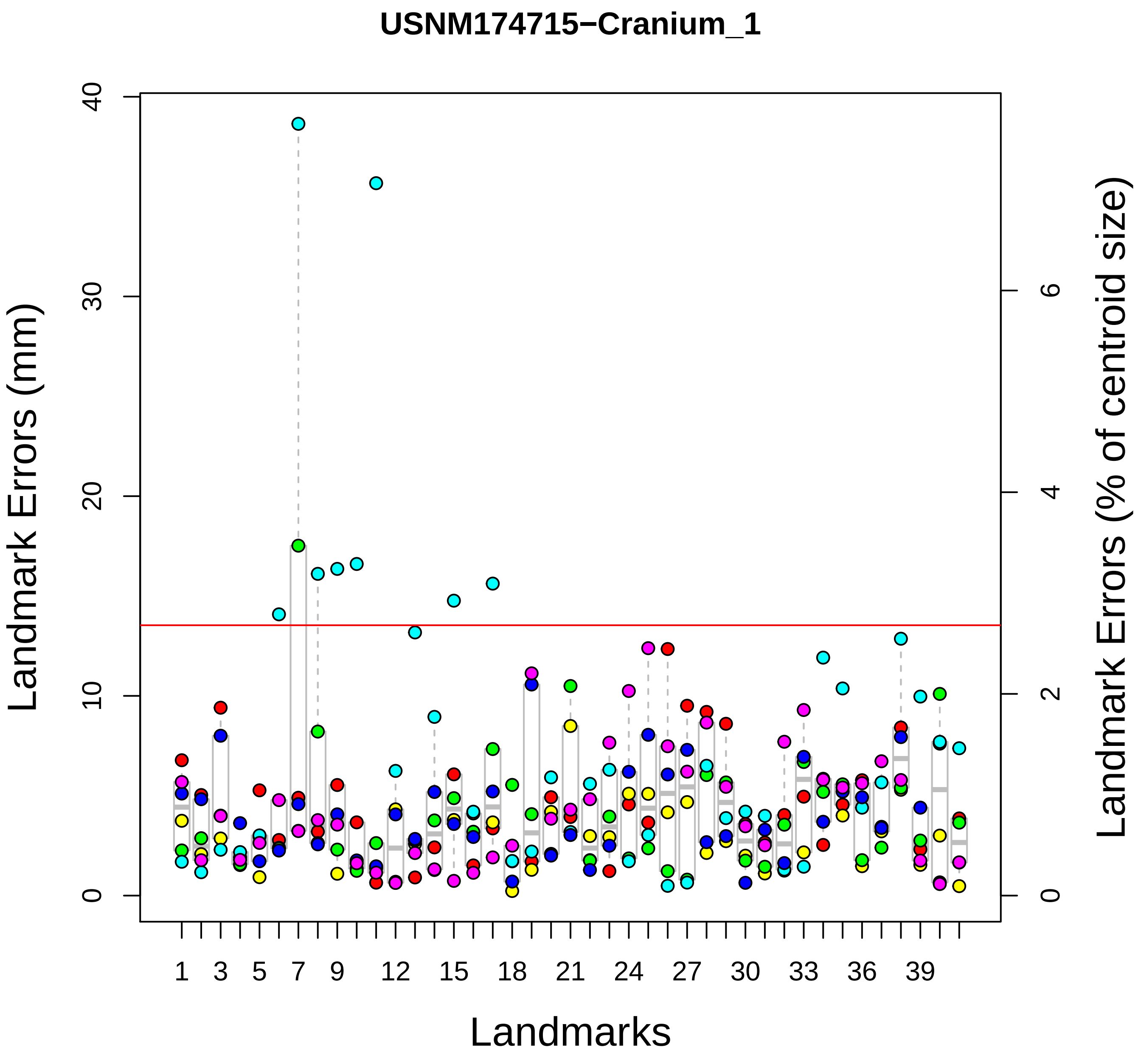

USNM174715−Cranium_1
Landmark Errors (mm)
Landmark Errors (% of centroid size)
Landmarks
0
10
20
30
40
0
2
4
6
1
3
5
7
9
12
15
18
21
24
27
30
33
36
39

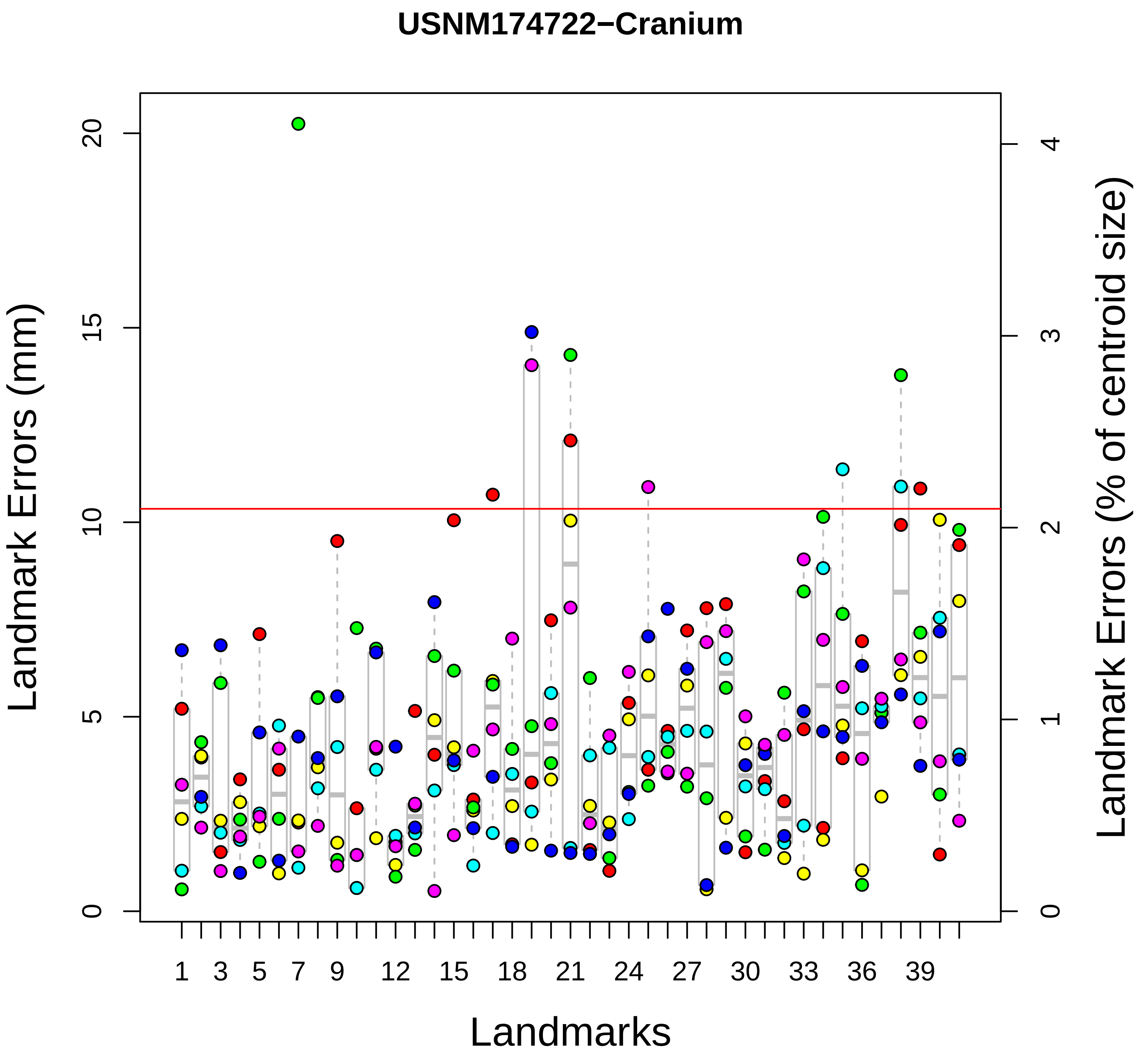

USNM174722−Cranium
Landmark Errors (mm)
Landmark Errors (% of centroid size)
Landmarks
0
5
10
15
20
0
1
2
3
4
1
3
5
7
9
12
15
18
21
24
27
30
33
36
39

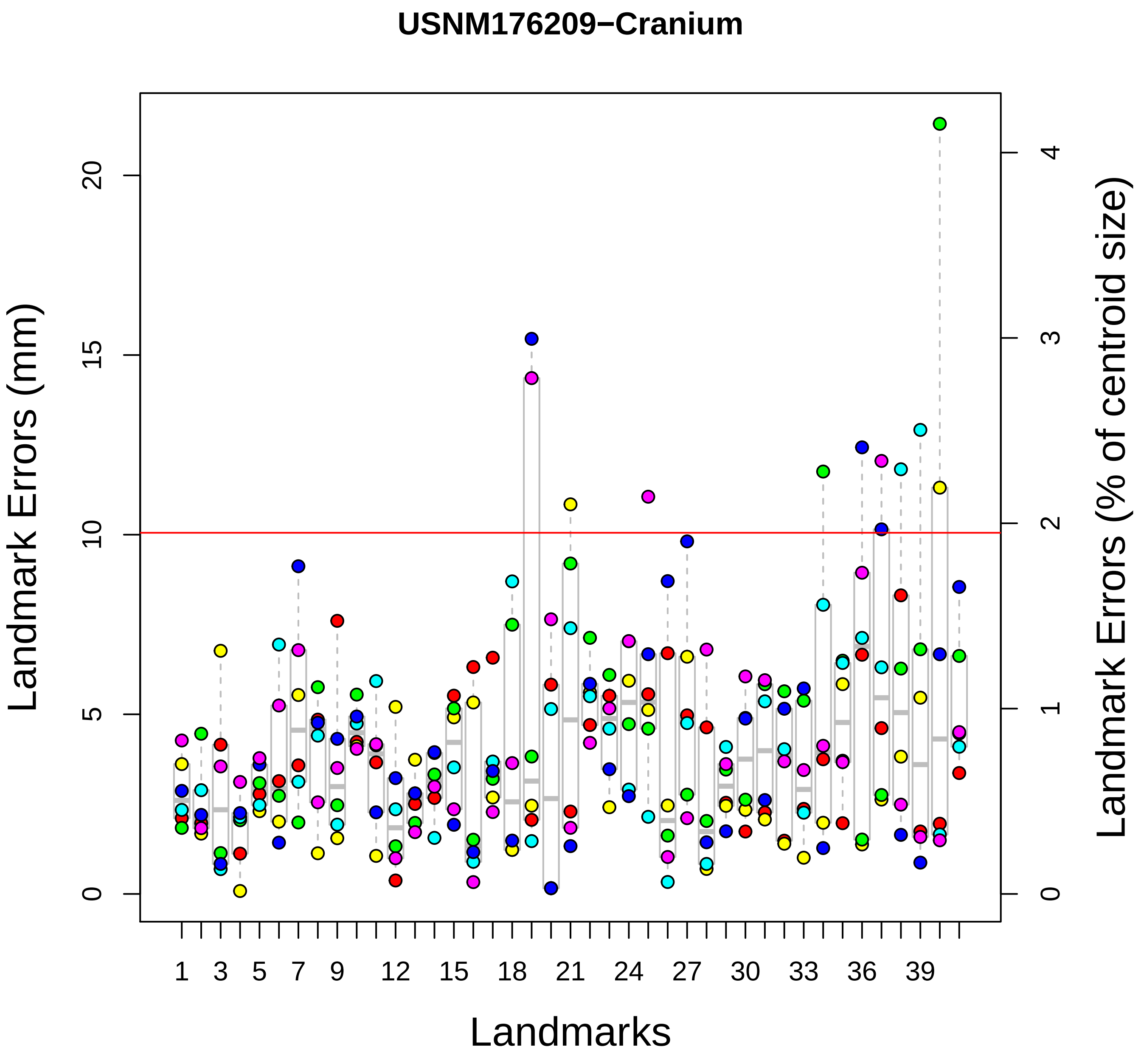

USNM176209−Cranium
Landmark Errors (mm)
Landmark Errors (% of centroid size)
Landmarks
0
5
10
15
20
0
1
2
3
4
1
3
5
7
9
12
15
18
21
24
27
30
33
36
39

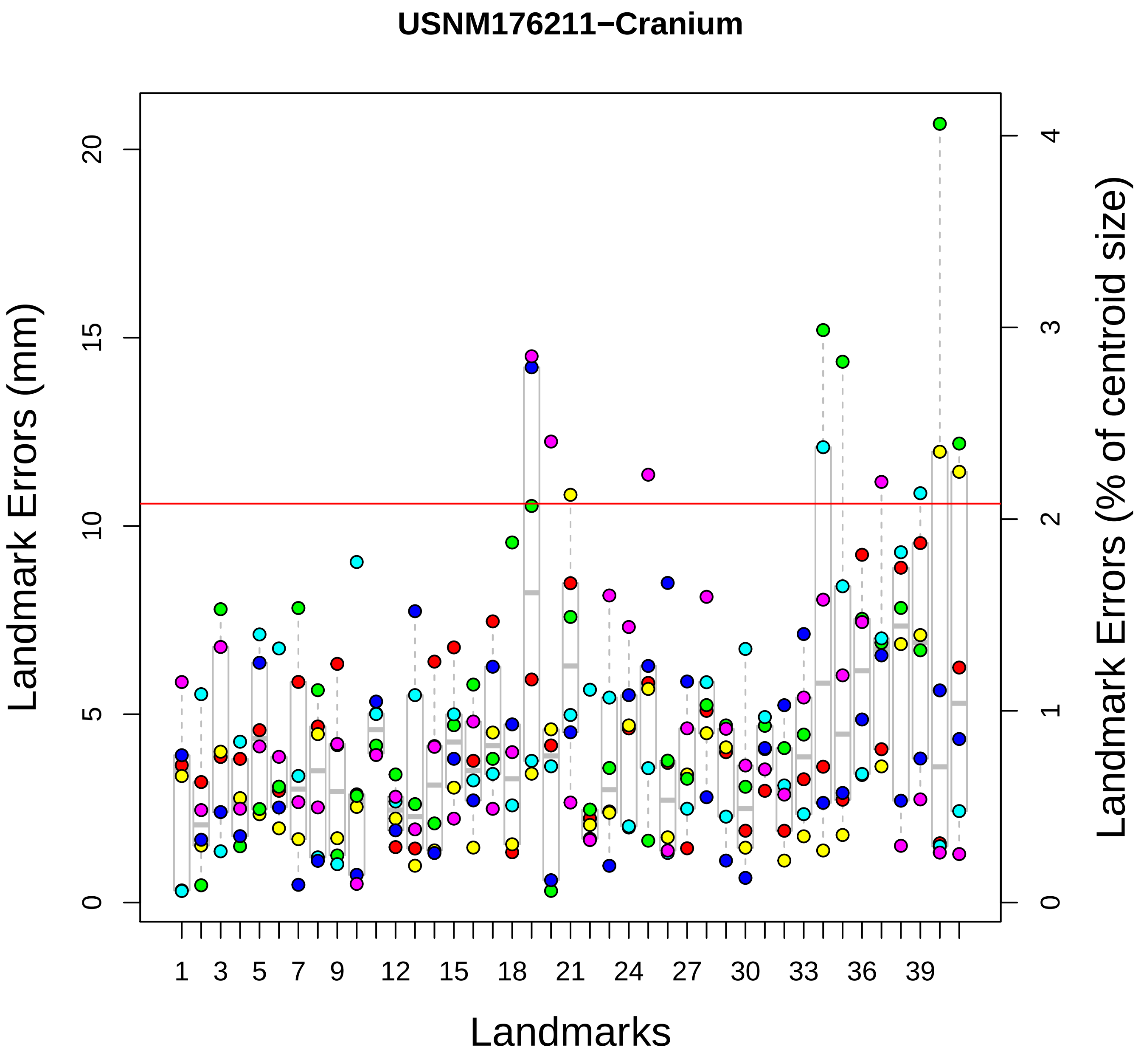
USNM176211−Cranium
Landmark Errors (mm)
Landmark Errors (% of centroid size)
Landmarks
0
5
10
15
20
0
1
2
3
4
1
3
5
7
9
12
15
18
21
24
27
30
33
36
39

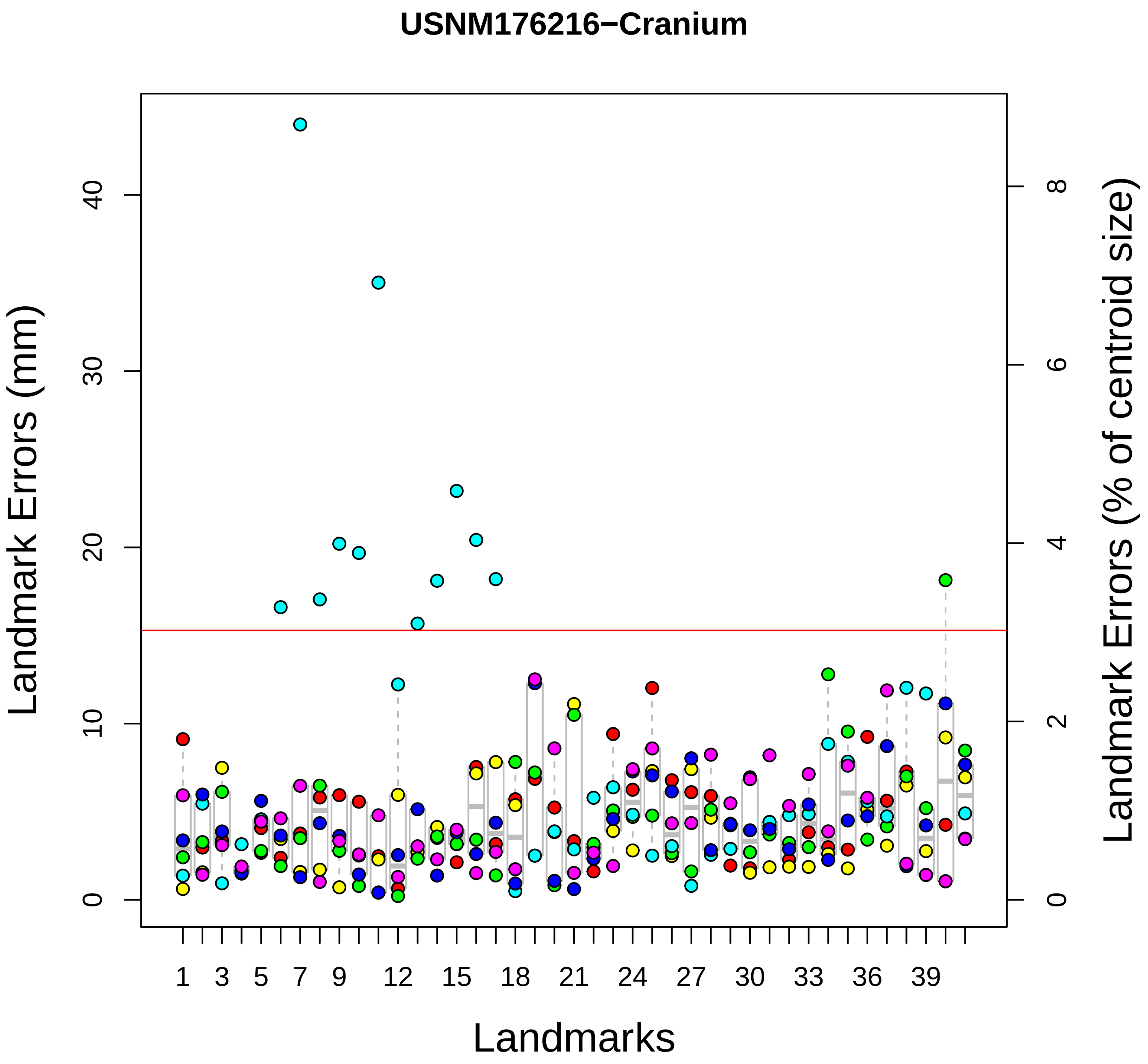
USNM176216−Cranium
Landmark Errors (mm)
Landmark Errors (% of centroid size)
Landmarks
0
10
20
30
40
0
2
4
6
8
1
3
5
7
9
12
15
18
21
24
27
30
33
36
39

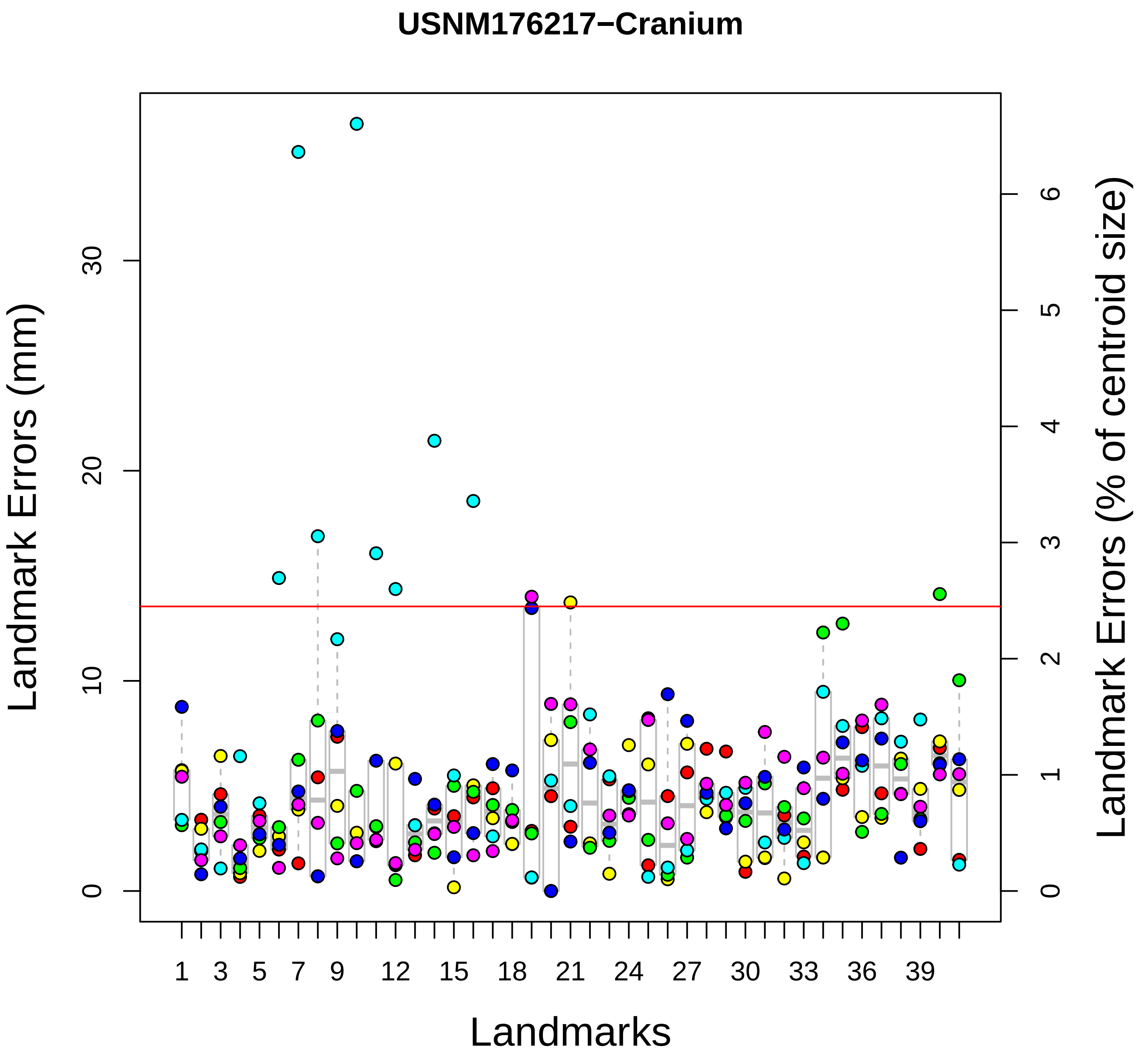

USNM176217−Cranium
Landmark Errors (mm)
Landmark Errors (% of centroid size)
Landmarks
0
10
20
30
0
1
2
3
4
5
6
1
3
5
7
9
12
15
18
21
24
27
30
33
36
39

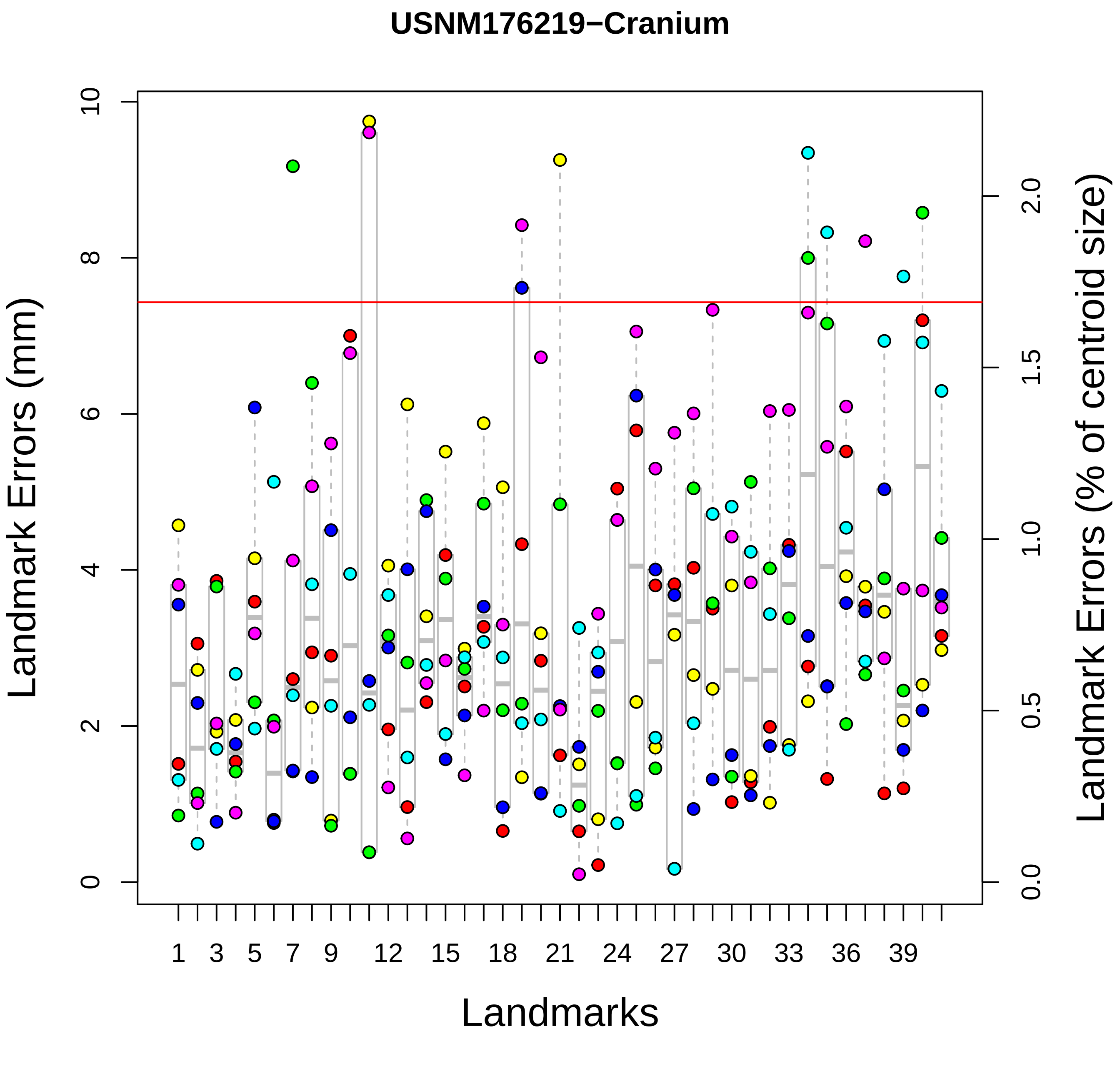
USNM176219−Cranium
Landmark Errors (mm)
Landmark Errors (% of centroid size)
Landmarks
0
2
4
6
8
10
0.0
0.5
1.0
1.5
2.0
1
3
5
7
9
12
15
18
21
24
27
30
33
36
39

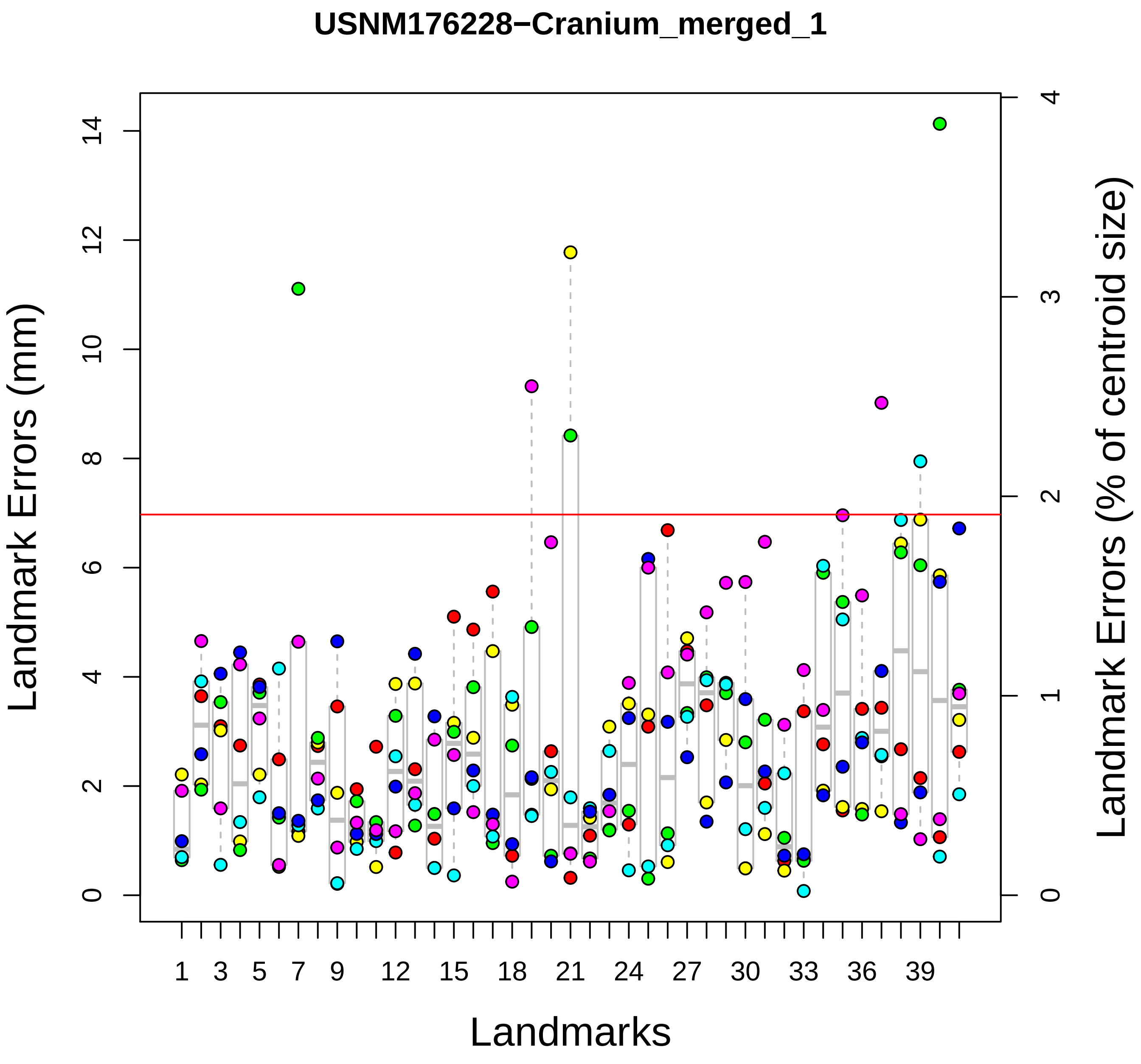
USNM176228−Cranium_merged_1
Landmark Errors (mm)
Landmark Errors (% of centroid size)
Landmarks
0
2
4
6
8
10
12
14
0
1
2
3
4
1
3
5
7
9
12
15
18
21
24
27
30
33
36
39

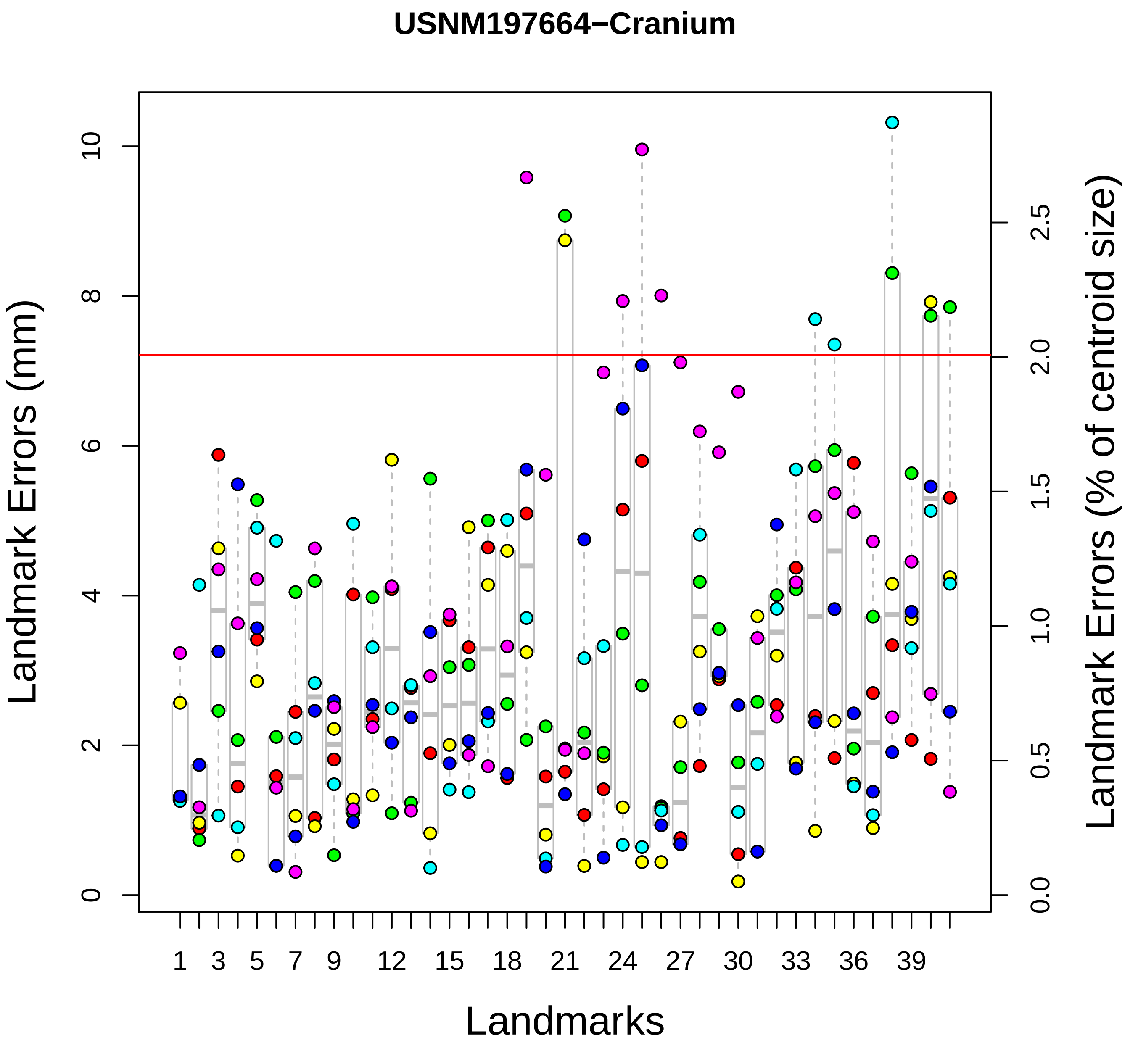

USNM197664−Cranium
Landmark Errors (mm)
Landmark Errors (% of centroid size)
Landmarks
0
2
4
6
8
10
0.0
0.5
1.0
1.5
2.0
2.5
1
3
5
7
9
12
15
18
21
24
27
30
33
36
39

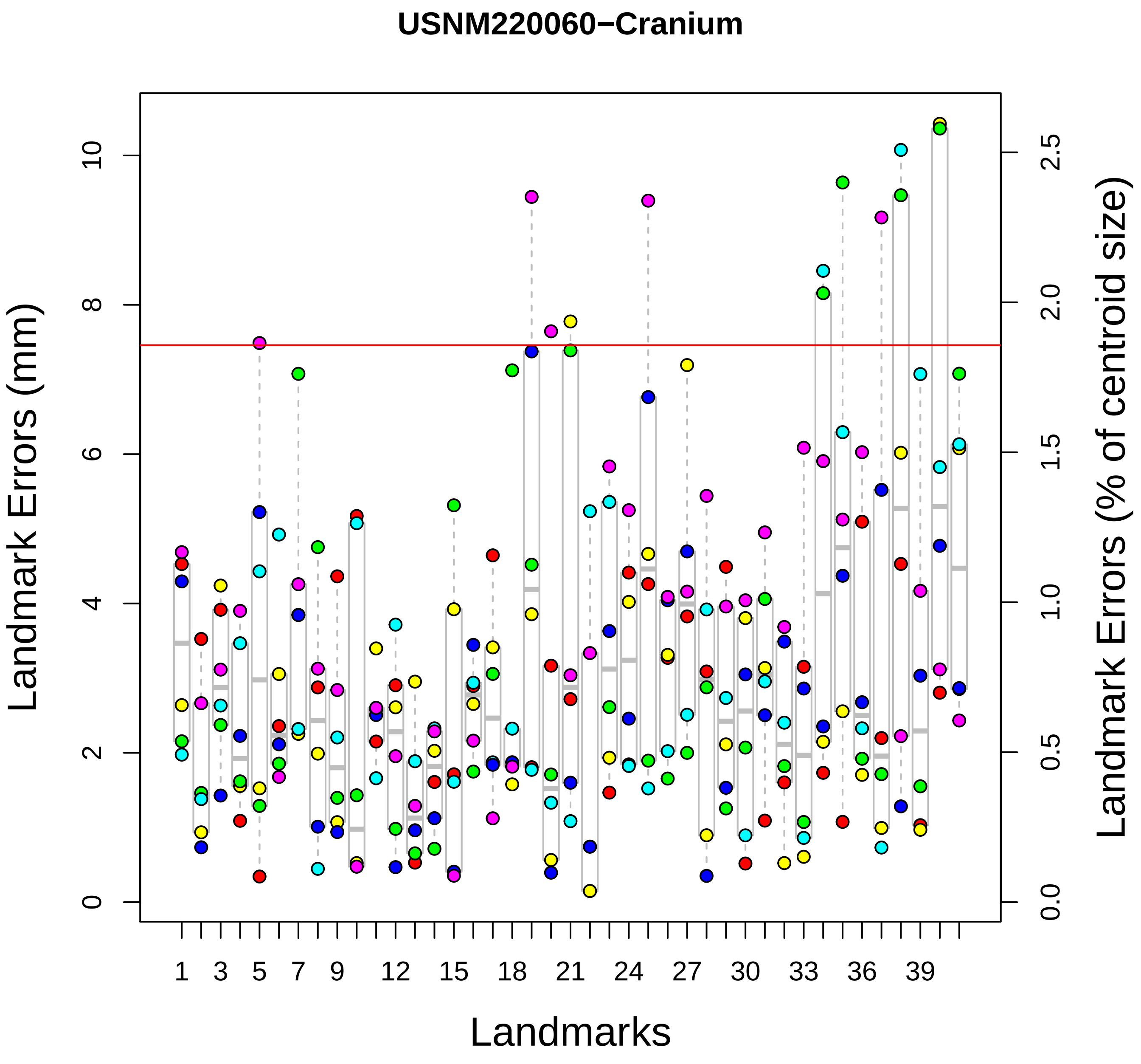

USNM220060−Cranium
Landmark Errors (mm)
Landmark Errors (% of centroid size)
Landmarks
0
2
4
6
8
10
0.0
0.5
1.0
1.5
2.0
2.5
1 3 5 7 9 12 15 18 21 24 27 30 33 36 39

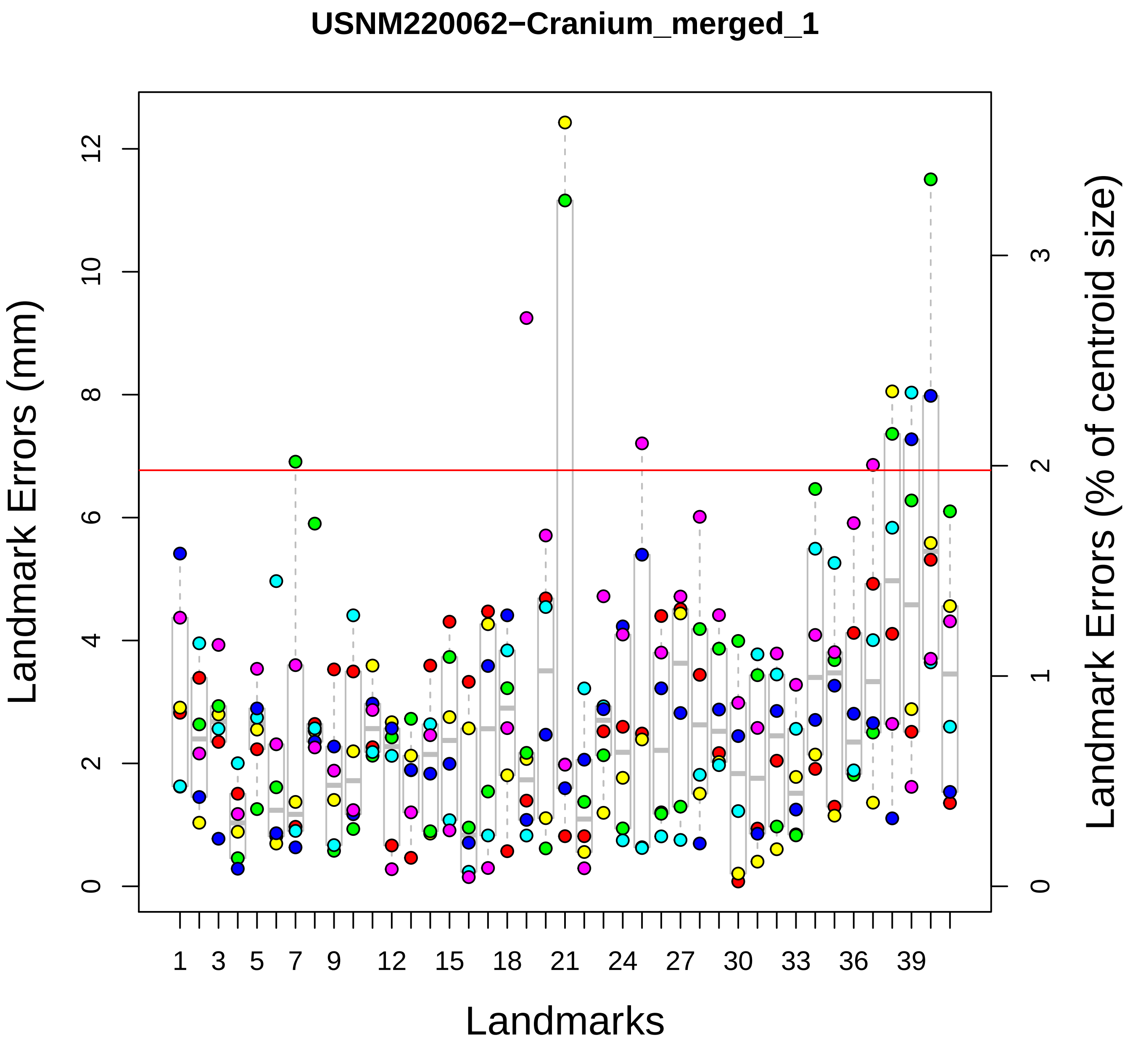

USNM220062−Cranium_merged_1
Landmark Errors (mm)
Landmark Errors (% of centroid size)
Landmarks
0
2
4
6
8
10
12
0
1
2
3
1
3
5
7
9
12
15
18
21
24
27
30
33
36
39

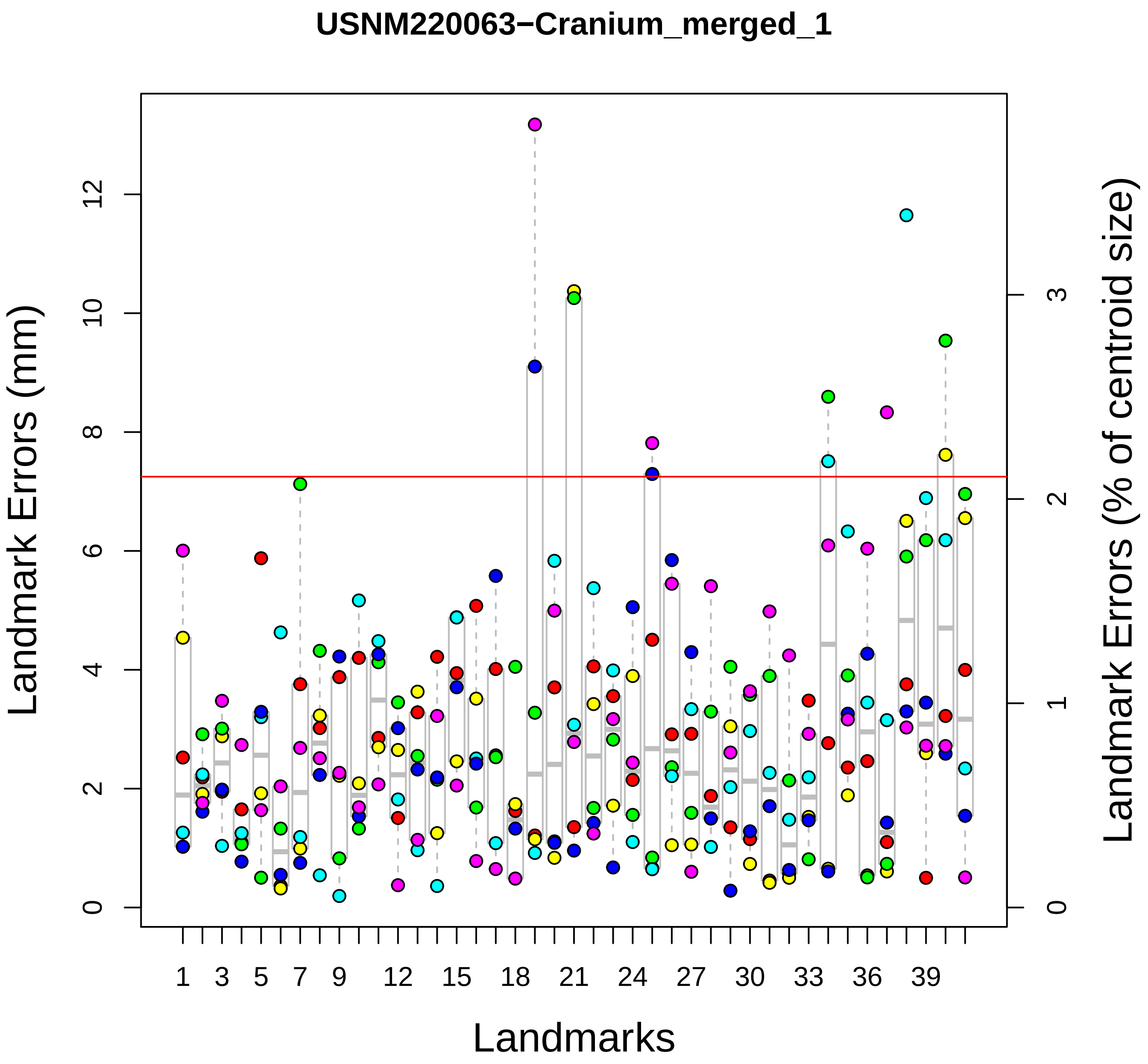

USNM220063−Cranium_merged_1
Landmark Errors (mm)
Landmark Errors (% of centroid size)
Landmarks
0
2
4
6
8
10
12
0
1
2
3
1
3
5
7
9
12
15
18
21
24
27
30
33
36
39

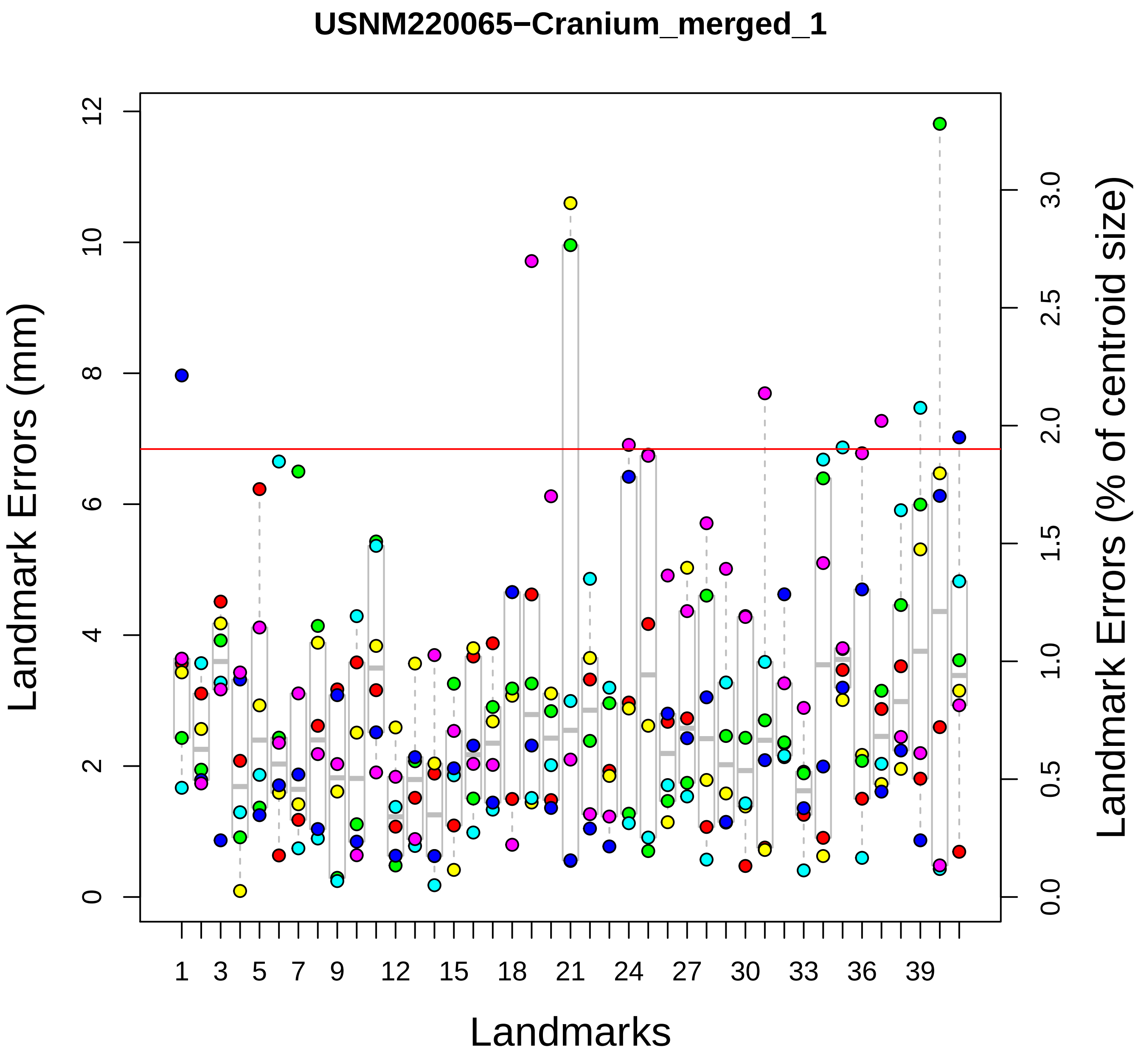
USNM220065−Cranium_merged_1
Landmark Errors (mm)
Landmark Errors (% of centroid size)
Landmarks
0
2
4
6
8
10
12
0.0
0.5
1.0
1.5
2.0
2.5
3.0
1
3
5
7
9
12
15
18
21
24
27
30
33
36
39

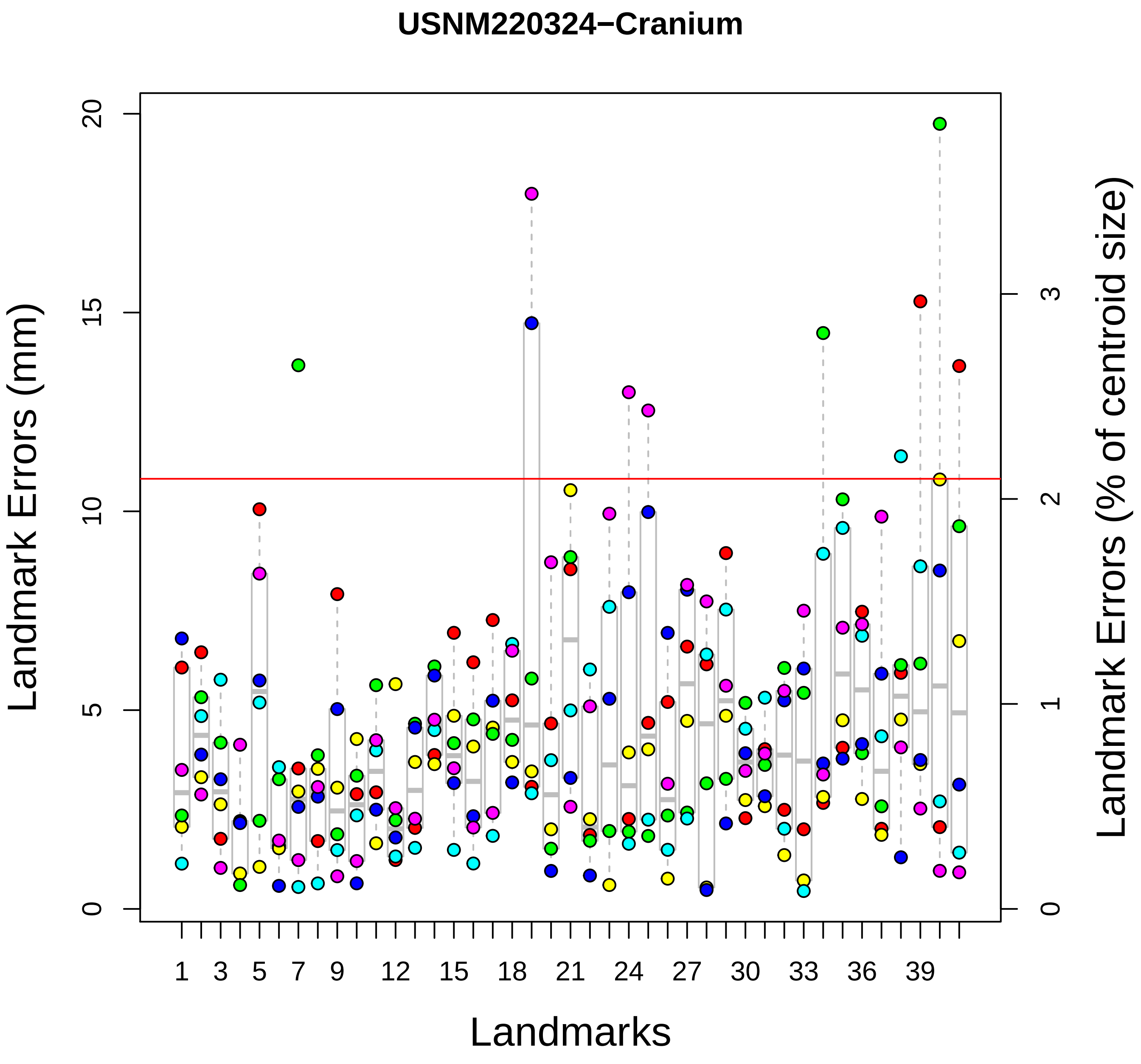
USNM220324−Cranium
Landmark Errors (mm)
Landmark Errors (% of centroid size)
Landmarks
0
5
10
15
20
0
1
2
3
1
3
5
7
9
12
15
18
21
24
27
30
33
36
39

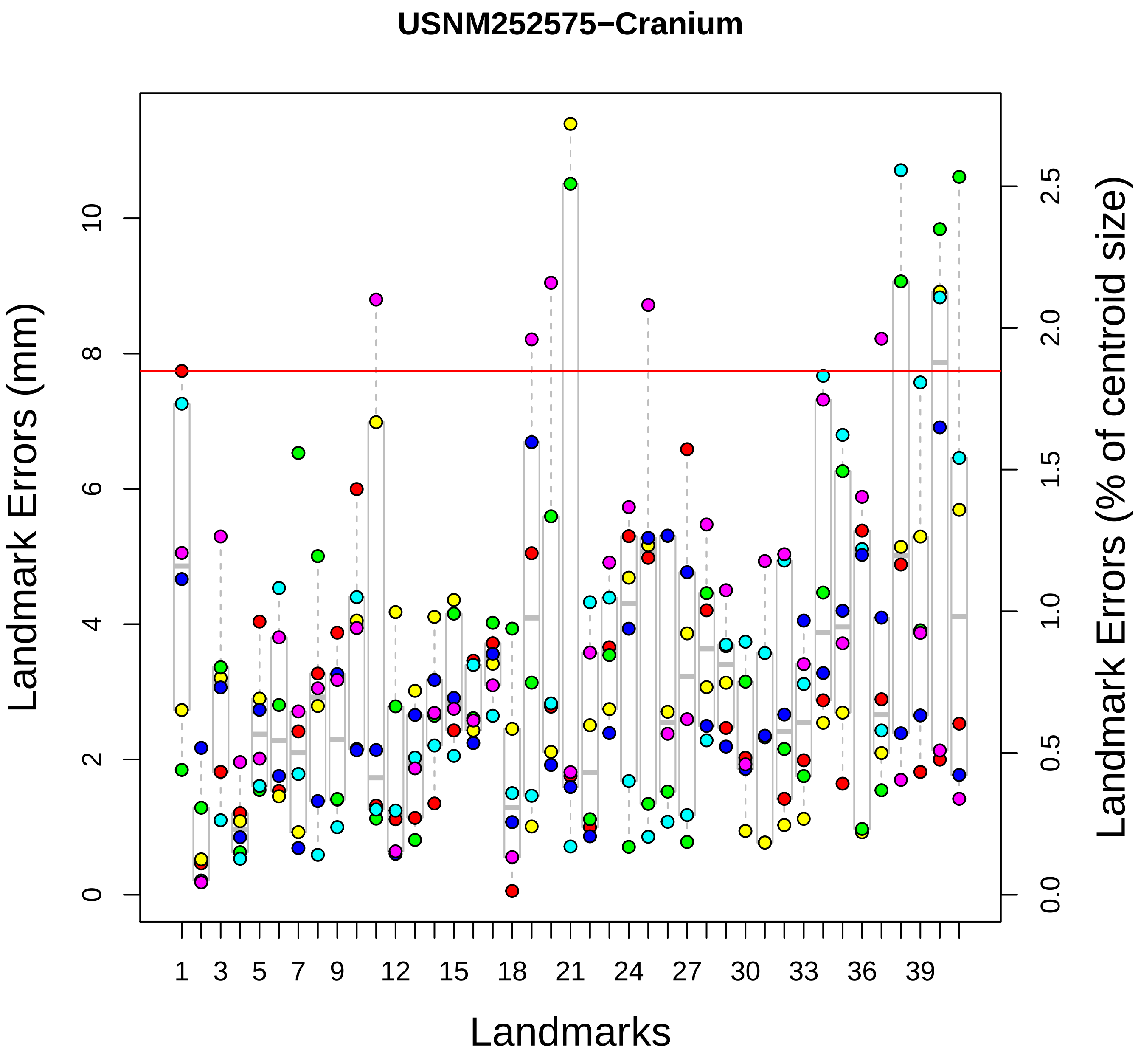
USNM252575−Cranium
Landmark Errors (mm)
Landmark Errors (% of centroid size)
Landmarks
0
2
4
6
8
10
0.0
0.5
1.0
1.5
2.0
2.5
1
3
5
7
9
12
15
18
21
24
27
30
33
36
39

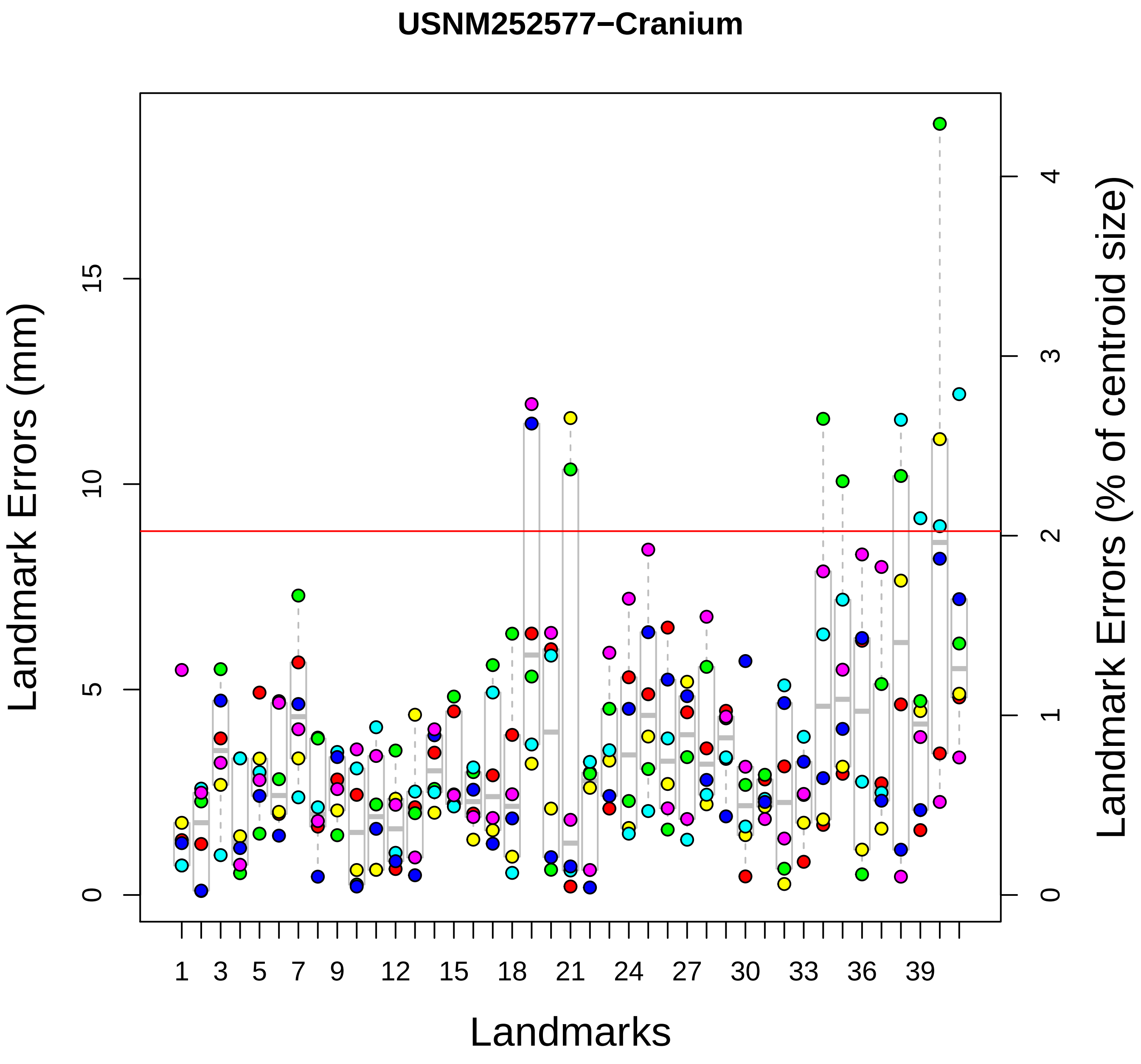
USNM252577−Cranium
Landmark Errors (mm)
Landmark Errors (% of centroid size)
Landmarks
0
5
10
15
0
1
2
3
4
1
3
5
7
9
12
15
18
21
24
27
30
33
36
39

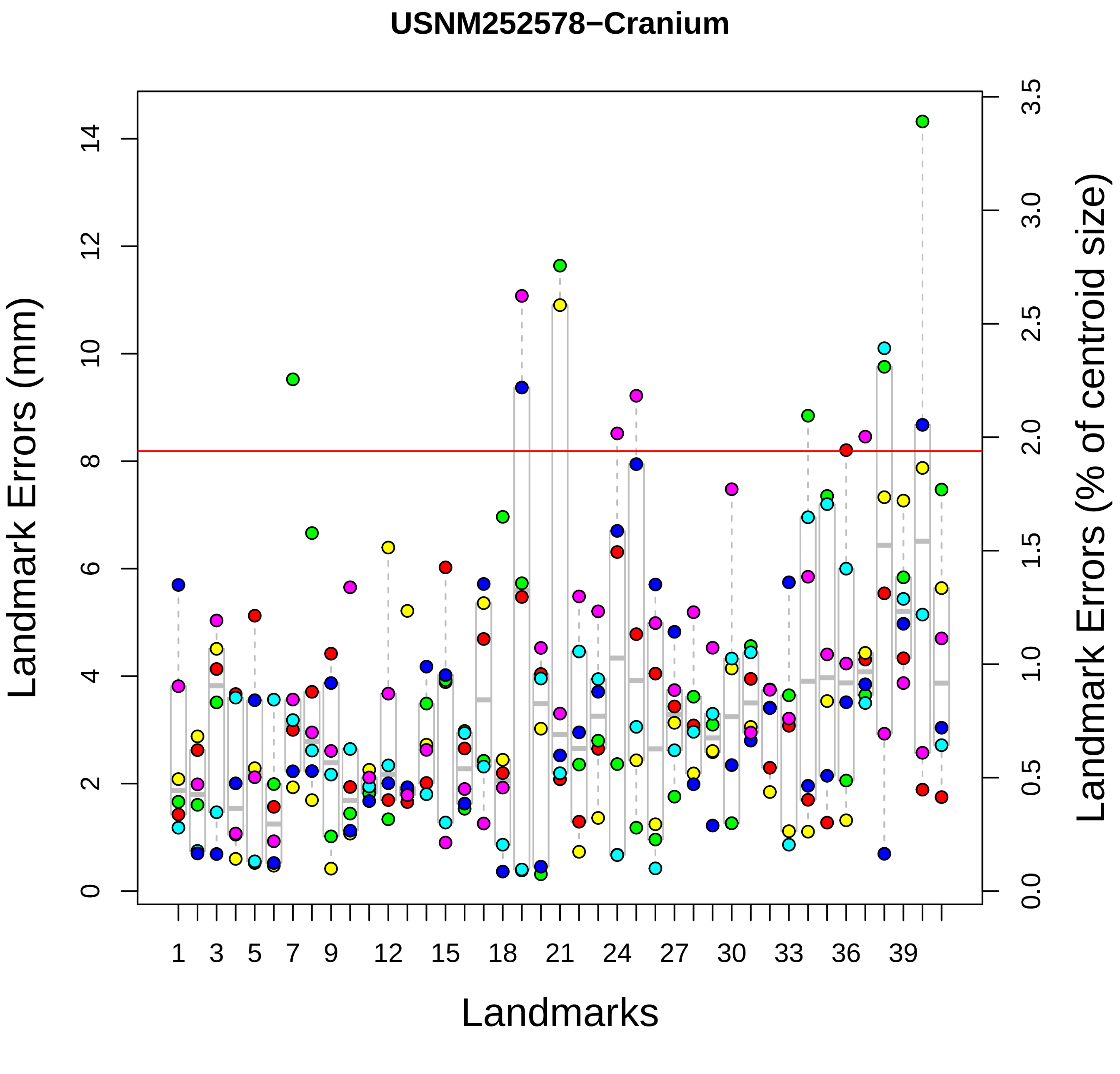
USNM252578−Cranium
Landmark Errors (mm)
Landmark Errors (% of centroid size)
Landmarks
0
2
4
6
8
10
12
14
0.0
0.5
1.0
1.5
2.0
2.5
3.0
3.5
1 3 5 7 9 12 15 18 21 24 27 30 33 36 39

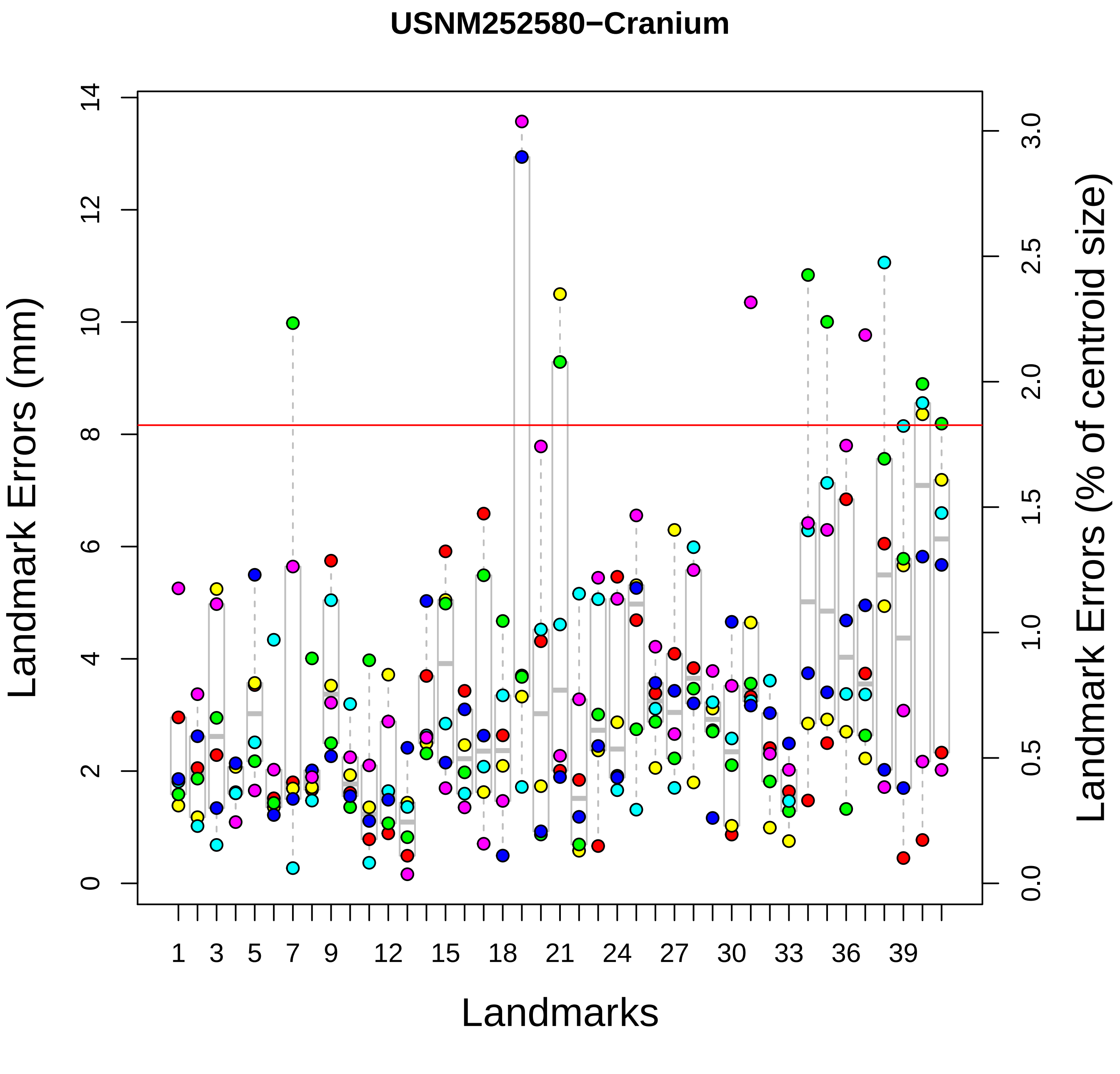
USNM252580−Cranium
Landmark Errors (mm)
Landmark Errors (% of centroid size)
Landmarks
0
2
4
6
8
10
12
14
0.0
0.5
1.0
1.5
2.0
2.5
3.0
1
3
5
7
9
12
15
18
21
24
27
30
33
36
39

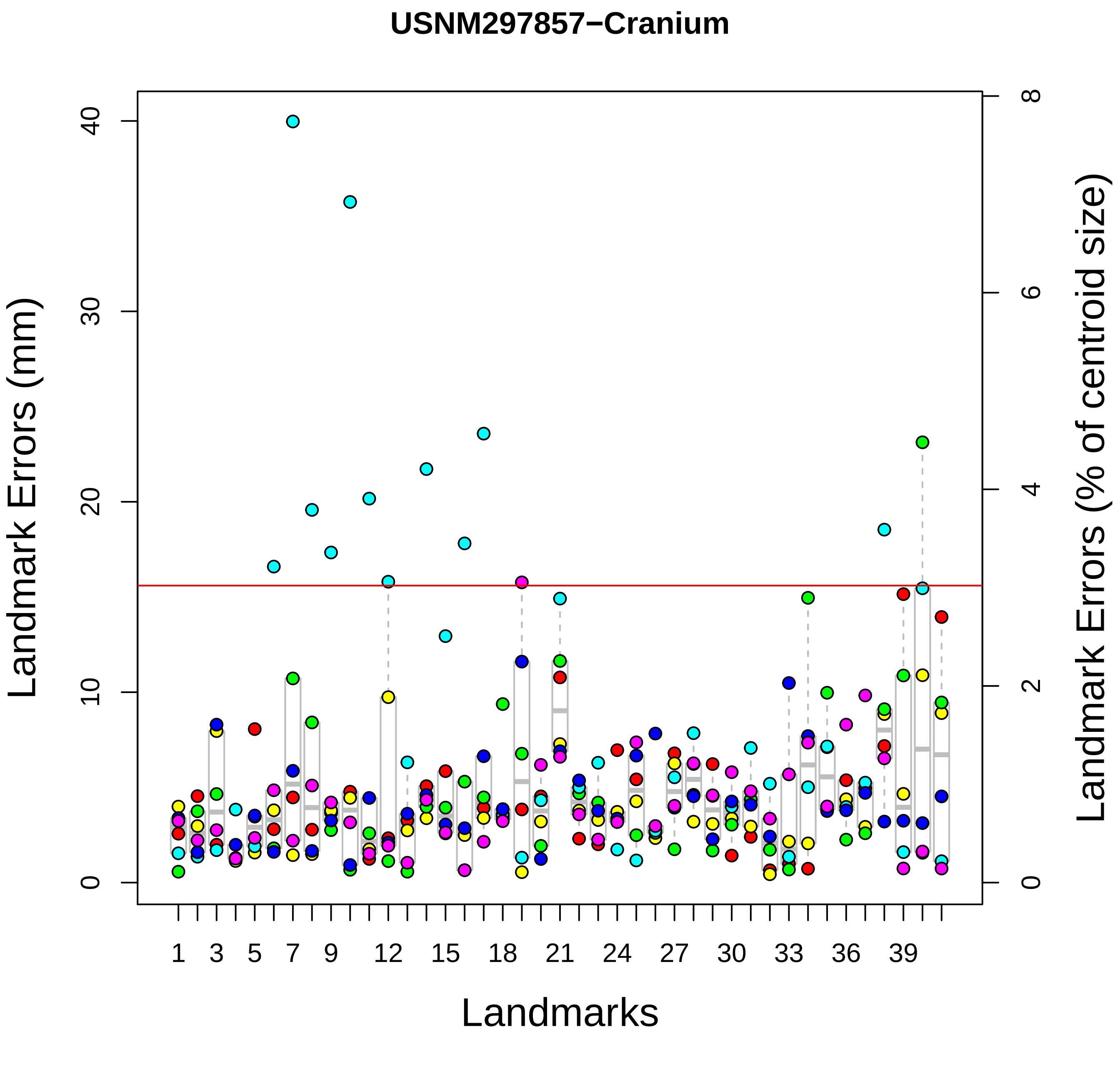
USNM297857−Cranium
Landmark Errors (mm)
Landmark Errors (% of centroid size)
Landmarks
0
10
20
30
40
0
2
4
6
8
1
3
5
7
9
12
15
18
21
24
27
30
33
36
39

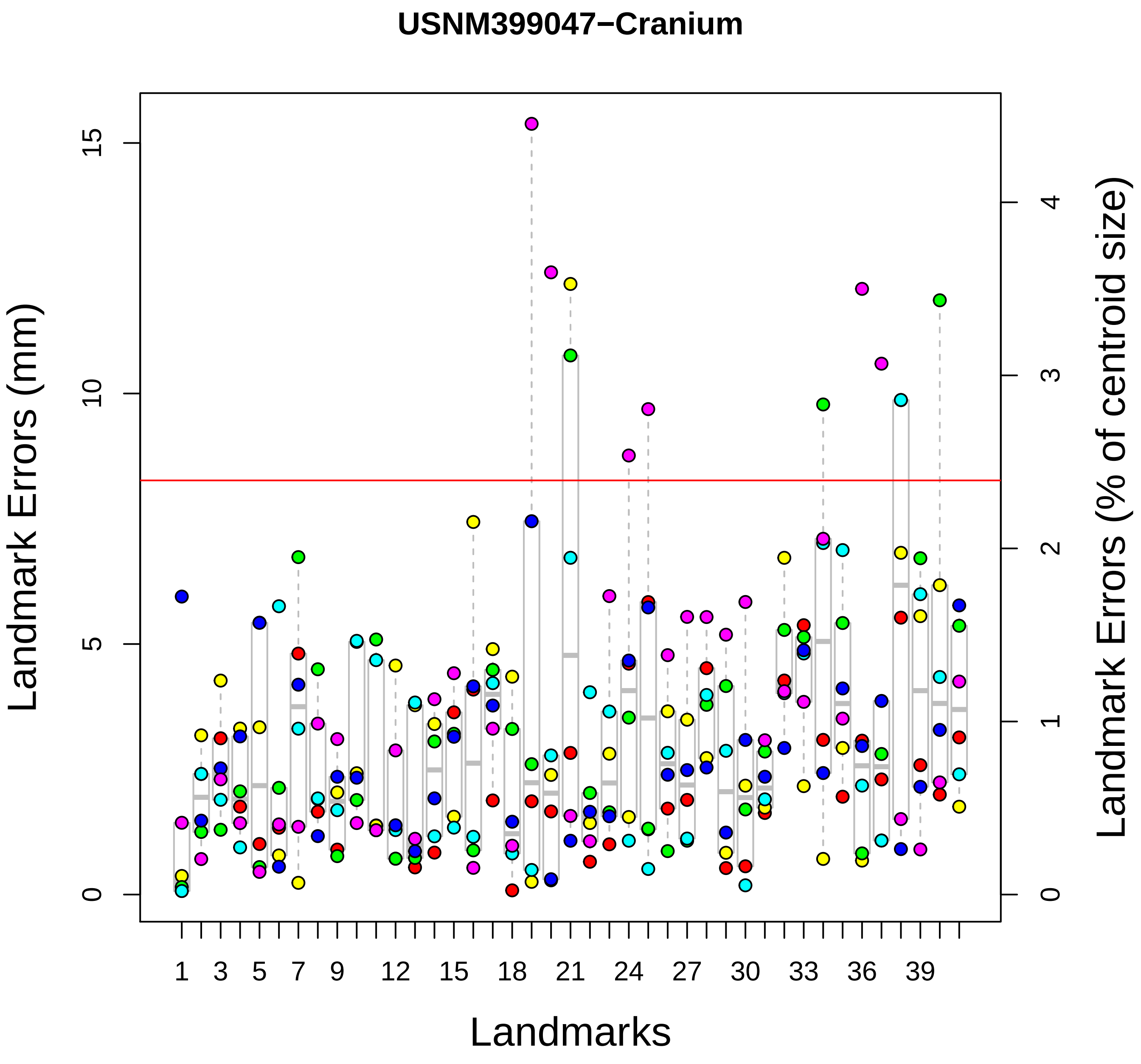
USNM399047−Cranium
Landmark Errors (mm)
Landmark Errors (% of centroid size)
Landmarks
0
5
10
15
0
1
2
3
4
1
3
5
7
9
12
15
18
21
24
27
30
33
36
39

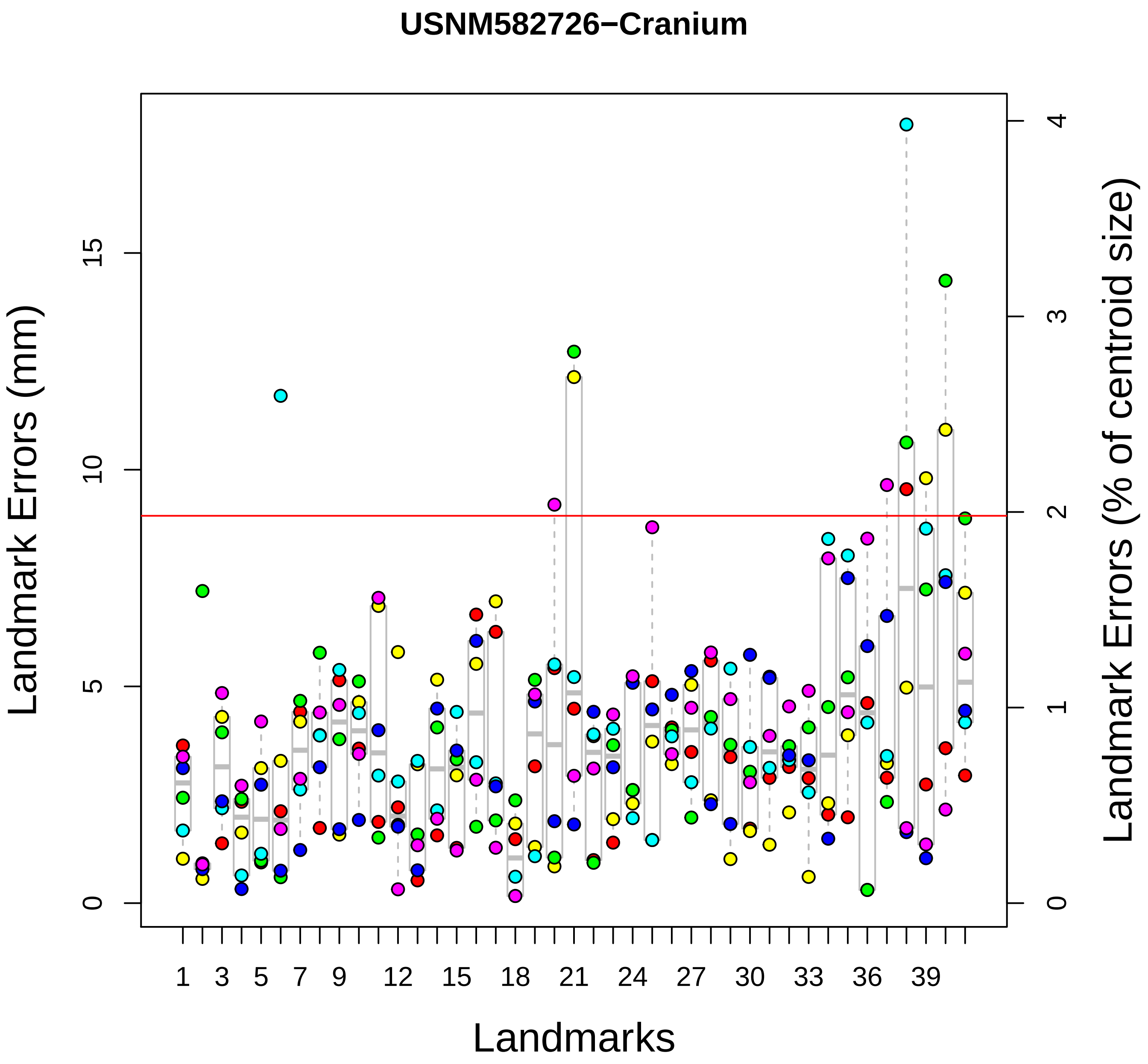
USNM582726−Cranium
Landmark Errors (mm)
Landmark Errors (% of centroid size)
Landmarks
0
5
10
15
0
1
2
3
4
1
3
5
7
9
12
15
18
21
24
27
30
33
36
39

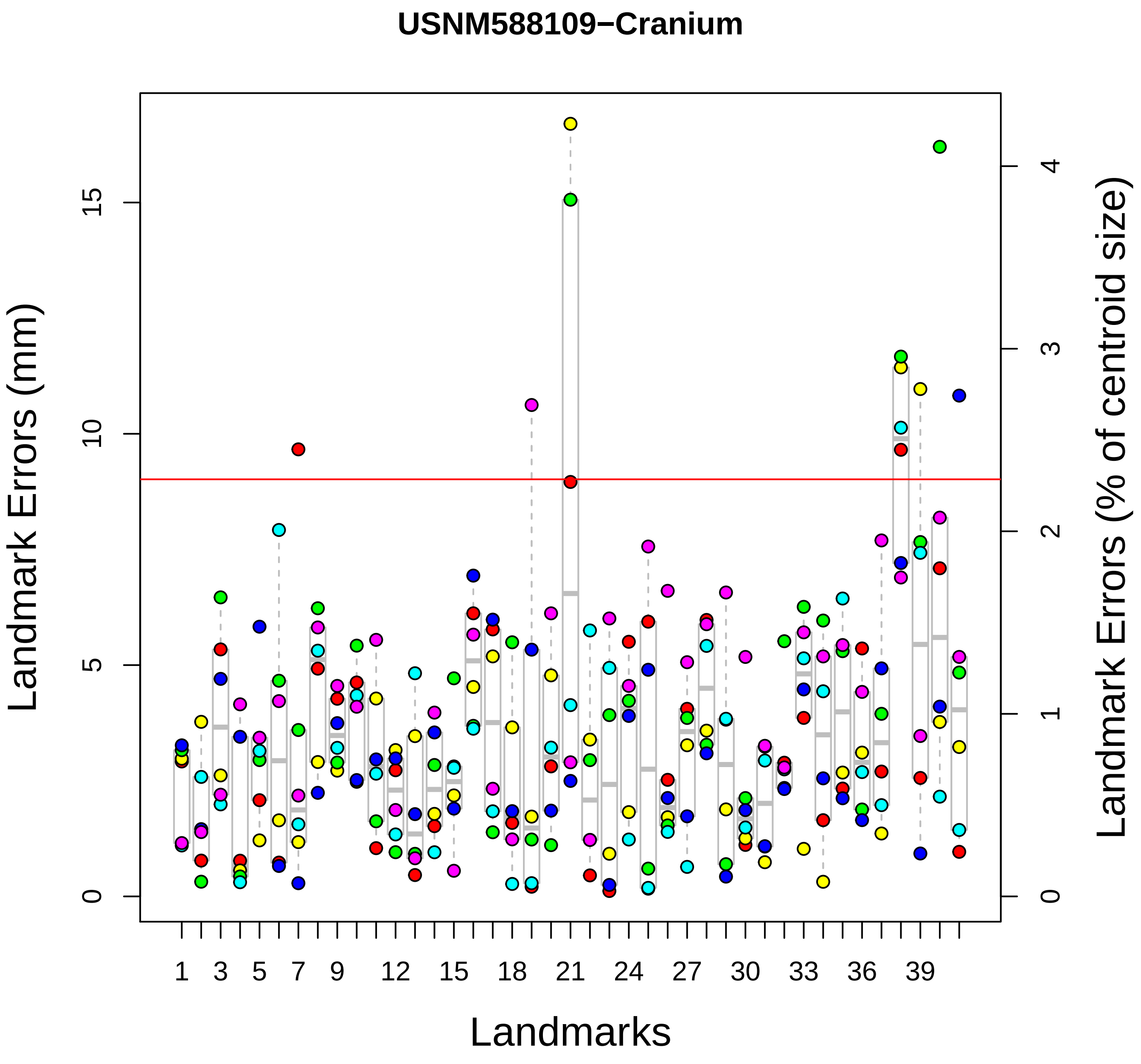

USNM588109−Cranium
Landmark Errors (mm)
Landmark Errors (% of centroid size)
Landmarks
0
5
10
15
0
1
2
3
4
1
3
5
7
9
12
15
18
21
24
27
30
33
36
39

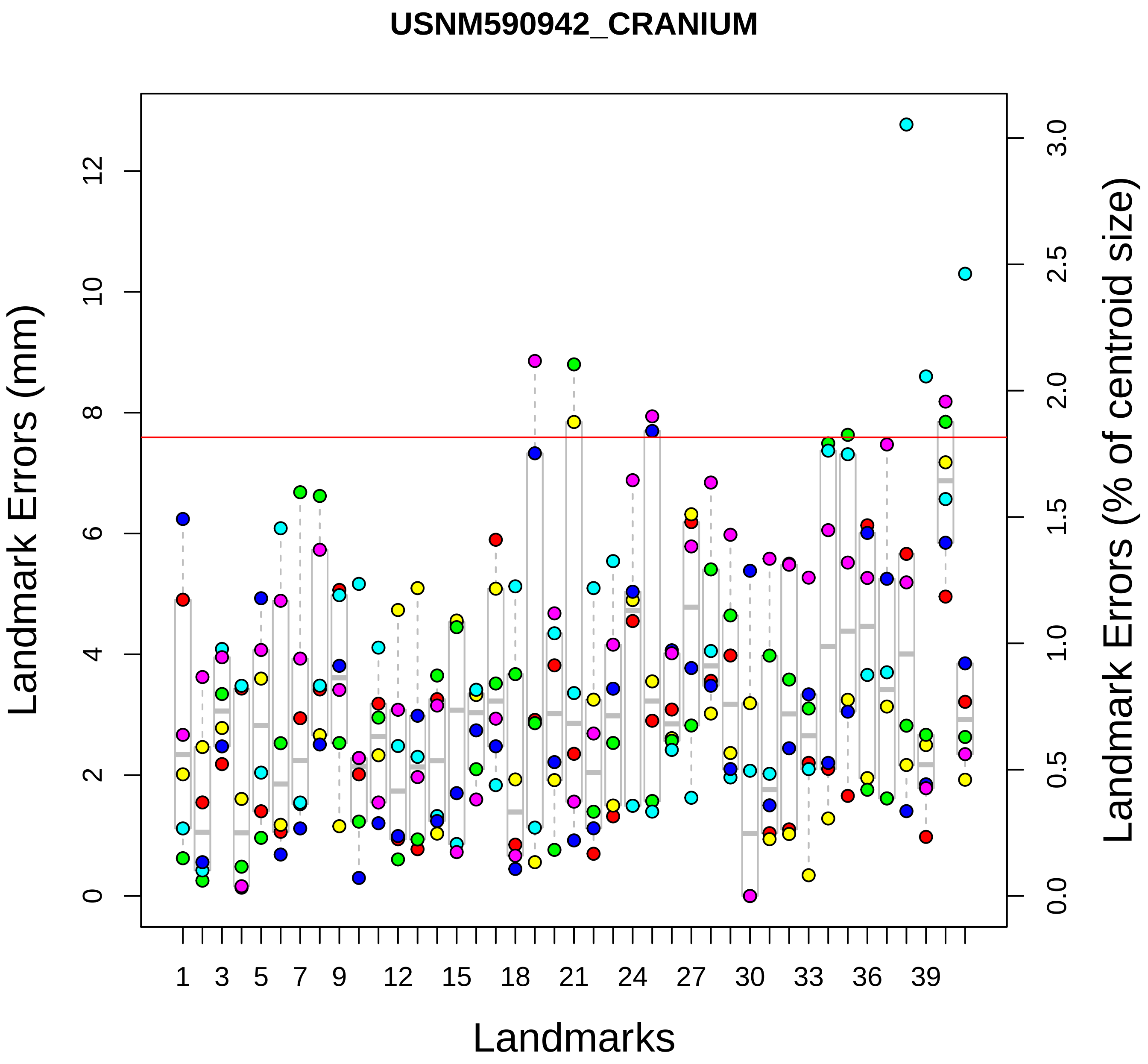

USNM590942_CRANIUM
Landmark Errors (mm)
Landmark Errors (% of centroid size)
Landmarks
0
2
4
6
8
10
12
0.0
0.5
1.0
1.5
2.0
2.5
3.0
1
3
5
7
9
12
15
18
21
24
27
30
33
36
39

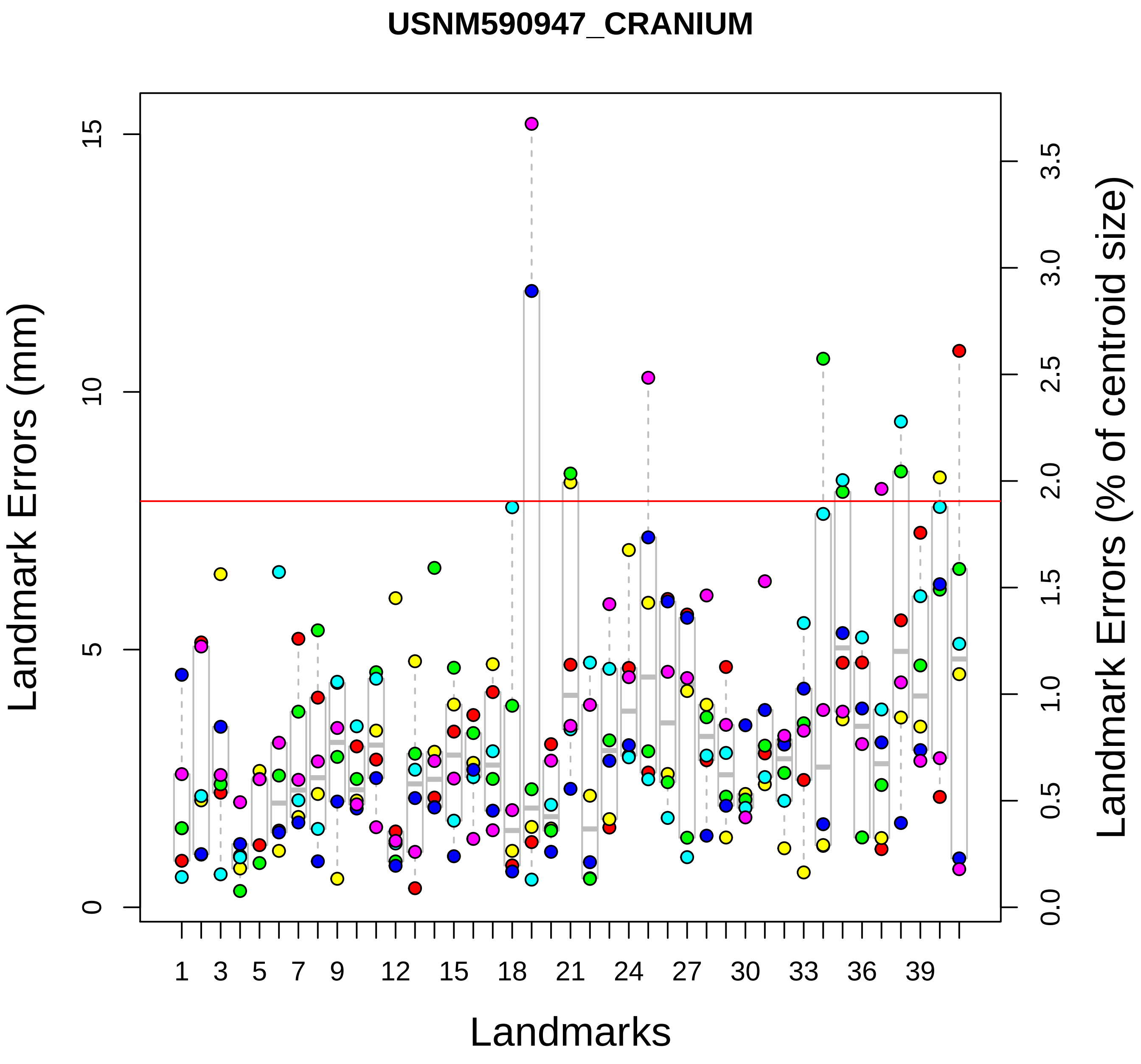
USNM590947_CRANIUM
Landmark Errors (mm)
Landmark Errors (% of centroid size)
Landmarks
0
5
10
15
0.0
0.5
1.0
1.5
2.0
2.5
3.0
3.5
1
3
5
7
9
12
15
18
21
24
27
30
33
36
39

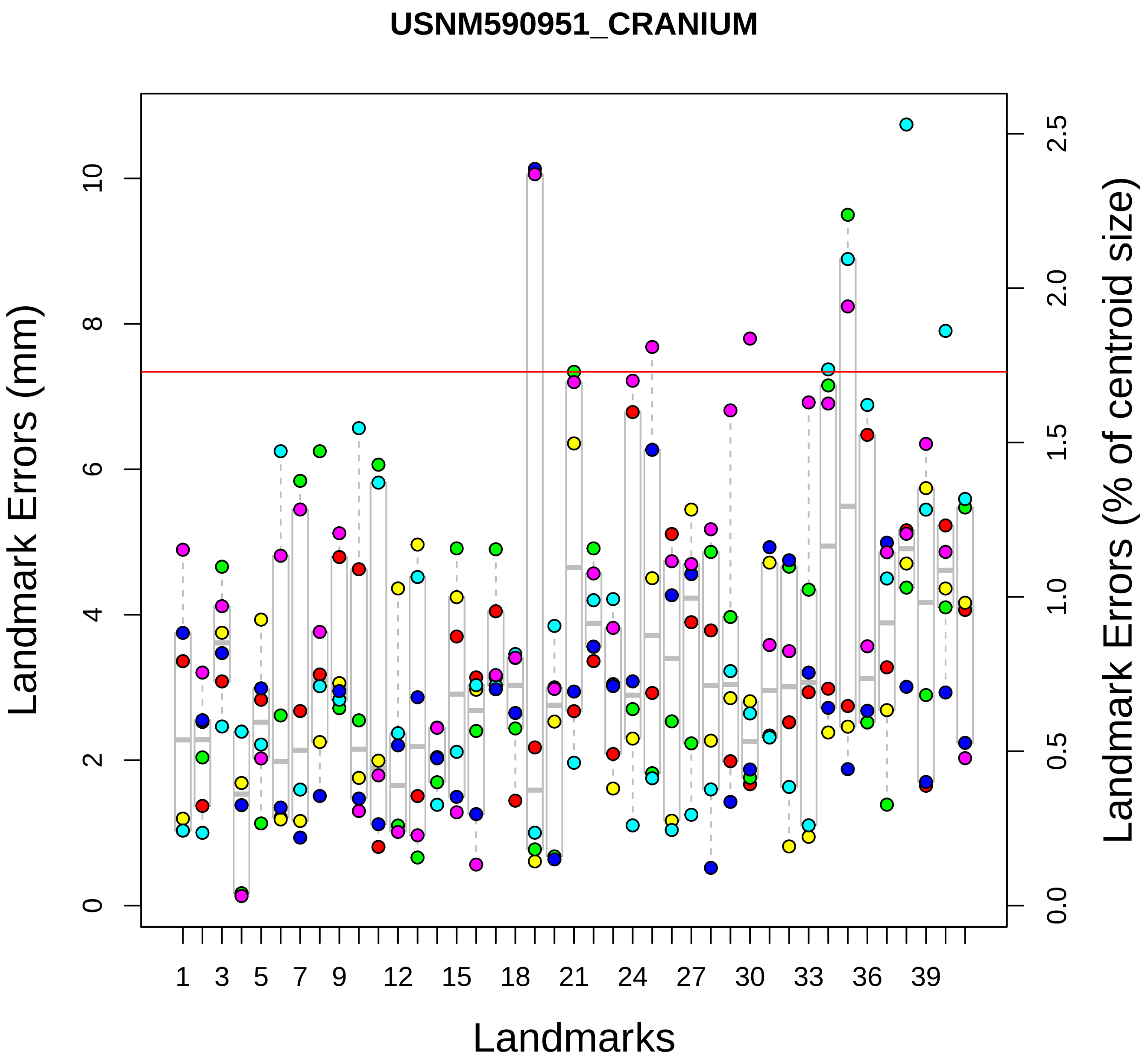
USNM590951_CRANIUM
Landmark Errors (mm)
Landmark Errors (% of centroid size)
Landmarks
0
2
4
6
8
10
0.0
0.5
1.0
1.5
2.0
2.5
1
3
5
7
9
12
15
18
21
24
27
30
33
36
39

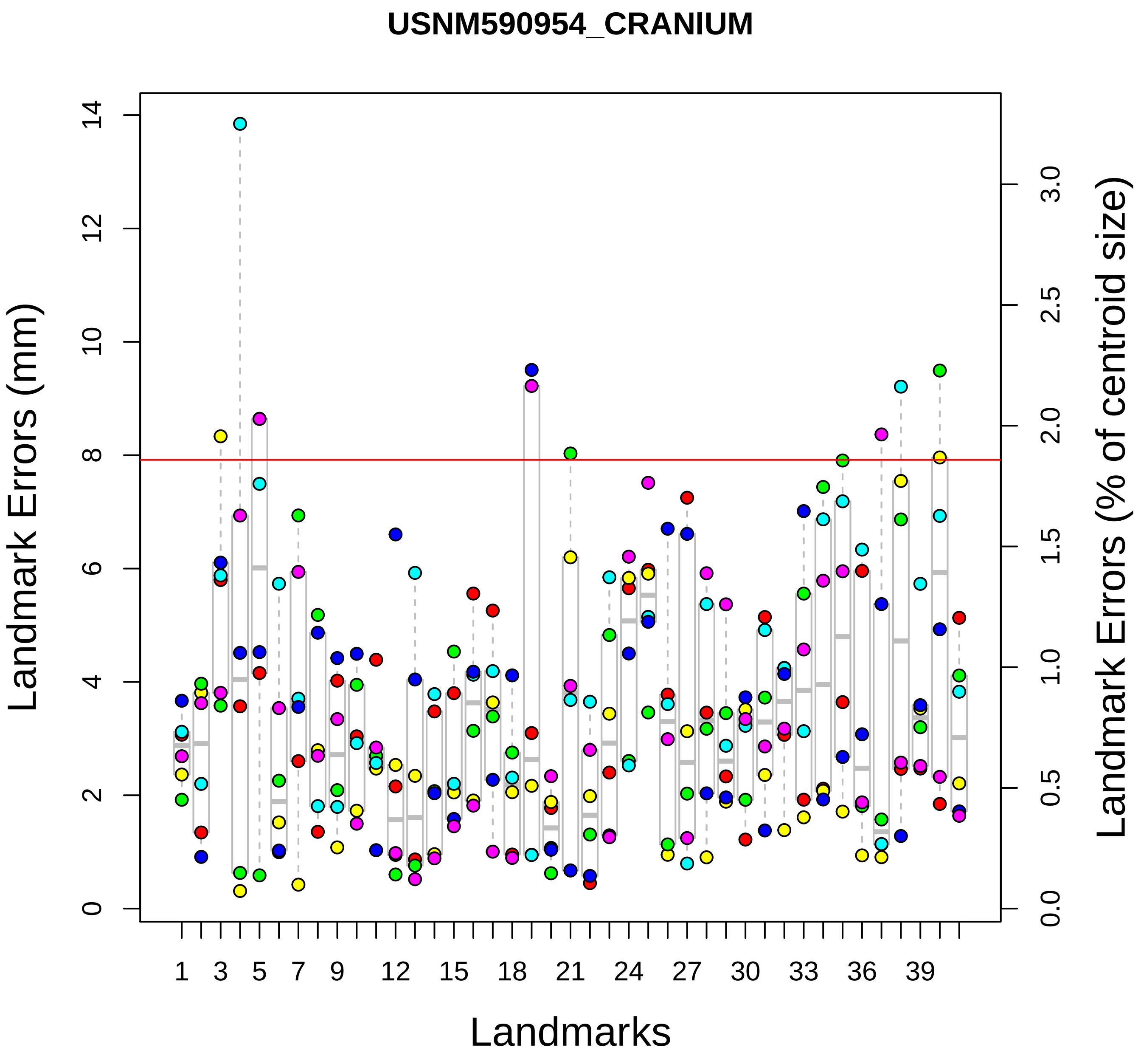

USNM590954_CRANIUM
Landmark Errors (mm)
Landmark Errors (% of centroid size)
Landmarks
0
2
4
6
8
10
12
14
0.0
0.5
1.0
1.5
2.0
2.5
3.0
1
3
5
7
9
12
15
18
21
24
27
30
33
36
39

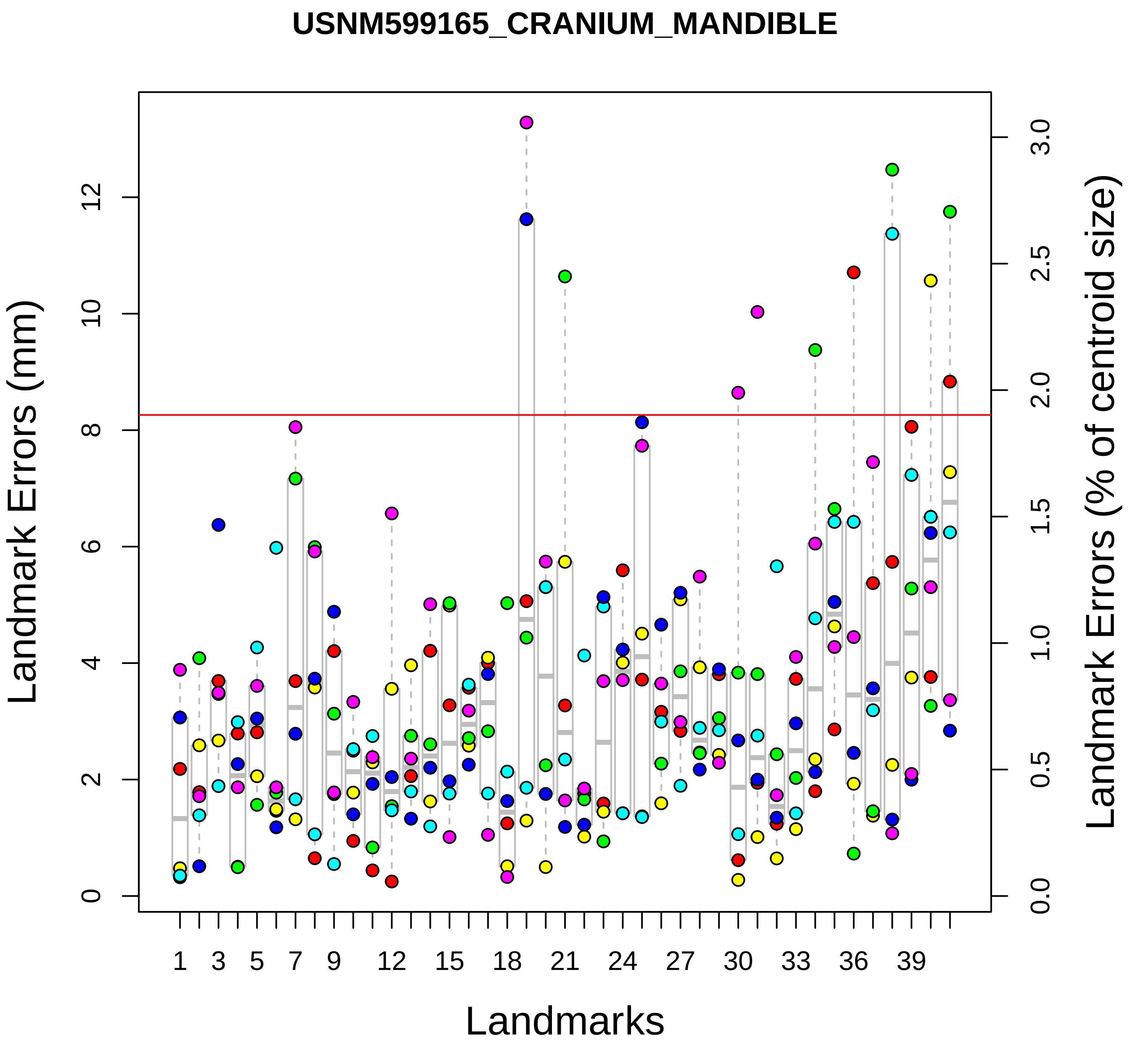

USNM599165_CRANIUM_MANDIBLE
Landmark Errors (mm)
Landmark Errors (% of centroid size)
Landmarks
0
2
4
6
8
10
12
0.0
0.5
1.0
1.5
2.0
2.5
3.0
1
3
5
7
9
12
15
18
21
24
27
30
33
36
39

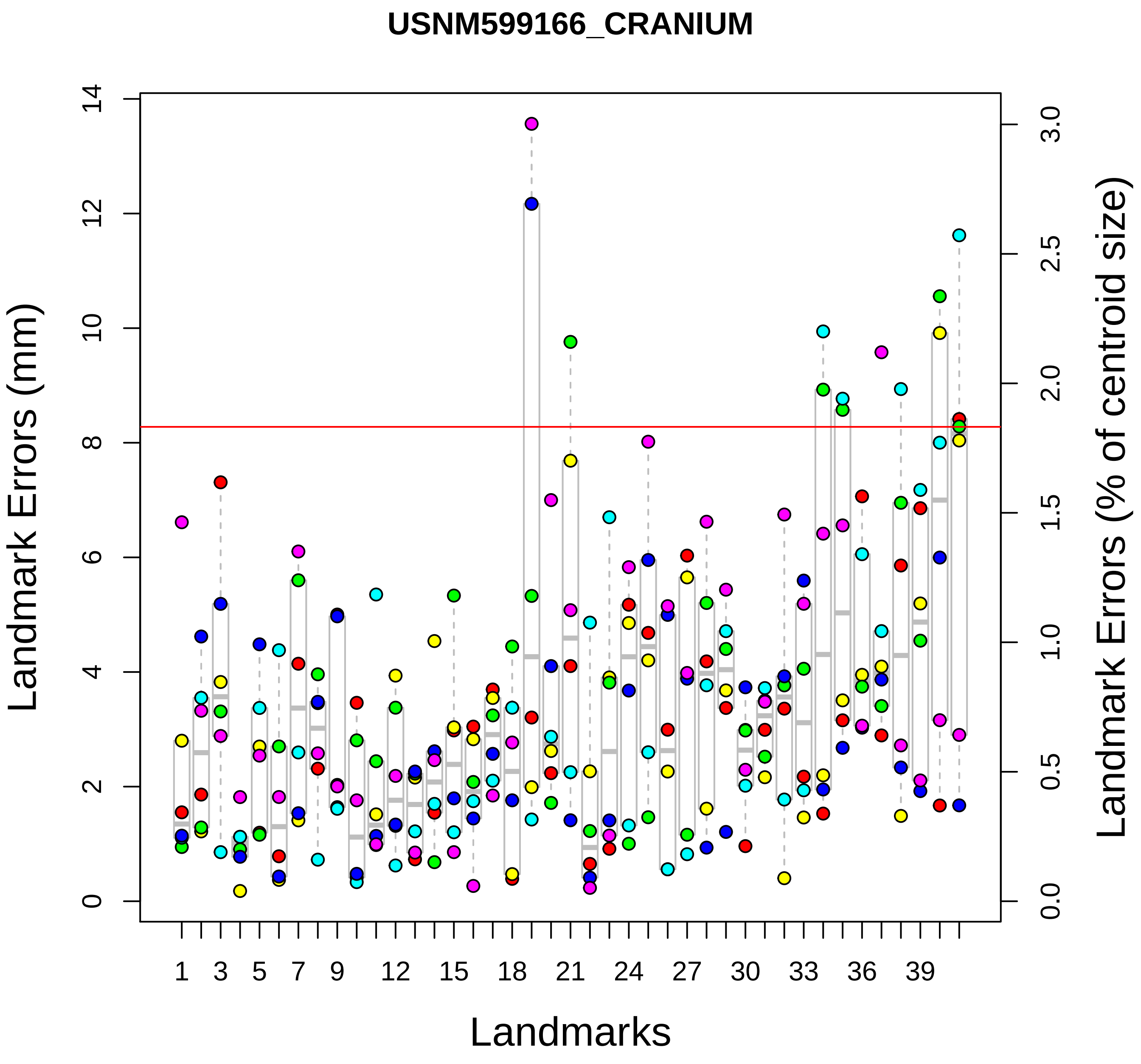

USNM599166_CRANIUM
Landmark Errors (mm)
Landmark Errors (% of centroid size)
Landmarks
0
2
4
6
8
10
12
14
0.0
0.5
1.0
1.5
2.0
2.5
3.0
1
3
5
7
9
12
15
18
21
24
27
30
33
36
39
