# Supplementary material for: Automated landmarking via multiple templates
Source: PLoS One. 2022 Dec 1;17(12):e0278035. doi: 10.1371/journal.pone.0278035 (PMC9714854; doi:10.1371/journal.pone.0278035)
Supplement: S1 Appendix — (DOCX) [file pone.0278035.s022.docx]

**S1 APPENDIX. STEP-BY-STEP INSTRUCTIONS FOR RUNNING MALPACA**

**I. Installation and sample data:**

The graphic user interface pipelines of MALPACA and K-means template selection have been incorporated into the ALPACA module of the SlicerMorph extension of the 3D Slicer biomedical imaging software. To install the SlicerMorph extension for Windows, MacOS, or Linux, please follow the instructions at <https://github.com/SlicerMorph/SlicerMorph#installation>. Users following these official methods will have all the necessary packages.

Introductions to the 3D Slicer UI and how to navigate between modules can be found at <https://slicer.readthedocs.io/en/latest/user_guide/user_interface.html>.

If it is the first time opening the ALPACA module, it may install additional python libraries necessary for running ALPACA. Tutorials for ALPACA can be found at <https://github.com/SlicerMorph/Tutorials/tree/main/ALPACA>.

Tutorials for MALPACA and K-means template selection method can also be found at: <https://github.com/SlicerMorph/Tutorials/tree/main/MALPACA>.

**Sample Data to run MALPACA:** Entire set of mouse skull models and associated manual LMs used in the study can be found at <https://github.com/SlicerMorph/mouse_models>. Please clone or download this repository as the remaining steps will use them as sample data. As noted in the data availability statement, Smithsonian Institution does not allow us to re-distribute the 3D models we have extracted from the CT scans of their ape collection. Please contact Smithsonian Institution Digitization Program Office (<https://dpo.si.edu/>) to obtain access to the ape skull scans.

Please follow the rest of the document to:

1. Run the K-means multi-template selection procedure. This step can be skipped, if the user already knows what samples are going to be used as templates.
2. Use the ALPACA in batch mode using the multi-template method.
3. Use the SlicerMorphR R package to do quality control on the MALPACA estimate. We provide an example script to detect, remove outlier and redo the estimates. This step is optional, and users can implement their own quality control pipelines using the example provided. The SlicerMorphR R package for MALPACA quality control is currently in its development version. It can be installed by using ‘devtools::install_github('chz31/SlicerMorphR')’. The script repository is at: https://github.com/chz31/SlicerMorphR.

**II. K-means multi-template selection (Optional Step)**

1. Switch to the “Templates Selection” tab (yellow). In the “Templates Selection Setup” collapsible section, specify entries to enable buttons in the following sections (S1 Appendix Fig 1):

- Models Directory (red): specify the folder that contains the original 3D models in “. ply” format. If you have downloaded the mouse models repository from github, this would be the contents of the ‘models’ folder.
- Point clouds output directory (green): specify the folder for storing downsampled point clouds for subsequent K-means multi-templates selection.
- Templates output directory (blue): specify the folder for storing the selected templates


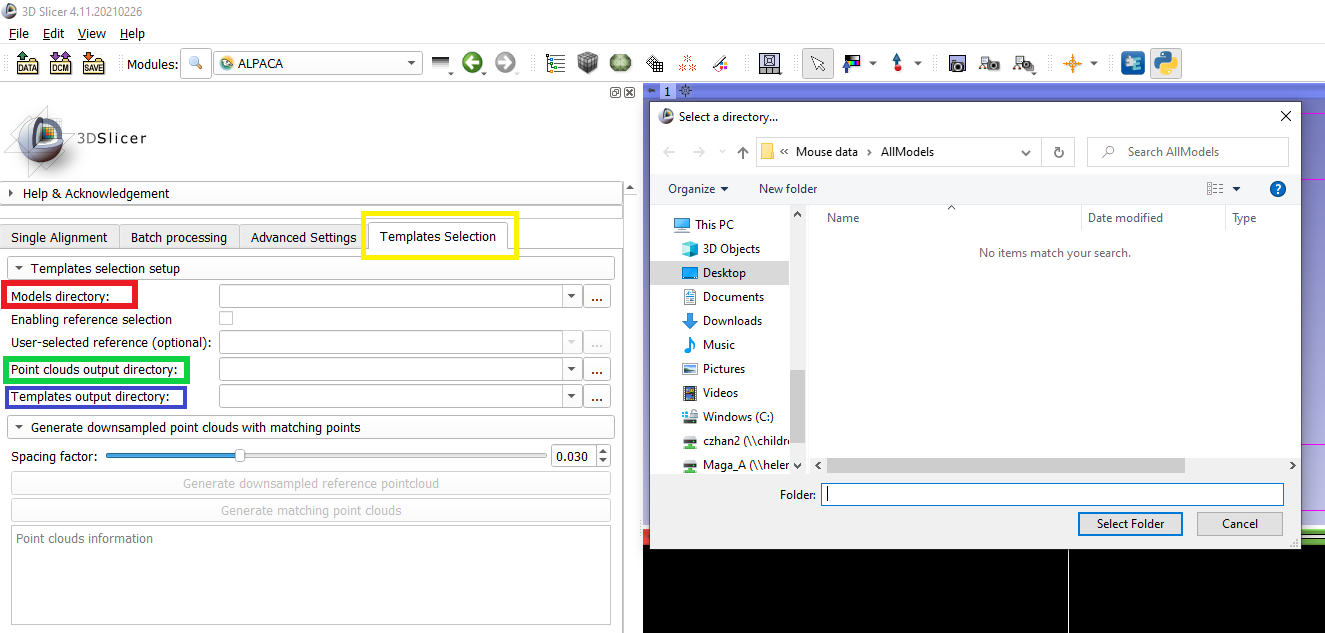


**S1 Appendix Fig 1. Set up templates selection.**

- (**Optional**) Checking the “Enabling reference selection” checkbox (red) will enable “User-selected reference” entry (S1 Appendix Fig 2). This allows users to select a specimen in the “Models Directory” (red arrow) as the reference for registering specimens as well as generating downsampled point clouds with point-to-point correspondence.
- If this checkbox is unchecked or no specific reference is selected, the first specimen in the “Models Directory” folder will serve as the reference by default.


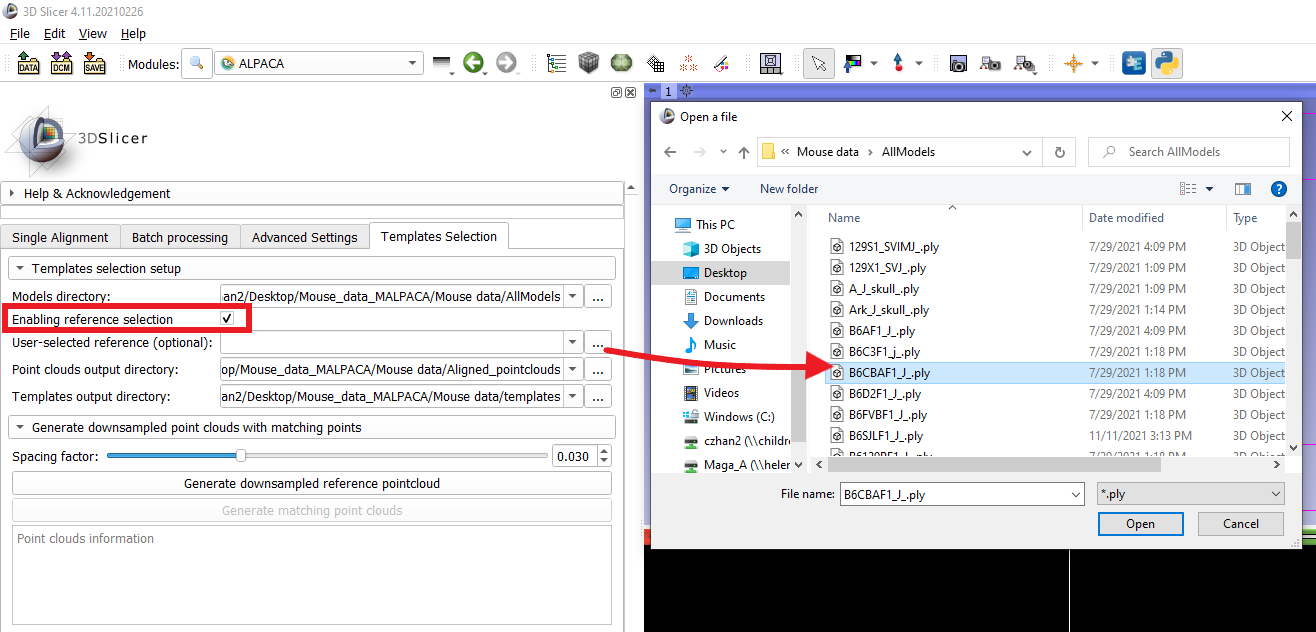


**S1 Appendix Fig 2. Optional reference selection for matching pointclouds.**

2. Generating downsampled point clouds with point-to-point correspondence.

- Specify a “Spacing factor” value using the slider (red) or simply input a value in the box at the right side (S1 Appendix Fig 3). This value determines how sparse the downsampled reference point cloud will be. The larger the spacing factor value, the sparser the point cloud will be. For new samples, this requires some experimentation on user’s end (See explanation in next steps). For mouse sample spacing of 0.03 is suggested.
- Click the “Generate downsampled reference pointcloud” button (blue) (S1 Appendix Fig C). After the downsampled reference point cloud is generated, the box below (green) will display information about the name of the reference model and the number of points in the reference point cloud. The “Generate matching point clouds” button will also be enabled.


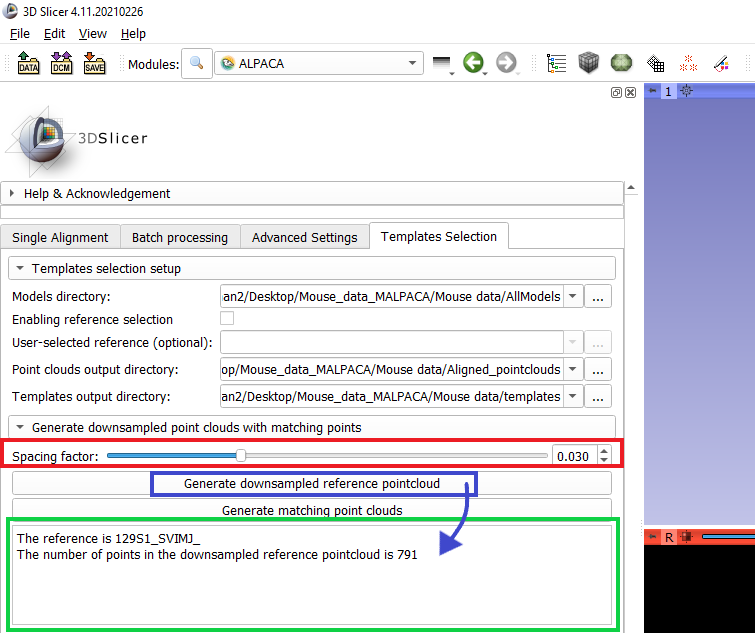


**S1 Appendix Fig 3. Generate downsampled reference pointcloud.**

- Click the “generate matching point clouds” button (“red”) (S1 Appendix Fig 4). This will extract point clouds from each target specimen listed in the “Models directory” based on the reference. These point clouds with point-to-point correspondence will be stored in “fcsv” format as landmark files in the folder specified in 2) of Step 1.
- Note that occasionally a point in the target may be repeatedly fetched if it is equally close to multiple points in the reference point cloud. Consequently, the number of unique points in a target will be smaller than that of the reference.
- Since the number of points in a point cloud is usually large, having a few redundant points are unlikely to significantly impact results of morphometric analysis. Thus, the error threshold that measures the number of redundant points in a target point cloud is set up as 1%. For example, if the reference has 800 points and the number of unique points in the target is 793, the result will be suggested as acceptable (the error rate does not exceed the 1% threshold).
- The box below the “Generate matching point clouds” button displays information about the number of unique points in generated point clouds, including specimens with number of unique points smaller than that of the reference and whether their error rates exceed the threshold.
- Users can opt to ignore the warning when some point clouds have error rates exceeding 1%. If not, they can try increasing the spacing factor to achieve sparser point clouds or check possible deficiencies in the original models.


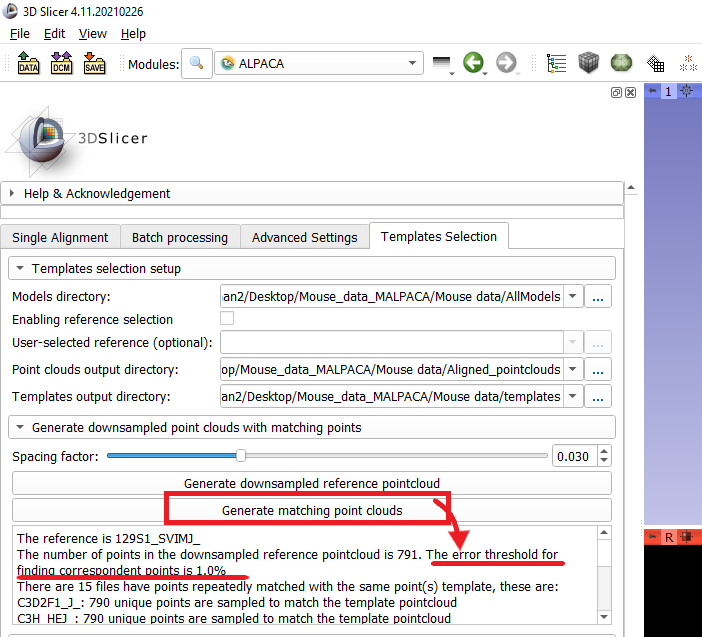


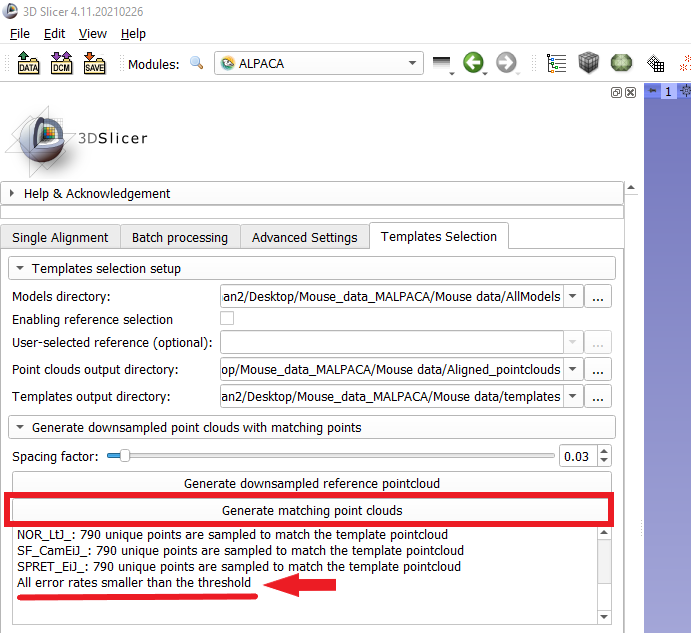


**S1 Appendix Fig 4. Generate matching pointclouds.**

3. Specify whether there are multiple groups within the sample in the “Multi-template selection” section. If users have the fcsv files of the point cloud data in hand, they can specify the directory in the “Point clouds output directory” in Step 1 without re-generating these point clouds.

- Check “One group for the whole sample” (red arrow) if no pre-known groups exist within the sample or users do not want to divide the sample into groups (S1 Appendix Fig 5). In this case, the user-input “Number of templates per group” (blue) is the number of templates for the whole sample.


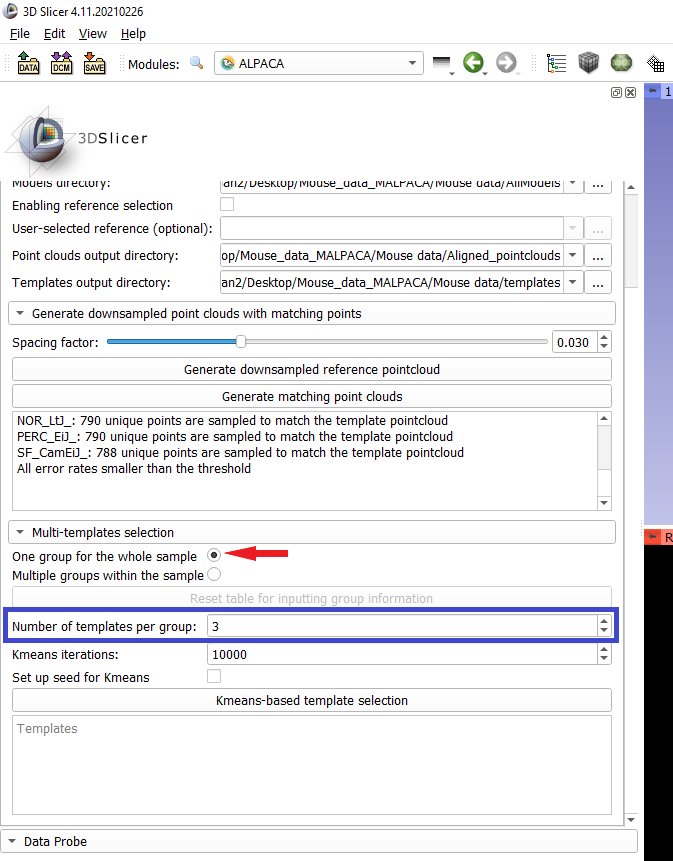


**S1 Appendix Fig 5. Specify the number of templates.**

- Check the “Multiple groups within sample” (green arrow) if for dividing a sample into multiple groups based on prior knowledge (S1 Appendix Fig 6). By checking this option:
- A table for manually entering group labels for each specimen will be generated (right). A K-means algorithm will be executed per group to select a user-defined number of templates specified in “Number of Templates per group” (blue).
- The “Reset table for inputting group information” button (yellow) will be enabled. Click it to reset the group information table.


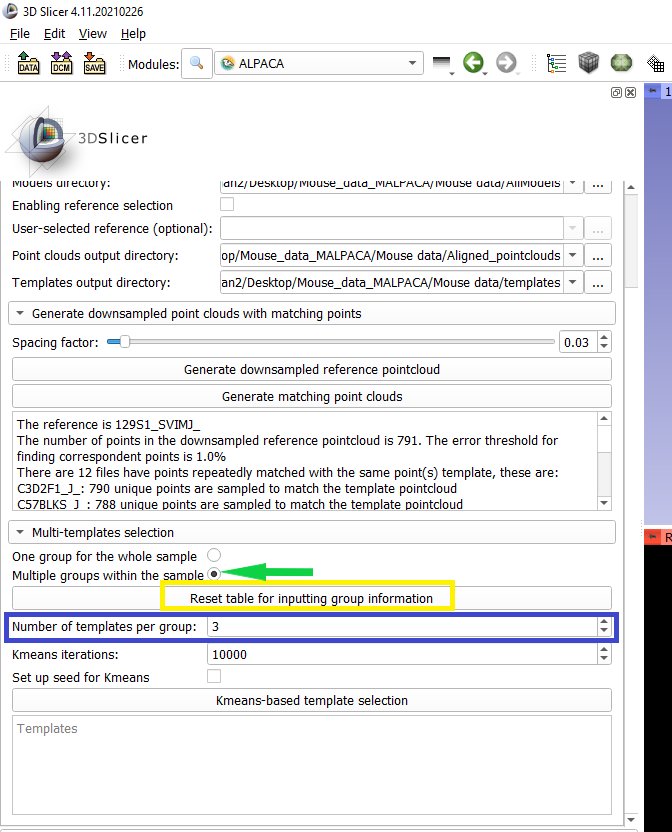

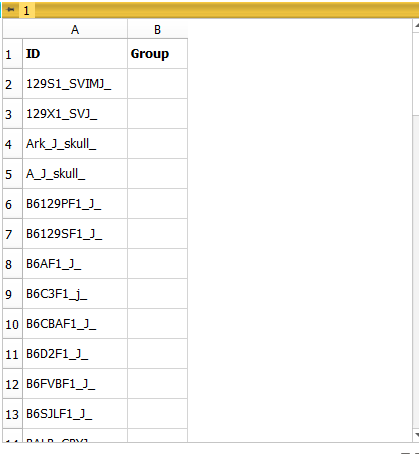


**S1 Appendix Fig 6.** **Specify desired cluster numbers for K-means.**

4. Set up number of iterations (red) and a seed (optional) (yellow) for K-means algorithm (S1 Appendix Fig 7)

- The default number of K-means iterations is 10,000. Iteration number can be increased for consistent results of K-means.
- For ensuring reproducibility, users can set up a seed for K-means by checking the “Set up seed for K-means” box (blue) (S1 Appendix Fig 7). However, this may also influence other random-based functions in 3D Slicers. Therefore, for running other 3D Slicer modules, it is recommended to open a new Slicer session.


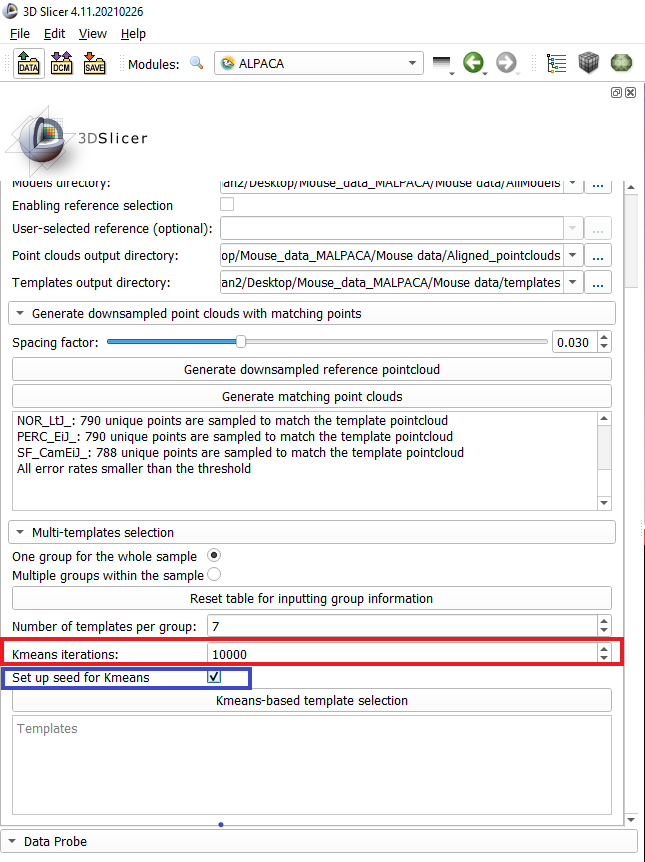


**S1 Appendix Fig 7. Set up K-means.**

5, Click “Kmeans-based template selection” button (red) will execute K-means algorithm for multi-template selection (S1 Appendix Fig 8). The models selected as templates will be saved in the folder specified in “Templates output directory” in Step 1. The display box (yellow) below shows specimens selected as the templates.


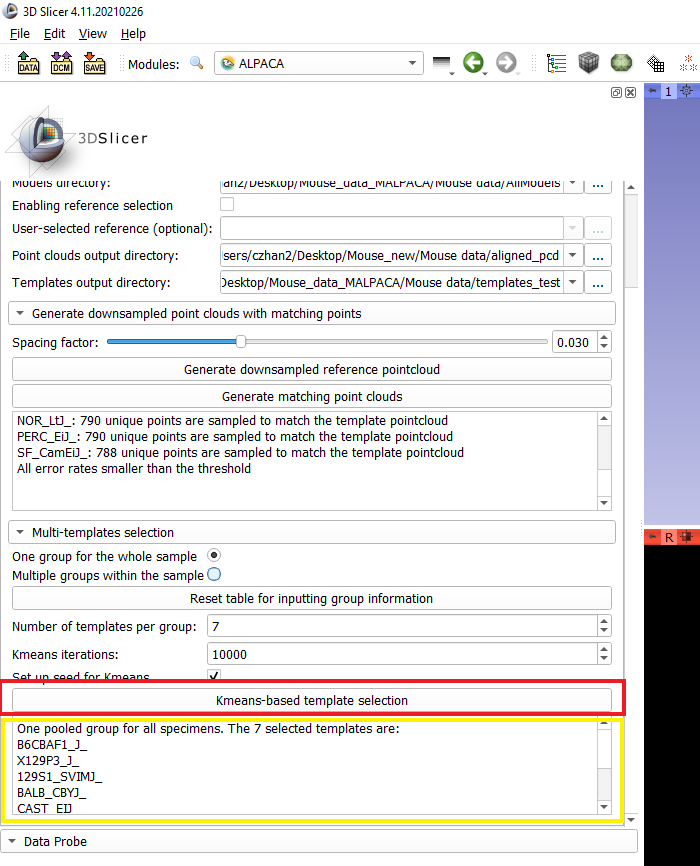


**S1 Appendix Fig 8. Run K-means multi-template selection.**

- If “One group for the whole sample” is checked in step 4, a plot of PC1 and PC 2 based on a Generalized Procrustes Analysis of the point cloud data will be displayed (S1 Appendix Fig 9). Specimens are black squares. Templates are light color, diamond-shaped points that overlay the squares.


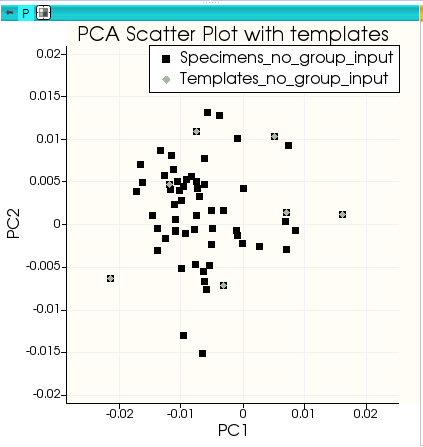


**S1 Appendix Fig 9. PCA plot for templates selection results (one group for the whole sample).**

- If “Multiple groups within the sample” is checked (green arrow) and group identity for each specimen is entered, the display box (yellow) will show selected templates for each entered group (S1 Appendix Fig 10). The PC plot will also display specimens in each user-defined group in a unique color.


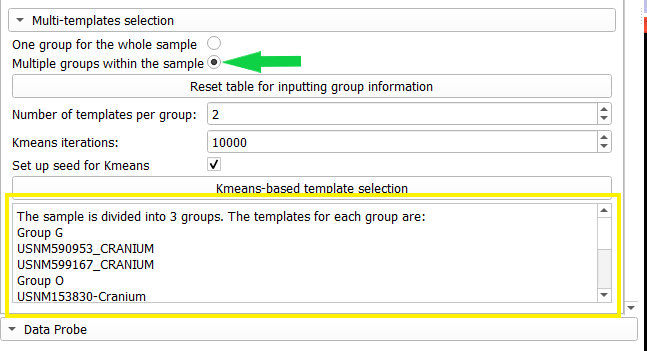


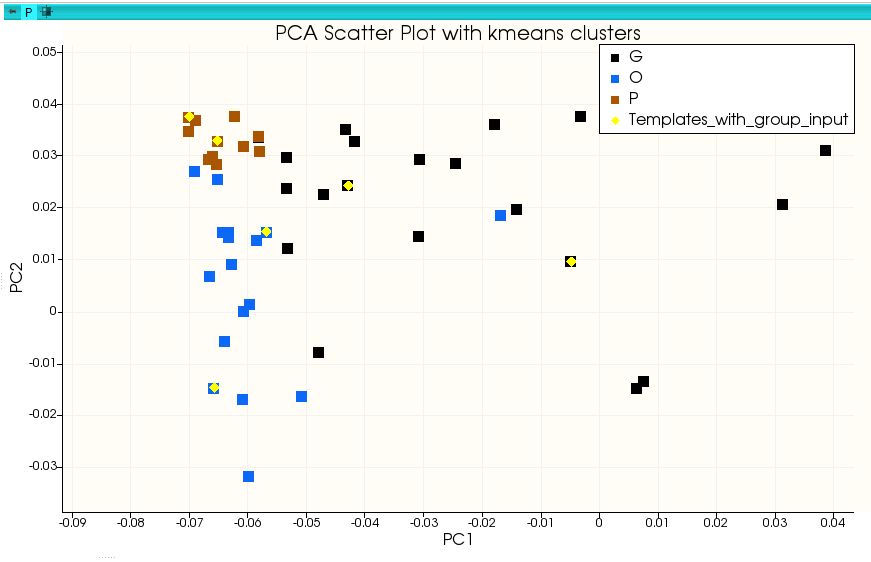


**S1 Appendix Fig 10. Templates selection results when multiple groups within the sample.**

**III. Executing ALPACA in Multi-Template mode (MALPACA)**

6. Switch to the ALPCA module and choose the Batch Processing tab (red). In the “Method” entry (dark blue), open the drop-down menu to select **“Multi-Template (MALPACA)”** option from the dropdown menu (S1 Appendix Fig 11).

7. Select required input and output directories (S1 Appendix Fig. 11).

- In the “Source mesh(es)” entry (yellow), select the folder that contains .ply files of the templates.
- In the “Source landmarks” entry (green), select the folder that contains fcsv landmark files for the templates. The names of the template model and landmark files must be identical.
- In the “Target mesh directory” entry (dark grey), select the folder that contains .ply files of the target meshes. These are the specimens to be landmarked by MALPCA.
- In the “Target output landmark directory” (light blue), select the folder for MALPACA output.

8. Click the “Run auto-landmarking” button (red arrow) to execute MALPACA. Slicer may appear to be in the “no response” condition (S1 Appendix Fig 11). This is because MALPACA is running.


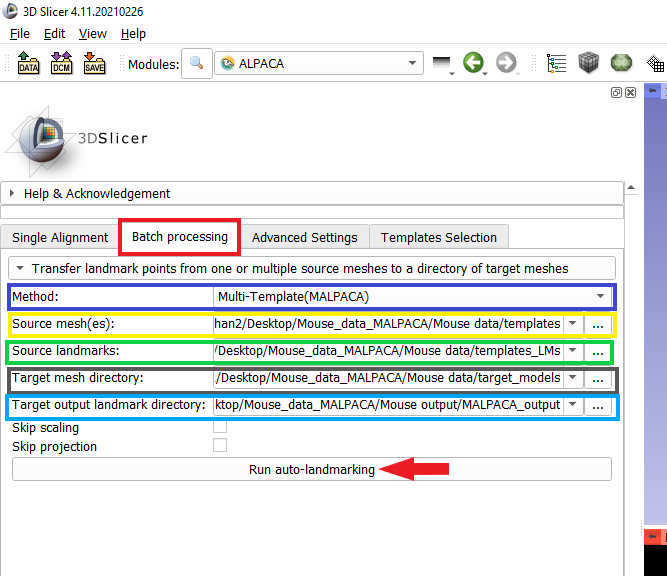


**S1 Appendix Fig 11. Set up and running MALPACA.**

9. Open the target output landmark directory specified in Step 7 that stores the MALPACA output (S1 Appendix Fig 12).

- The “advancedparameters.txt” file stores the MALPACA settings.


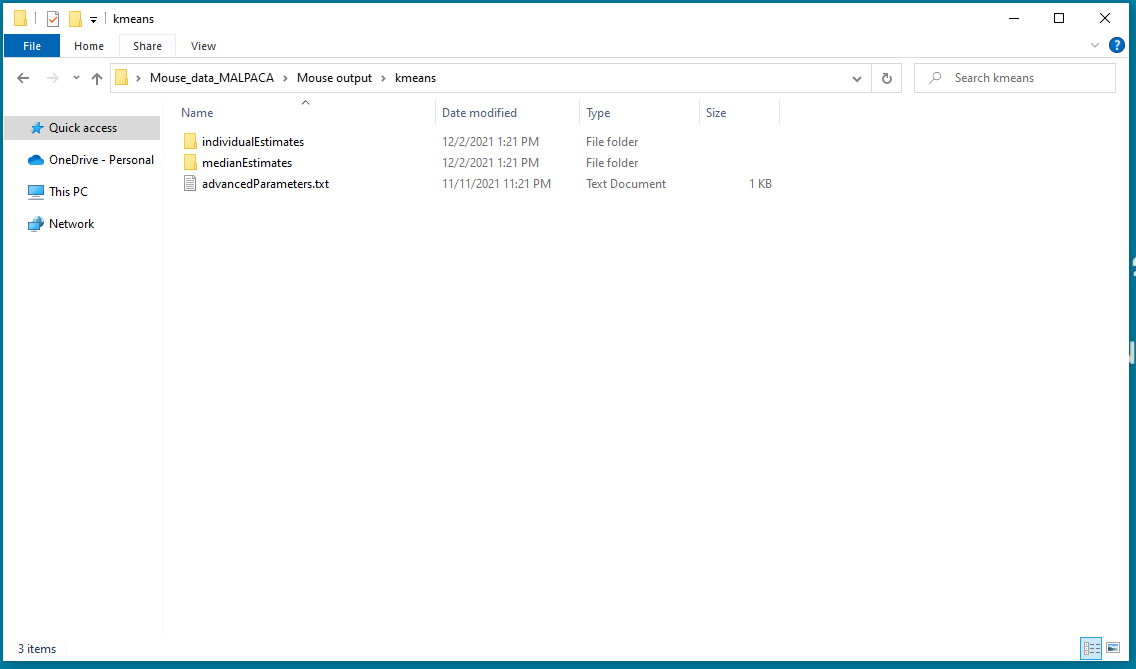


**S1 Appendix Fig 12. MALPACA output folders.**

- The “individual estimates” folder contains landmarks estimated by each individual template stored in the fcsv format (S1 Appendix Fig 13 - 14). For each file name, the postfix is the template that generate this estimated landmark file. For example, “129X1_SVJ_B6CBAF1” suggests that the estimated landmarks of the specimen 129X1_SVJ is derived from using the template B6CBAF1.


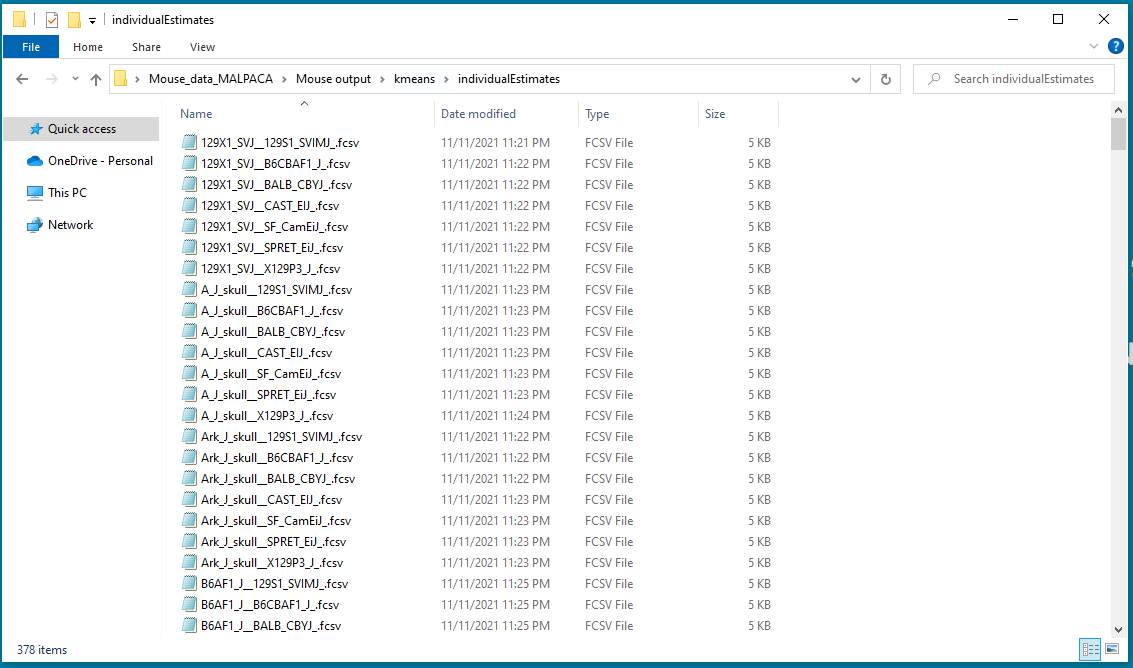


**S1 Appendix Fig 13. Individual estimates files.**

- The “medianEstimates” folder contains the final output of MALPACA storing in fcsv format (S1 Appendix Fig 14). Each landmark file name has a suffix “_median”, suggesting it is the median of the estimates derived from using all templates.


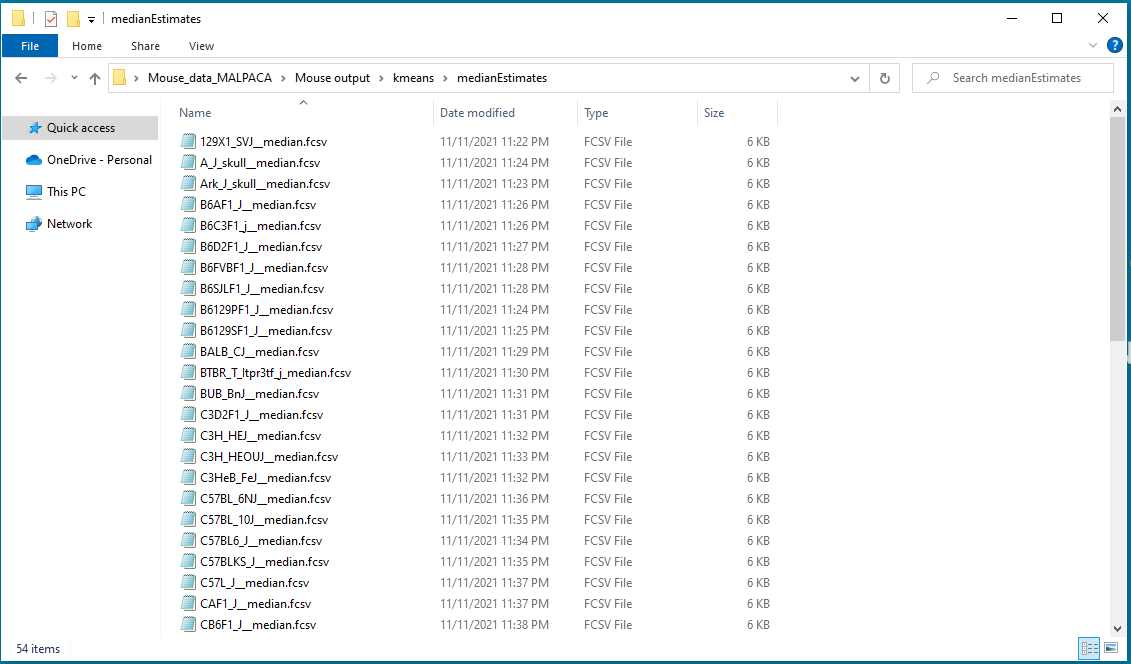


**S21 Fig 14. Median estimates files.**

**IV. Landmarking Quality Control Pipeline (OPTIONAL)**

One of the benefits of multi-template pipeline is the ability to assess confidence in the estimates. Here we demonstrate a pipeline based on a simple heuristic (mean RMSE + 2.SD RMSE) to flag individual estimates as potential outliers. It should be noted that because MALPACA is using median to obtain final LM positions, it is robust to an occasional outlier (i.e., removal of the outlier and re-estimate of the position should have no appreciable effect on final LM estimate). However, in cases where an ill-chosen template produces consistently poor results, diagnostic plots like the one shown below would be helpful to identify the problem (abundance of LMs above threshold for that template) and re-run the MALPACA pipeline by replacing the template by another sample, or simply re-estimating without the outlier.

10. Open R or RStudio console, install SlicerMorphR package using the code devtools::install_github(‘chz31/SlicerMorphR'), then run ‘library(SlicerMorphR)’ to load the package.

11. To try the functions for extracting outliers, users can load the ape MALPACA data from Github, either by clone the repository or using the following code to download the zip file and unzip it:

- save.dir='C:/tmp' #enter your directory to download the MALPACA data sample
- setwd(save.dir)
- zip_url <- "https://github.com/SlicerMorph/Mouse_Models/archive/refs/heads/main.zip"
- download.file(url = zip_url, destfile = "Mouse_Models.zip")
- unzip(zipfile = "Mouse_Models.zip")

The following steps use the sample ape data loaded in Step 3. Users can switch to their own data.

12. Set up MALPACA output path and the directory that stores the template landmark or model files (specified in step 7 if using your own data):

- MAL_outDir_git<- paste(save.dir, 'Mouse_Models-main/ape_malpaca', sep="/") #Set up MALPACA output path
- templates_path_git<- paste(save.dir, 'Mouse_Models-main/ape_malpaca/templates_LMs', sep='/') #Set up

13. Read all median estimates and estimates derived from individual templates.

- LMs <- read.malpaca.estimates(MALPACA_outputDir = MAL_outDir_git, templates_Dir = templates_path_git)
- allEst <- LMs$allEstimates #Store all individual estimates in a 4D array [i, j, k, n]. i = number of templates; j = dimension; k = number of templates; n = sample size. For example:


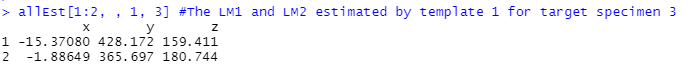


- MAL_medians <- LMs$MALPACA_medians #Store all median estimates in a 3D array [i, j, n]. i = number of templates; j = dimension; n = sample size. For example:


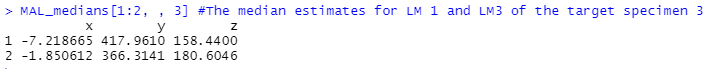


14. Use the ‘extract.outliers()’ function in SlicerMorphR to find and remove outliers from estimates derived from individual templates. This function calculates the distance between each landmark estimated by an individual template to its corresponding MALPACA median estimate. If the distance exceeds the threshold, then this estimate is marked as an outlier. By default, the threshold is 2 × (standard deviation) + mean of the pooled distances for each specimen. ‘NA’ is then assigned to its coordinates in the 4D array that stores all estimates of individual templates. For the parameters:

- ‘allEstimates’ is the 4D array that stores all estimates of individual templates, generated by ‘read.malpca.estimates()’
- ‘MALPACA_medians’ is the 3D array that stores MALPACA median estimates generated by ‘read.malpaca.estimates()’.
- ‘outputPath’ specifies the output directory for storing output the pdf files that contains the boxplots of distances between each individual estimate to its corresponding MALPACA median estimate.
- ‘ZScore’ specify the threshold for determining outliers. The default value is 2. This means that the threshold is (2 (standard deviation) + mean) of pooled distances between all pairs of individual estimates and the corresponding MALPACA median estimates.
- For the ape sample data, run:
  - ‘outliers <- extract.outliers(allEstimates = allEst, MALPACA_medians = MAL_medians, outputPath = outPath)’
- Results using the ape sample data are shown below (S1 Appendix Fig 15 - 16). The boxplot shows the distances between every individual landmark estimate and its corresponding MALPACA median output for one ape specimen. Each column of dots represents the distances for the estimates of the six ape templates. The red horizontal line represents the threshold. Points above the threshold are outlier estimates. For the pdf file in the ‘outputPath’, each page represents a boxplot for one target specimen. The legends are at the first page.


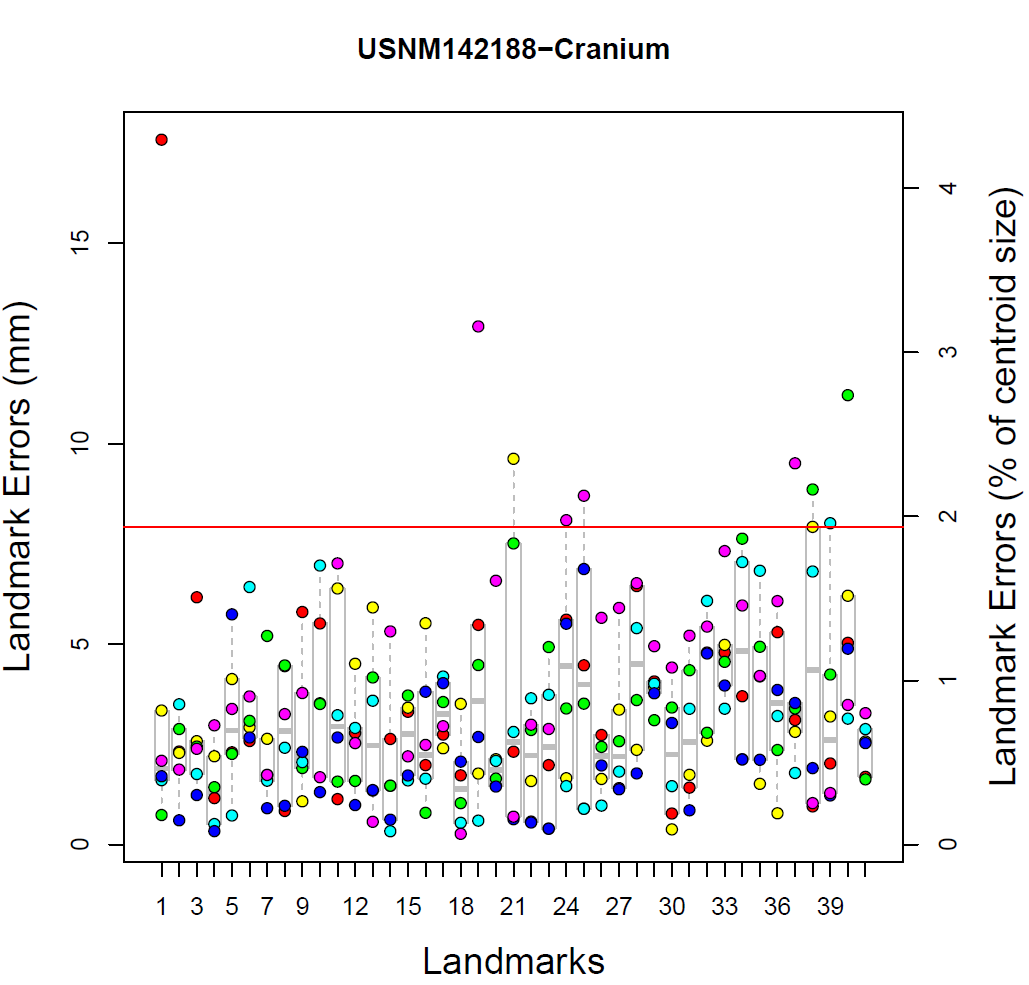


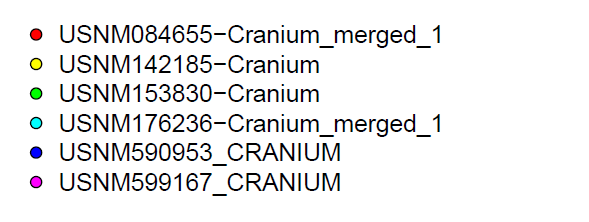


**S1 Appendix Fig 15. Sample output of boxplots that denote individual estimates, the heuristic threshold, and outlier estimates.**


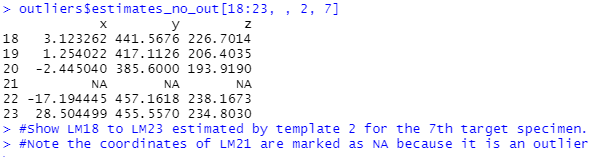


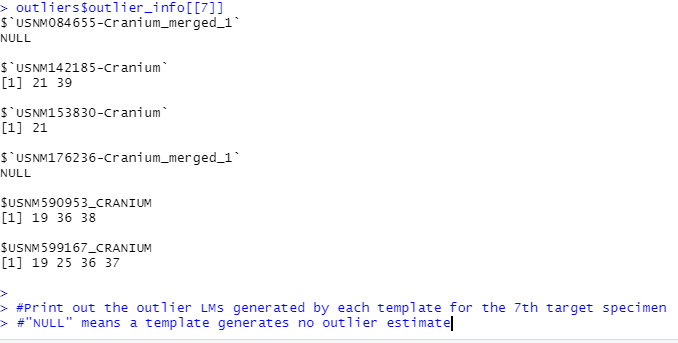


**S1 Appendix** **Fig 16. Outlier information.**

15. Use the “get.medians()” function from SlicerMorphR to generate new median estimates for each specimen, storing in a 3D array [i, j, n]. i = number of templates; j = dimension; n = sample size.

- medians <- get.medians(AllLMs = outliers$estimates_no_out, outlier.NA = TRUE)
- #AllLMs = input 4D array that stores estimates of individual templates
- #outlier.NA by default is False. Setting up ‘True’ specifies there is NA in the input 4D array.
